# Novel isobavachalcone derivatives induce apoptosis and necroptosis in human non-small cell lung cancer H1975 cells

**DOI:** 10.1080/14756366.2023.2292006

**Published:** 2023-12-12

**Authors:** Jie Chen, Long Zhao, Meng-Fan Xu, Di Huang, Xiao-Long Sun, Yu-Xin Zhang, Hong-Mei Li, Cheng-Zhu Wu

**Affiliations:** a School of Pharmacy, Bengbu Medical College, Bengbu, Anhui, China; b Anhui Province Biochemical Pharmaceutical Engineering Technology Research Center, Bengbu, Anhui, China; c School of Laboratory Medicine, Bengbu Medical College, Bengbu, Anhui, China

**Keywords:** Isobavachalcone derivatives, NSCLC, apoptosis, necroptosis, RIP3

## Abstract

In this study, seventeen isobavachalcone (IBC) derivatives (1–17) were synthesised, and evaluated for their cytotoxic activity against three human lung cancer cell lines. Among these derivatives, compound 16 displayed the most potent cytotoxic activity against H1975 and A549 cells, with IC50 values of 4.35 and 14.21 μM, respectively. Compared with IBC, compound 16 exhibited up to 4.11-fold enhancement of cytotoxic activity on human non-small cell lung cancer H1975 cells. In addition, we found that compound 16 suppressed H1975 cells via inducing apoptosis and necroptosis. The initial mechanism of compound 16 induced cell death in H1975 cells involves the increasing of Bax/Bcl-2 ratio and Cyt C protein level, down-regulating of Akt protein level, and cleaving caspase-9 and -3 induced apoptosis; the up-regulation of RIP3, p-RIP3, MLKL, and p-MLKL levels induced necroptosis. Moreover, compound 16 also caused mitochondrial dysfunction, thereby decreasing cellular ATP levels, and resulting in excessive reactive oxygen species (ROS) accumulation.

## Graphical Abstract

**Figure:**
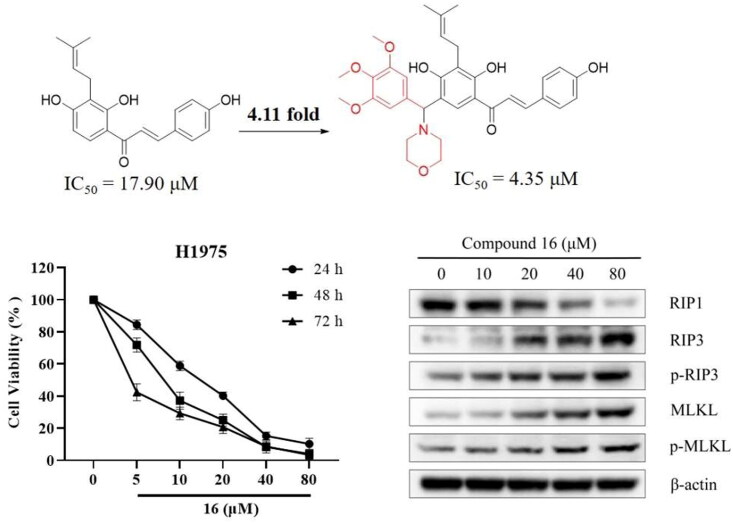

## Introduction

Lung cancer is one of the most common causes of cancer morbidity and mortality worldwide^1^. Non-small cell lung cancer (NSCLC) is the most frequent subtype of lung cancer, accounts for approximately 80–85% of all lung cancers^2^. Commonly, NSCLC is not detected until the disease has progressed to an advanced state^3^. Up to now, surgery, radiation, chemotherapy, molecularly targeted therapy, and immunotherapy are used to treat NSCLC^4^. Chemotherapy or tyrosine kinase inhibitors (TKIs) improve the median survival of patients with advanced NSCLC, but the overall survival remains poor^5^^,^^6^. However, with the progress of chemotherapy, TKIs do not have a good efficacy in the epidermal growth factor receptor (EGFR) mutation type of NSCLC, and it is inevitable that acquired drug resistance will occur, resulting in chemotherapy failure^7^.

Programmed cell death (PCD), including apoptosis, autophagy, necroptosis, ferroptosis, pyrotosis, etc., refers to the form of cell death that can be regulated by various molecules or signals, which is different from necrosis^8^^,^^9^. Many studies showed that the discovery of small-molecule compounds targeting different forms of PCD has been emerging as a new strategy for many types of human cancers^10–14^. Recently, we reported that isobavachalcone (IBC) induces multiple PCD in human triple-negative breast cancer MDA-MB-231 cells, including necroptosis^15^.

Necroptosis, a type of PCD was identified in 2005, which is distinct from classical apoptosis and necrosis^16^. Until now, many necroptosis inducers have been emerging as new anti-cancer agents. Shikonin induces necroptosis in glioma cells via promoting RIP1/RIP3 necrosome formation and ROS overproduction^17^. Matrine induces necroptosis in cholangiocarcinoma cells by enhancing RIP3 expression and the following RIP3/MLKL/ROS signalling pathway^18^. LGH00168 induces necroptosis in human lung cancer A549 cells by the ROS-mediated ER stress and NF-κB inhibition^19^. 2-Methoxy-6-acetyl-7-methyljuglone induces necroptosis in lung cancer cells by targeting RIP1 and ROS in a TNFα-independent manner^20^. ZZW-115 induces necroptosis in hepatocellular carcinoma cells via concomitant mitochondrial metabolism failure that triggers lower ATP production^21^. However, the anti-cancer properties of IBC and its analogs in necroptosis of H1975 cells are not completely understood.

IBC is a chalcone bearing prenyl group that was first isolated from the seed of Psoralea corylifolia L. in 1968^22^. Many research findings revealed that chalcones could be served as a common scaffold (e.g. xanthohumol, panduretin A, and licochalcones) for anti-cancer agent research and development^23–26^. To improve the anti-cancer activity of IBC, in this study seventeen IBC derivatives were synthesised and investigated cytotoxicity against three lung cancer cell lines. Furthermore, we demonstrated that the mechanism action of compound 16 induces apoptosis and necroptosis in H1975 cells.

## Results

### Semisynthesis of isobavachalcone derivatives

**Scheme 1.**
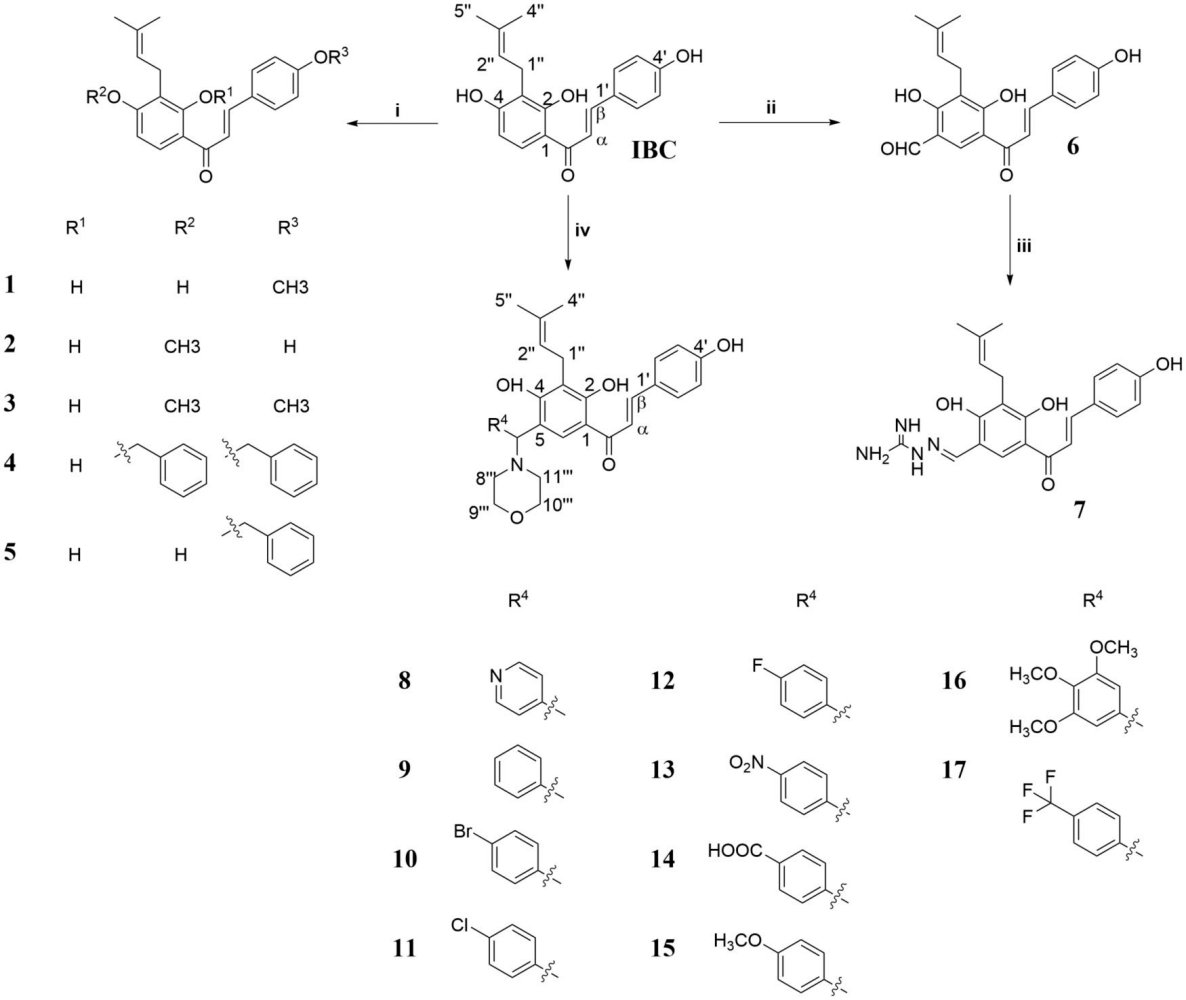
Synthetic route for IBC derivatives. (i) DMF, CH_3_I or benzyl chloride, Na_2_CO_3_, 65 °C, 10–12 h. (ii) 25%NaOH, CHCl_3_, 30 °C, 9 h. (ii) Acetic acid, aminoguanidine carbonate, 50 °C, 5 h. (iv) MeCN, aldehydes, morpholine, 80 °C, 12 h.

As shown in Scheme 1, three series of IBC derivatives (1–17) were designed and synthesised by using IBC as the starting material. Compounds 1–5 were synthesised from IBC through Williamson reaction in the presence of Na2CO3 in DMF. An aldehyde group was substituted on IBC afforded intermediate 6. Next, compound 6 was further reacted with aminoguanidine bicarbonate in the presence of catalytic amounts of acetic acid to provide compound 7. Finally, the target compounds with the structures of 8–17 were synthesised by the Mannich reaction of IBC with appropriately substituted benzaldehyde and morpholine in MeCN.

### In vitro *cytotoxic activity of IBC derivatives*

Compounds 1–17 were initially evaluated for their cytotoxic activities against the three human lung cancer cell lines (H1975, A549, and PC9) using the MTT assay (Table 1). Among these, compounds 7 and 16 showed excellent cytotoxicity against the human lung cancer (H1975 and A549) cell lines, with IC50 values of 4.35–15.04 μM. Compared to IBC, compound 16 displayed 4.11- and 1.53-fold enhancement of the cytotoxic effects on H1975 and A549 cells. We observed that both 2-OH and 4′-OH substituted IBC derivatives were inactive at 50 μM. Compounds 6 and 7, which were substituted with the aldehyde group and aminoguanidine at C-5 position, showed better cytotoxic activity against the H1975 cells than the parent compound. In addition, compounds 10, 13, 16, and 17, Mannich base substituted IBC derivatives, had potent cytotoxic activity against H1975 cells, with IC50 values of 6.76, 7.39, 4.35, and 7.01 μM, respectively. In addition, compound 16 exhibited relatively low cytotoxic activity against normal mouse hepatocytes Aml-12 cells, with IC_50_ values of 10.09 μM, indicating its selective inhibition (SI) to cancer cells (Table 2).

**Table 1.** Cytotoxic activity of compounds **1–17** and IBC (IC_50_, μM, 72 h).

Compound	H1975	A549	PC9
1	37.97	41.56	14.28
2	40.48	>50	35.31
3	>50	>50	>50
4	>50	>50	>50
5	13.59	33.12	24.86
6	5.79	24.06	18.36
7	7.33	15.04	8.30
8	13.79	39.22	29.92
9	12.10	26.95	22.81
10	6.76	34.75	18.56
11	11.44	21.84	19.65
12	12.11	32.95	20.66
13	7.39	44.54	23.54
14	23.64	>50	>50
15	11.74	28.86	23.19
16	4.35	14.21	16.12
17	7.01	30.13	20.58
IBC	17.90	21.69	9.22
Paclitaxel^a^	0.01	1.61	0.06

^a^Positive control.

**Table 2.** Cytotoxic activity of compound **16** on Aml-12 cells for 72 h (IC_50_, μM).

Compounds	Aml-12
16	10.09 ± 0.58
IBC	17.65 ± 1.20
Paclitaxel	2.51 ± 0.13

### Compound 16 inhibited cell viability against human lung cancer cells

**Figure 1.**
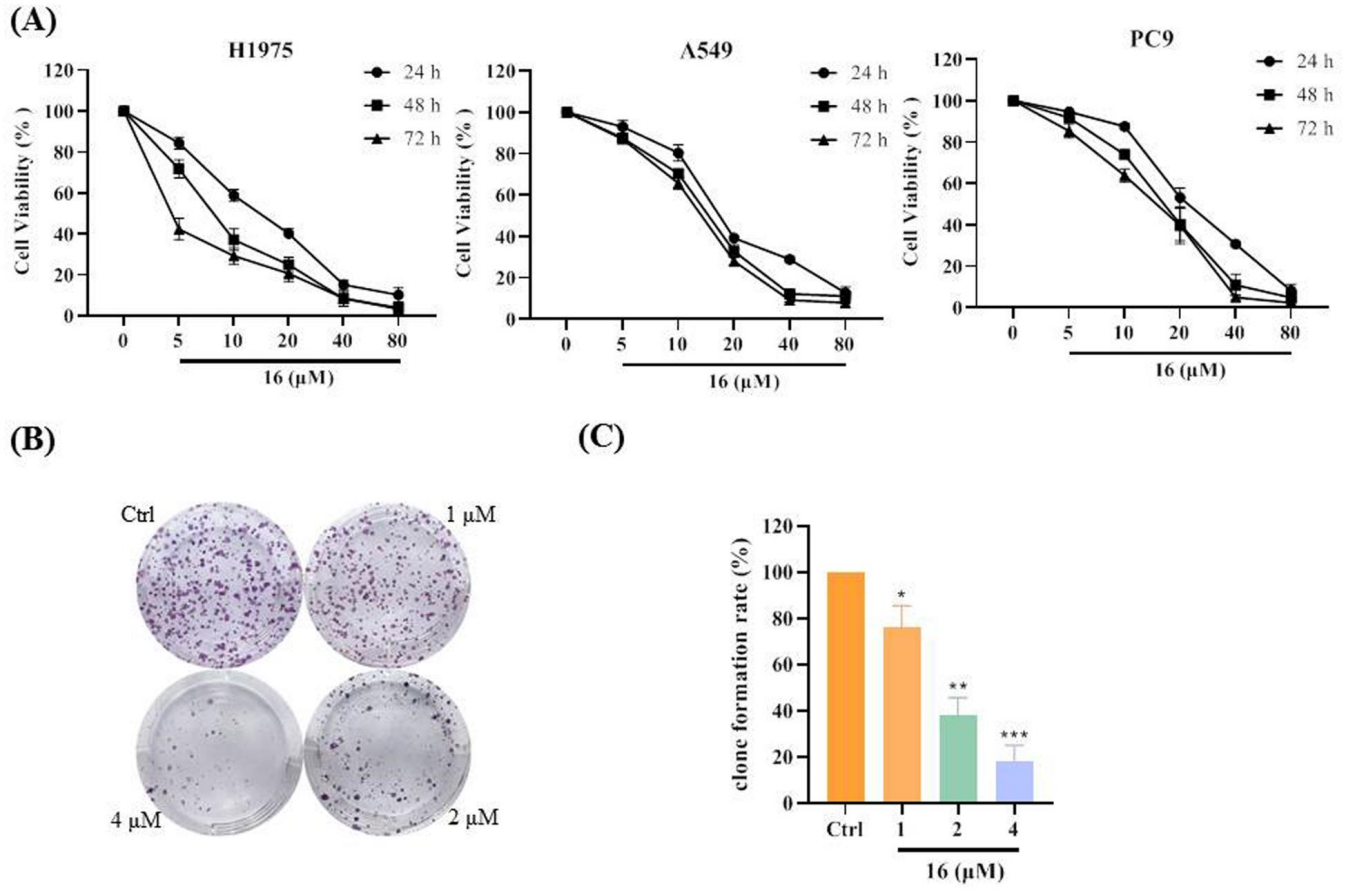
Inhibitory effect of compound **16** on cell viability in human lung cancer cells. (A) The cell viabilities of three human lung cancer (H1975, A549, and PC9) cells treated with various concentrations (0, 5, 10, 20, 40, and 80 μM) of **16** for 24, 48, and 72 h. (B) Effect of compound **16** on the ability of cells to form colonies in H1975 cells using a colony-formation assay. (C) Quantification of the colony-formation assay. *p < 0.05, **p < 0.01, and ***p < 0.001 compared with the control.

As shown in Figure 1(A), compound 16 inhibited cell viability against the three human lung cancer (H1975, A549, and PC9) cell lines. These results also indicated that H1975 cells were more sensitive to cell growth inhibition by compound 16. Similar to the MTT assays, the colony-formation assays showed that treatment with compound 16 (0, 1, 2, and 4 μM) inhibited cell growth in a concentration-dependent manner, with the colony formation rates of 100, 51.34, 27.70, and 14.87%, respectively (Figures 1(B,C)).

### Compound 16 induced apoptosis in H1975 cells

**Figure 2.**
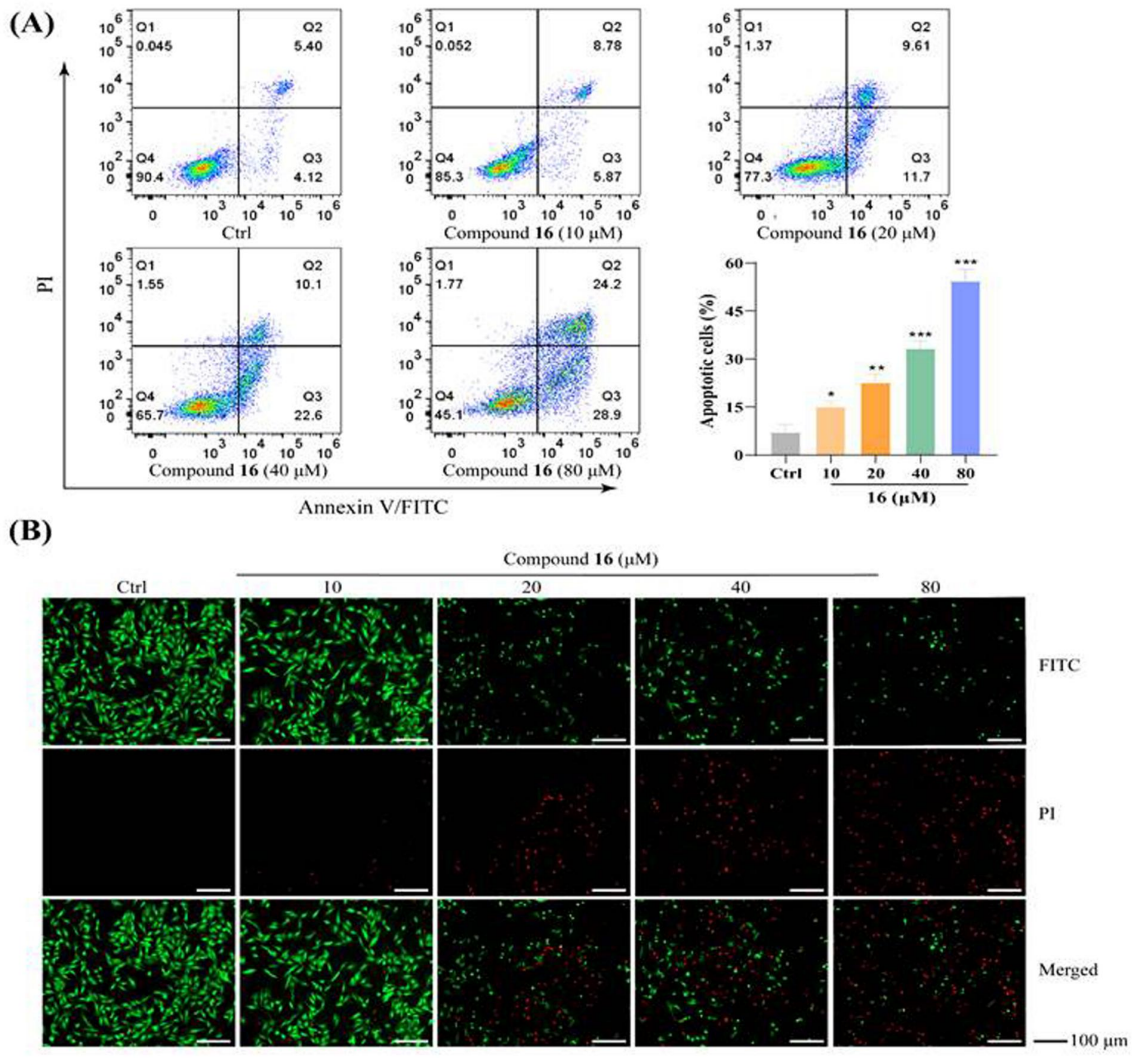
Compound **16** induced apoptosis in H1975 cells. (A) Flow cytometric analysis of cell death treated with different concentrations (10, 20, 40, and 80 μM) of **16** using Annexin V/PI dual staining. (B) H1975 cells were treated with **16** for 24 h, subjected to FITC and PI staining, and visualised using fluorescence microscopy. *p < 0.05, **p < 0.01, and ***p < 0.001 compared with the control.

Following inhibition with compound 16 for 24 h, flow cytometry was used to detect cell death. As shown in Figure 2(A), Annexin V/PI dual staining resulted in detected cell death rates of 14.7, 22.7, 34.3, and 54.9% for the H1975 cells exposed to increasing concentrations (10, 20, 40, and 80 μM) of compound 16, respectively. In addition, Calcein-AM and PI staining revealed significantly increased red fluorescence (death cells) in the H1975 cells by treated with the compound 16 (Figure 2(B)).

**Figure 3.**
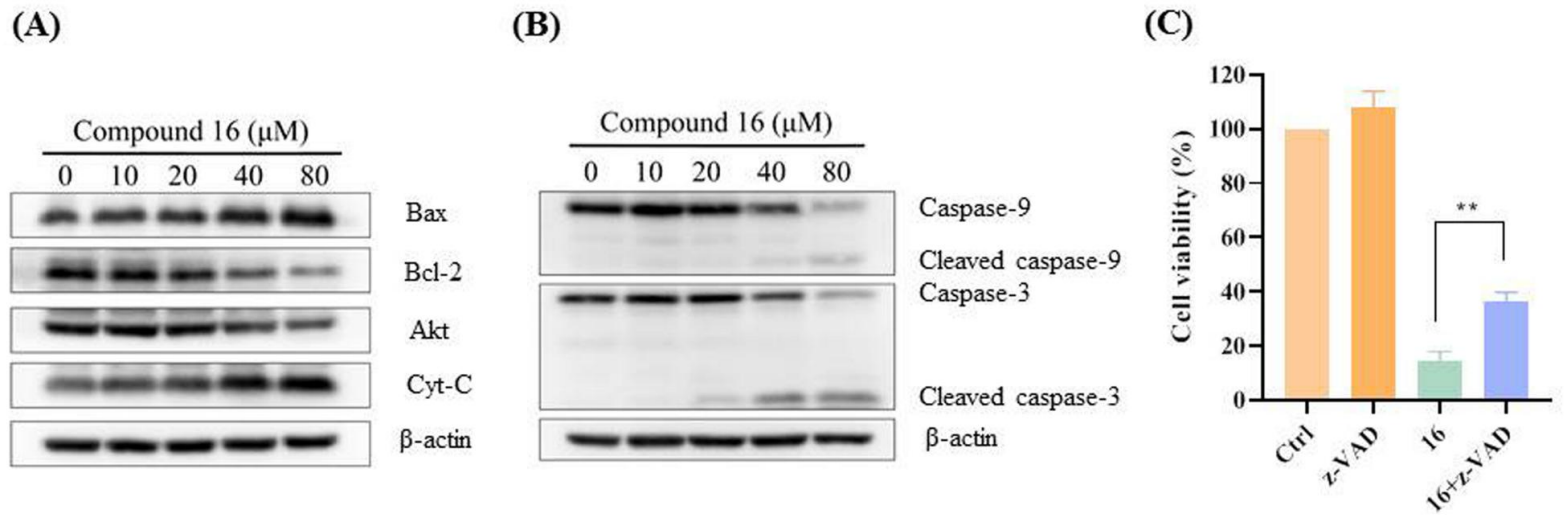
Compound **16** induced caspase-dependent apoptosis in H1975 cells. (A) Western blotting analysis of Bax, Bcl-2, Akt, and Cyt-C protein levels. (B) Western blotting analysis of caspase-9, and -3. (C) Cell viability following treated with compound **16** (40 μM) for 24 h alone or with pre-treatment with z-VAD (20 μM) as measured by MTT assay. **p < 0.01 compared with the control.

Western blotting showed that compound 16 induced apoptosis in H1975 cells as confirmed by the increasing the Bax and Cyt C expression, decreasing the Bcl-2 and Akt protein levels, and cleaving caspase-9 and -3 (Figures 3(A,B)). Furthermore, the H1975 cells were pre-treated with z-VAD-FMK, a pan-caspase inhibitor. As shown in Figure 3(C), we observed that the cell viability was 12.71% by treated with compound 16 alone and increased to 35.43% in combination with z-VAD-FMK (p < 0.01). These results indicated that compound 16 induced apoptosis in H1975 cells.

### Compound 16 induced necroptosis in H1975 cells

**Figure 4.**
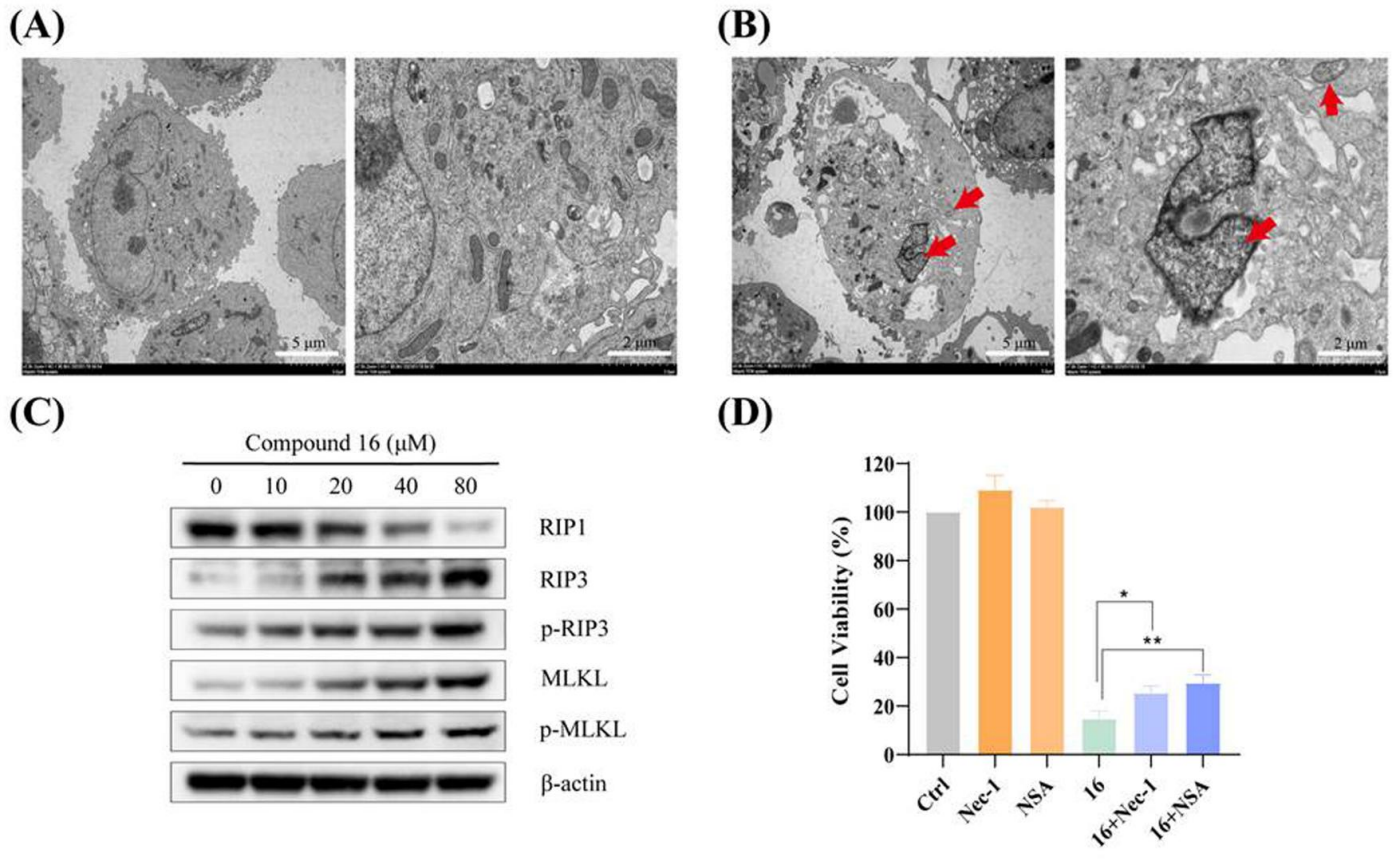
Compound **16** induced necroptosis in H1975 cells. (A) Morphological characteristics of the control using electron microscopy. (B) Morphological characteristics of **16**-treated H1975 cells showing necrosis. Red arrowheads indicated cell nuclear chromatin condensation, massive mitochondrial damage, and disruption of plasma membrane. (C) Western blotting analysis of RIP1, RIP3, p-RIP3, MLKL, and p-MLKL protein levels. (D) Cell viability following treated with compound **16** (40 μM) for 24 h alone or with pre-treatment with Nec-1 (20 μM) and NSA (20 μM) as measured by MTT assay. *p < 0.05 and **p < 0.01 compared with the control.

We observed morphological features of typical necrosis of H1975 cells under electron microscopy after treated with 40 μM of compound 16 (Figures 4(A,B)). Compound 16 induced typical nuclear chromatin condensation, massive mitochondrial damage, and disruption of plasma membrane. Western blotting analysis showed that compound 16 increased the expression of RIP3, p-RIP3, MLKL, and p-MLKL protein levels (Figure 4(C)). Furthermore, the H1975 cells were pre-treated with Nec-1 (necroptosis inhibitor). As shown in Figure 4(D), we observed that the cell viability was 14.82% by treated with compound 16 alone and increased to 25.33% in combination with Nec-1 (p < 0.05). Meanwhile, NSA (MLKL inhibitor) also had a protective effect on the compound 16 induced necroptosis in H1975 cells (p < 0.01). These results indicated that compound 16 induced necroptosis in H1975 cells via the regulation of the RIP3-MLKL signalling pathway.

### Effect of RIP3 knock-down on necroptosis of H1975 cells by siRNA

**Figure 5.**
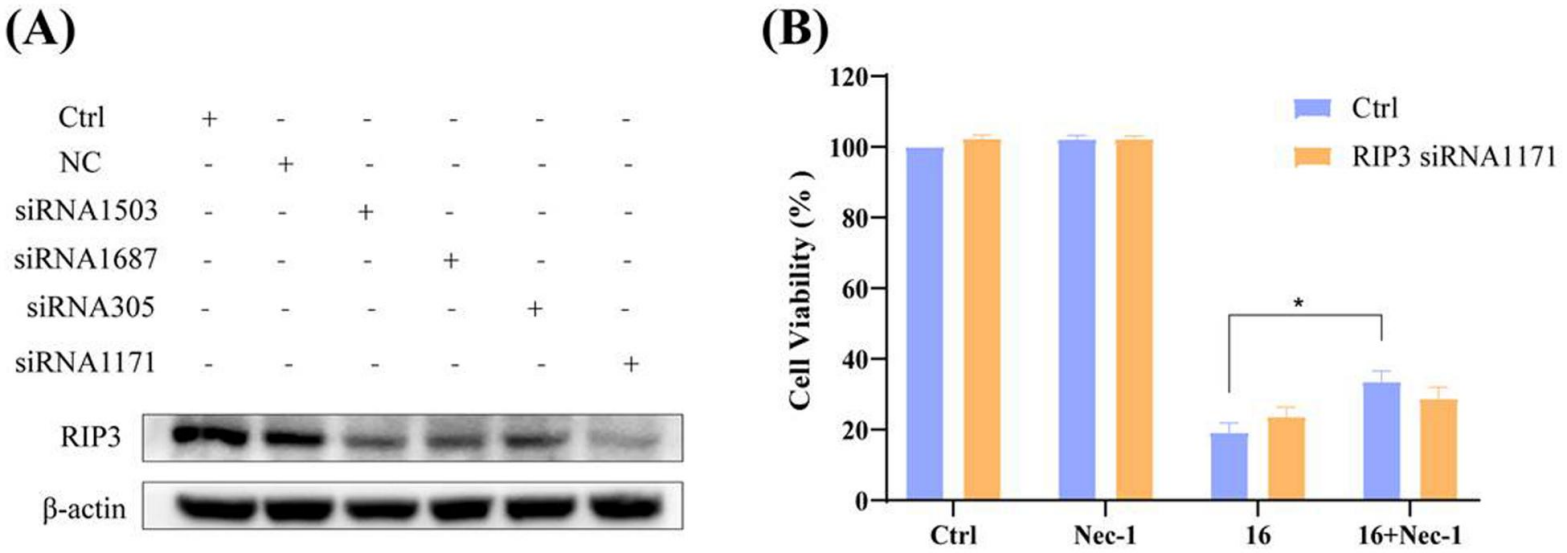
Knock-down of RIP3 using siRNA protected against compound **16** induced cell death in H1975 cells. (A) The H1975 cells were transfected with RIP3 siRNA, and whole-cell lysates were subjected to western blot analysis. (B) After transfection with RIP3 siRNA1171, the cells were treated with compound **16** (40 μM) for 24 h, alone or with pre-treatment with Nec-1. The cell viability was measured by MTT assay. *p < 0.05 compared between the groups of compound **16** and **16** with Nec-1.

To explore the role of RIP3 in compound **16**-induced necroptosis, we investigated the effect of RIP3 knock-down in H1975 cell viability using siRNA. As shown in Figure 5, the siRNA1171 gradually silenced RIP3 expression, leading to a loss of Nec-1 protective effect in the compound **16** treated cells. These results suggested that compound 16 induced necroptosis in H1975 cells, which may be mimicked by up-regulation of RIP3.

### Effect of compound 16 on mitochondrial function in H1975 cells

**Figure 6.**
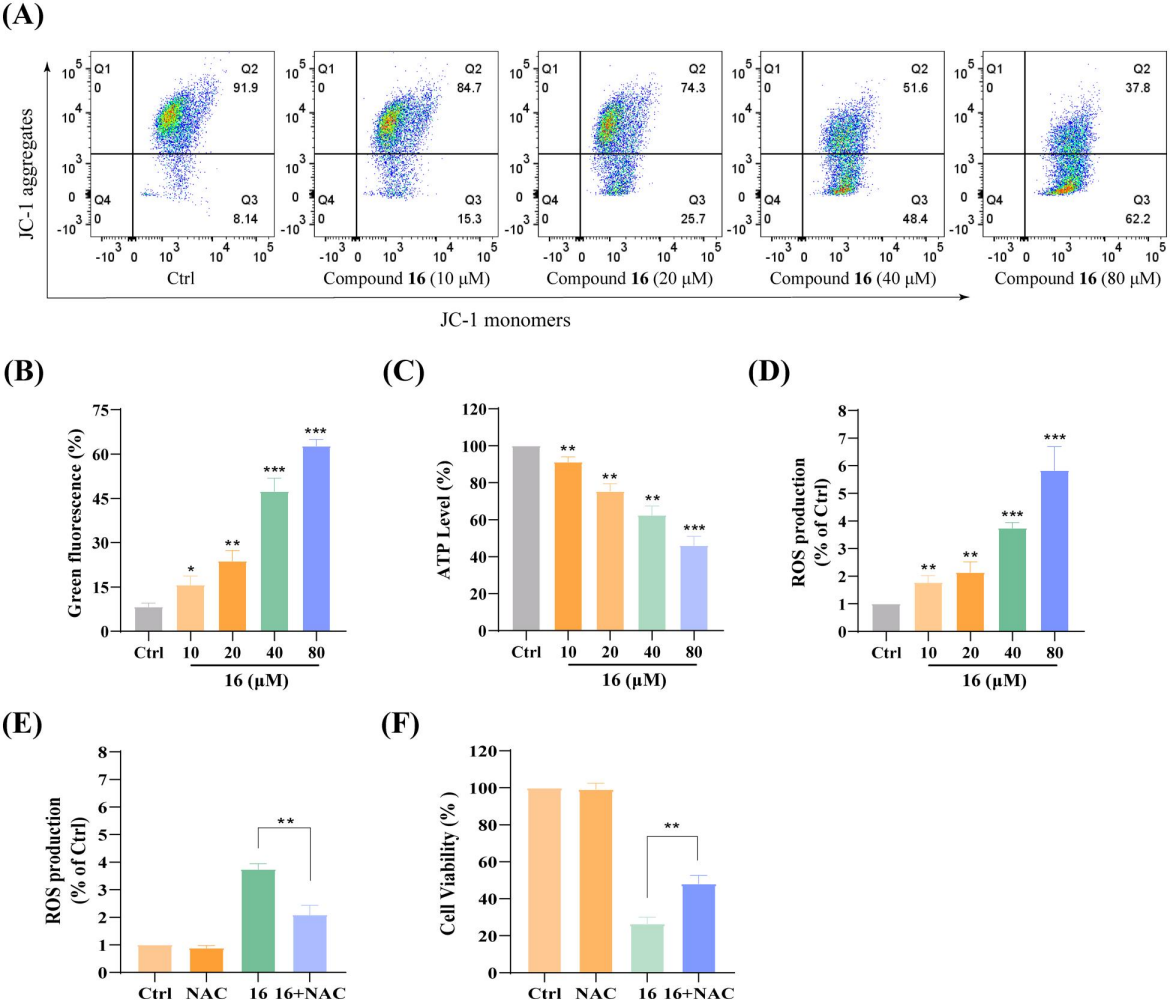
Effect of compound **16** on mitochondrial function in H1975 cells. (A) Mitochondrial membrane potential was assessed by JC-1 staining and flow cytometry. (B) Quantification of the green fluorescence. (C) Cellular ATP levels after treated with different concentrations (10, 20, 40, and 80 μM) of **16** for 6 h. (D) Intracellular ROS levels after treated with various concentrations of **16** for 6 h. (E) Intracellular ROS level analysis following treated with **16** with or pre-treatment with NAC (20 μM). (F) Cell viability following treated with **16** (40 μM) for 24 h alone or with pre-treatment with NAC as measured by MTT assay. *p < 0.05, **p < 0.01, and ***p < 0.001 compared with the control.

To explore whether compound 16 induced apoptosis and necroptosis are associated with mitochondrial function, we performed a flow cytometry with using the calcium indicator JC-1. As shown in Figures 6(A,B), H1975 cells were treated with various concentrations (10, 20, 40, and 80 μM) of compound 16, increasing green fluorescence of JC-1 from 8.14 to 61.20% was observed (p < 0.05). Next, we observed that cellular ATP levels were gradually decreased with different concentrations of compound 16 (Figure 6(C)). Furthermore, intracellular ROS levels gradually significantly enhanced by 1.86, 2.21, 3.74, and 5.82% when treatment with various concentrations (10, 20, 40, and 80 μM) of compound 16, respectively (Figure 6(D)).

To further illuminate the relationship between ROS and cell death, the NAC (as ROS scavenger) was applied to analyse the intracellular ROS level. As shown in Figure 6(E), we observed that the intracellular ROS level enhanced by 3.75% by treated with compound 16 (40 μM) alone, and the increasement decreased to 2.02% in combination with NAC (p < 0.01). In addition, NAC also had a protective effect on the compound 16 induced cell death in H1975 cells (Figure 6(F)) (p < 0.01). These results indicated that compound 16 induced various forms of H1975 cell death that are associated with mitochondrial function.

### Molecular docking of compound 16 on RIP3

**Figure 7.**
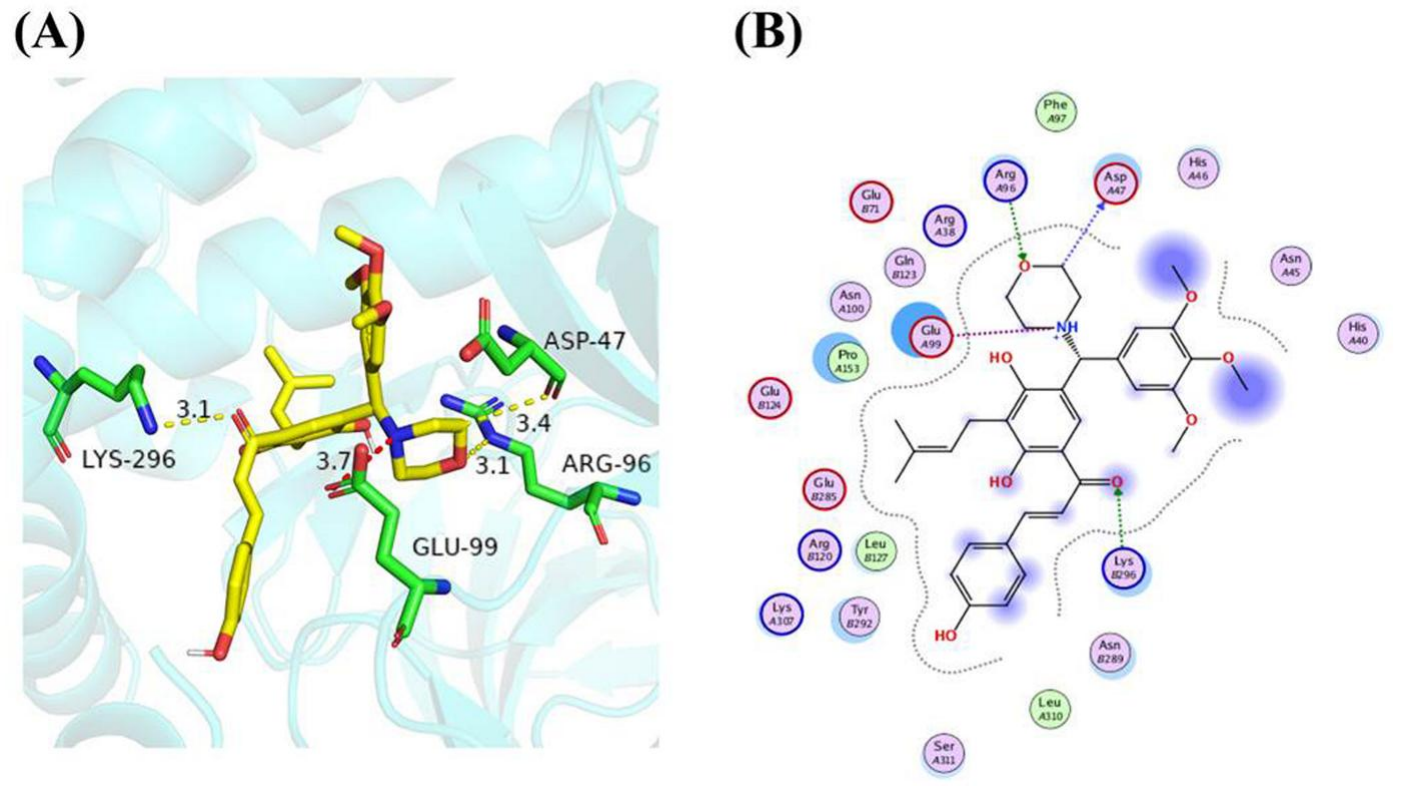
Molecular docking model of compound **16** on RIP3 protein. (A) 3D-binding model of compound **16** with RIP3 according to molecular docking analysis by MOE. (B) 2D-docking model of compound **16** with RIP3.

In the present study, the binding mode of compound **16** on RIP3 was revealed based on molecular docking. As shown in Figure 7, α,β-unsaturated carbonyl moiety formed a hydrogen bond with the Lys296 residue. Moreover, electrovalent bond and hydrogen bond were formed between the morpholine ring of compound **16** to occupy the Glu99 and Arg96 residue of RIP3, respectively.

## Discussion

Important advancements in anti-NSCLC drugs research and development have been achieved over the past 20 years. The use of small molecule tyrosine kinase inhibitors (e.g. gefitinib and erlotinib) and immunotherapy has resulted in unprecedented survival benefits for selected NSCLC patients^27–29^. However, the overall cure and survival rates for NSCLC remain low, especially in metastatic NSCLC patients^30^. In this study, we found that a novel IBC derivative 16 induced apoptosis and necroptosis in H1975 cells.

IBC, a natural bioactive chalcone, was found in the seed of a traditional Chinese medicine P. corylifolia L. Several studies revealed that IBC could be served as a potential lead compound for anti-cancer agent discovery and development^15^^,^^31–33^. Therefore, to find more effective anti-lung cancer IBC derivatives, three series of new IBC derivatives modified on phenolic hydroxyls (2-OH and 4′-OH) or benzene ring (C-5) position were synthesised. Interestingly, our results revealed that the aldehyde group, aminoguanidine, and Mannich base substituted on C-5 position of IBC could be enhanced the cytotoxic activity in H1975 cells. Among these, compound 16 had the most potent cytotoxic activity against H1975 cells, with IC50 values of 4.35 μM. Relative to the strong cytotoxicity activity against H1975 cells, compound 16 exhibited relatively low toxicity in normal Aml-12 cells, with an SI value of 2.32.

Apoptosis is one of the main cell death in which chemo-therapeutic agents kill cancer cells^13^^,^^34^. Our results showed that apoptosis was induced in human non-small cell lung cancer H1975 cells treated with compound 16 via regulating apoptosis-related proteins, including Bax, Bcl-2, Akt, and Cyt C. Many previous studies have reported that anti-cancer agents induced apoptosis via the activation of caspases, including caspase-3, -8, and -9^35^^,^^36^. Consistent with previous studies, we found that the treatment of compound 16 involves the increased in the Bax/Bcl-2 ratio, up-regulated Cyt C level, and cleaved of the caspase-9 and -3 with the induction of apoptosis. Thus, our results suggest that compound 16 induced apoptosis in H1975 cells in the caspase-dependent manner.

Necroptosis has been emerged as a potential therapeutic target for treating cancer^11^^,^^37^. In our previous studies, we found that IBC trigger various forms of death in human breast cancer MDA-MB-231 cells, including necroptosis^15^. Here, we showed for the first time that compound 16 induced apoptosis and necroptosis in H1975 cells, whereas necroptosis might be more important for compound 16 triggered PCD. The state of RIP1 and RIP3 determines whether they function as biomarkers that trigger or inhibit PCD^38^^,^^39^. In the cell viability experiments, Nec-1 or NSA significantly suppressed the cell death induced by compound 16 (40 μM) in the H1975 cells. These data showed that compound 16 increased RIP3, p-RIP3, MLKL, and p-MLKL levels in a concentration-dependent manner. In the siRNA experiments, the siRNA1171 inhibited compound **16** induced cell death and caused loss of the protective effect of Nec-1. Here, we also used compound **16** for molecular docking with RIP3 to indirectly reflect the interaction of **16** with the target protein. The binding energy of compound **16** with RIP3 was −13.4 kcal/mol. The strong binding affinity of **16** with RIP3 supports the role of compound **16** as a new RIP3-dependent necroptosis inducer.

In addition, the down-regulation of RIP1 protein levels was observed after treated with compound 16 (20, 40, and 80 µM). RIP1 is an important upstream signalling protein in the cell death receptor that regulates both cell survival and PCD^40^. RIP1 not only activates NF-κB against apoptosis but also induces death receptor-mediated apoptosis and necroptosis^41^^,^^42^. Corresponding to these references, our studies demonstrated that compound 16 not only induces necroptosis but also induces apoptosis in H1975 cells by treatment with 40 µM of compound 16. Therefore, the down-regulation of RIP1 expression can also be considered reasonable, and the molecular mechanism needs to be further studies.

Mitochondria is an important organelle that plays a central role in cellular ROS generation and energy production^43^^,^^44^. In this study, compound 16 induced PCD was closely associated with similar changes in mitochondrial function, mainly a decline in mitochondrial membrane potential, a decrease in ATP level, and the generation and accumulation of ROS. High levels of intracellular ROS have been recognised as the driving forces, leading to PCD^45^. When the H1975 cells were treated with NAC and compound 16, NAC significantly suppressed the increase of ROS level and cell death induced by compound 16. Therefore, our data suggest that compound 16 induced necroptosis in H1975 cells via the up-regulation of the RIP3-MLKL-ROS signalling pathway.

## Conclusions

In summary, we synthesised seventeen IBC derivatives and investigated their *in vitro* anti-cancer activities. Among these derivatives, compound **16** exhibited the most potent cytotoxic activity against H1975 cells. Our further study showed that compound 16 induced PCD in H1975 cells by triggering apoptosis and necroptosis. Importantly, compound 16 induced necroptosis via up-regulating RIP3-MLKL signalling pathway and ROS accumulation. Therefore, compound 16 may be a potential anti-NSCLC agent that warrants further investigation.

## Experimental section

### General information

All reagents used in the synthesis were obtained commercially and used without further purification unless otherwise specified. The reaction was monitored by thin-layer chromatography (TLC) on silica gel plates and visualised using a combination of UV. Flash column chromatography was performed using silica gel (300–400 mesh) was purchased from Qingdao Haiyang Chemical Co. Ltd. (Qingdao, China). The 1H-NMR and 13C-NMR spectra were recorded using TMS as the internal standard on a Bruker Avance (Bruker, Billerica, MA, USA) instrument. Chemical shifts were reported in ppm (d). High-resolution mass spectrometry (HRMS) spectra were determined using a Thermo Scientific LTQ Orbitrap XL mass spectrometer (Bruker, Bremerhaven, Germany). IBC was purchased from Mansite Biological Co. Ltd. (Chengdu, China). Human lung cancer (H1795, A549, and PC9) cells and normal mouse hepatocytes Aml-12 cells were purchased from the Shanghai Cell Bank of the Chinese Academy of Sciences (Shanghai, China). RPMI-1640 was purchased from Gibco (California, USA). Foetal bovine serum (FBS) was purchased from Sijiqing (Hangzhou, China). RIPA lysis buffer and dimethylsulphoxide (DMSO), Nec-1, z-VAD-FMK, NSA, and NAC were purchased from Sigma-Aldrich (St. Louis, MO, USA). BCA protein assay kit, Annexin V-FITC cell apoptosis detection kit, ATP assay kit, and ROS assay kit were purchased from Beyotime (Hangzhou, China). The JC-1 assay kit was purchased from BestBio (Shanghai, China). Anti-RIP1, anti-RIP3, anti-MLKL, anti-p-MLKL, and anti-caspase-3 antibodies were purchased from Abcam (Cambridge, MA, UK). Anti-Bax, anti-Bcl-2, anti-Akt, and β-Actin antibodies were purchased from Proteintech (Wuhan, China). Anti-Cyt-C, anti-caspase-3, and anti-p-RIP3 were purchased from Abclonal (Wuhan, China).

### General procedure for the preparation of compounds 1–17

#### The general procedure for the preparation of 1–3

To a solution of IBC (178 mg, 0.55 mmol) in DMF (6 ml) were added 10% Na2CO3 solution (3 ml) and CH3I (0.084 ml, 1.65 mmol), and the mixture was stirred at 65 °C for 10 h. The reaction was quenched with water and extracted three times with ethyl acetate. Furthermore, the combined extracts were dried over anhydrous Na2SO4 and concentrated under reduced pressure. Finally, the crude residue was purified by silica gel column chromatography using petroleum/ethyl acetate (10:1) to afford pure compounds 1–3, respectively.

##### (*E*)-1-(2,4-Dihydroxy-3-(3-methylbut-2-en-1-yl)phenyl)-3-(4-methoxyphenyl)prop-2-en-1-one (1)

Yellow solid, yield 9.7%; 1H-NMR (300 MHz, DMSO-d6) δ 13.96 (1H, s, -OH), 10.59 (1H, s, -OH), 8.08 (1H, d, J = 8.9 Hz, H-β), 7.88 (2H, d, J = 8.7 Hz, H-2′, H-6′), 7.80 (2H, d, J = 5.7 Hz, H-α), 7.04 (2H, d, J = 8.8 Hz, H-5′), 6.50 (1H, d, J = 8.9 Hz, H-5), 5.18 (m, H-2″), 3.83 (3H, s, H-7′), 3.25 (2H, d, J = 6.8 Hz, H-1″), 1.73 (3H, s, H-5″), 1.62 (3H, s, H-4″); 13C-NMR (75 MHz, DMSO-d6) δ 191.73, 163.56, 162.36, 161.43, 143.62, 130.96, 130.96, 130.47, 129.95, 129.94, 127.29, 122.33, 118.48, 114.43, 114.43, 112.70, 107.34, 55.42, 25.49, 21.27, 17.71; ESI-HRMS (m/z): calcd. for C21H23O4 + [M + H] + 339.1591, found 339.1592.

##### (*E*)-1-(2-Hydroxy-4-methoxy-3-(3-methylbut-2-en-1-yl)phenyl)-3-(4-hydroxyphenyl) prop-2-en-1-one (2)

Yellow solid, yield 22.0%; 1H-NMR (300 MHz, DMSO-d6) δ 13.81 (1H, s, -OH), 10.16 (1H, s, -OH), 8.25 (1H, d, J = 9.0 Hz, H-6), 7.81 (1H, d, J = 4.2 Hz, H-α), 7.80 (3H, d, J = 9.1 Hz, H-β, H-2′, H-6′), 6.86 (2H, d, J = 8.6 Hz, H-3′, H-5′), 6.69 (1H, d, J = 9.0 Hz, H-5), 5.13 (m, H-2″), 3.90 (3H, s, H-7), 3.27 (2H, d, J = 7.0 Hz, H-1″), 1.72 (3H, s, H-5″), 1.61 (3H, s, H-4″); 13C-NMR (75 MHz, DMSO-d6) δ 192.37, 162.91, 162.12, 160.41, 144.81, 131.41, 130.83, 130.43, 125.71, 122.04, 117.24, 115.89, 115.86, 114.04, 102.73, 56.06, 25.49, 21.25, 17.67; ESI-HRMS (m/z): calcd. for C21H23O4 + [M + H] + 339.1591, found 339.1591.

##### (*E*)-1-(2-Hydroxy-4-methoxy-3-(3-methylbut-2-en-1-yl)phenyl)-3-(4-methoxyphenyl) prop-2-en-1-one (3)

Yellow solid, yield 44.6%; 1H-NMR (300 MHz, DMSO-d6) δ 13.76 (1H, s, -OH), 8.28 (1H, d, J = 9.0 Hz, H-6), 7.94 (1H, d, J = 15.8 Hz, H-β), 7.92 (2H, d, J = 7.8 Hz, H-2′, H-6′), 7.85 (1H, d, J = 15.3 Hz, H-α), 7.50 (2H, d, J = 8.1 Hz, H-3′, H-5′), 6.70 (1H, d, J = 9.0 Hz, H-5), 5.13 (m, H-2″), 3.91 (3H, s, H-7′), 3.83 (3H, s, H-7), 3.28 (2H, d, J = 7.2 Hz, H-1″), 1.73 (3H, s, H-5″), 1.62 (3H, s, H-4″); 13C-NMR (75 MHz, DMSO-d6) δ 192.37, 163.00, 162.12, 161.57, 144.30, 131.15, 130.86, 130.58, 127.22, 122.00, 118.37, 115.89, 114.44, 114.02, 102.78, 56.08, 55.42, 25.48, 21.24, 17.67; ESI-HRMS (m/z): calcd. for C22H25O4 + [M + H] +353.1747, found 353.1749.

#### The general procedure for the preparation of 4–5

To a solution of IBC (149 mg, 0.46 mmol) in DMF (6 ml) were added Na2CO3 (36 mg, 0.34 mmol) and benzyl chloride (0.3 ml, 2.61 mmol), and the mixture was stirred at 65 °C for 12 h. The reaction mixture was quenched with water and extracted three times with ethyl acetate. Furthermore, the combined extracts were dried over anhydrous Na2SO4 and concentrated under reduced pressure. Finally, the crude residue was purified by silica gel column chromatography using petroleum/ethyl acetate = 10:1 to afford pure compounds 4 and 5, respectively.

##### (*E*)-1-(4-(Benzyloxy)-2-hydroxy-3-(3-methylbut-2-en-1-yl)phenyl)-3-(4-(benzyl-oxy) phenyl)prop-2-en-1-one (4)

Yellow solid, yield 32.9%; 1H-NMR (300 MHz, DMSO-d6) δ 13.77 (1H, s, -OH), 8.26 (1H, d, J = 9.2 Hz, H-6), 7.94 (1H, d, J = 15.2 Hz, H-β), 7.91 (2H, d, J = 8.7 Hz, H-2′, H-6′), 7.84 (1H, d, J = 15.3 Hz, H-α), 7.41 (m, J = 8.1 Hz, H-9, H-10, H-11, H-12, H-13, H-9′, H-10′, H-11′, H-12′, H-13′), 7.13 (2H, d, J = 8.8 Hz, H-3′, H-5′), 6.78 (1H, d, J = 9.1 Hz, H-5), 5.23 (m, H-2″), 5.19 (2H, s, H-7), 3.30 (2H, d, J = 8.9 Hz, H-1″), 1.63 (3H, s, H-5″), 1.61 (3H, s, H-4″); 13C-NMR (75 MHz, DMSO-d6) δ 192.37, 162.24, 162.06, 160.65, 144.26, 136.68, 131.16, 130.85, 130.46, 128.48, 127.97, 127.82, 127.54, 121.96, 118.51, 116.26, 115.26, 114.08, 104.04, 69.75, 69.43, 25.49, 21.40, 17.65; ESI-HRMS (m/z): calcd. for C34H32O4Na+ [M + Na] + 527.2198, found 527.2195.

##### (*E*)-3-(4-(Benzyloxy)phenyl)-1-(2,4-dihydroxy-3-(3-methylbut-2-en-1-yl)phenyl)prop-2-en-1-one (5)

Yellow solid, yield 13.6%; 1H-NMR (300 MHz, DMSO-d6) δ 13.83 (1H, s, -OH), 10.17 (1H, s, -OH), 8.23 (1H, d, J = 9.1 Hz, H-6), 7.86 (1H, d, J = 15.6 Hz, H-β), 7.80 (2H, d, J = 10.1 Hz, H-2′, H-6′), 7.75 (1H, d, J = 15.3 Hz, H-α), 7.41 (m, H-9′, H-10′, H-11′, H-12′, H-13′), 6.86 (2H, d, J = 8.5 Hz, H-3′, H-5′), 6.77 (1H, d, J = 9.1 Hz, H-5), 5.27 (2H, s, H-7′), 5.15 (m, H-2″), 3.32 (2H, d, J = 7.0 Hz, H-1″), 1.62 (3H, s, H-5″), 1.60(3H, s, H-4″); 13C-NMR (75 MHz, DMSO-d6) δ 192.37, 162.23, 161.95, 160.41, 144.83, 136.70, 131.40, 130.83, 130.28, 128.46, 127.94, 127.53, 125.72, 122.0, 117.26, 116.27, 115.86, 114.10, 103.97, 69.73, 25.50, 21.42, 17.65; ESI-HRMS (m/z): calcd. for C27H26O4Na+ [M + Na] + 437.1723, found 437.1725.

#### The procedure for the preparation of 6

To a solution of IBC (298 mg, 0.92 mmol) in CHCl3 (8 ml) was added 25% NaOH solution (4 ml) at 30 °C for 9 h. The reaction was quenched with water, and adjusted with 10% HCl to acidity. Then the mixture was extracted three times with ethyl acetate. The combined extracts were dried over anhydrous Na_2_SO_4_ and concentrated under reduced pressure. Finally, the crude residue was purified by silica gel column chromatography using CH_2_Cl_2_/MeOH = 30:1 to afford pure compound **6**.

##### (*E*)-2,4-Dihydroxy-5-(3-(4-hydroxyphenyl)acryloyl)-3-(3-methylbut-2-en-1-yl)benzylaldehyde (6)

Yellow solid, yield 4.1%; 1H-NMR (300 MHz, DMSO-d6) δ 14.61 (1H, s, -OH), 11.93 (1H, s, -OH), 10.30 (1H, s, H-7), 9.89 (2H, s, -OH), 8.85 (1H, s, H-6), 7.87 (2H, d, J = 16.6 Hz, H-α, H-β), 7.82 (2H, d, J = 8.3 Hz, H-2′, H-6′), 6.89 (2H, d, J = 8.3 Hz, H-3′, H-5′), 5.16 (m, H-2″), 3.28 (2H, d, J = 7.2 Hz, H-1″), 1.74 (3H, s, H-5″), 1.63 (3H, s, H-4″); 13C-NMR (75 MHz, DMSO-d6) δ 195.76, 192.37, 167.53, 163.84, 160.87, 146.17, 137.95, 131.74, 131.66, 125.50, 121.01, 116.46, 115.98, 115.47, 114.57, 113.73, 25.45, 20.57, 17.72; ESI-HRMS (m/z): calcd. for C21H21O5 +[M + H] + 353.1311, found 353.1383.

#### The procedure for the preparation of 7

To a solution of 6 (70 mg, 0.2 mmol) with aminoguanidine carbonate (27 mg, 0.2 mmol) in EtOH (3 ml) was added glacial acetic acid (0.5 ml), and the mixture was stirred at 50 °C for 5 h. The reaction mixture was extracted with ethyl acetate, washed with aqueous NaHCO3 and dried over anhydrous Na2SO4 and concentrated under reduced pressure. Finally, the crude residue was purified by silica gel column chromatography using CH2Cl2/MeOH = 20:1 to afford pure compound 7.

##### 2-((*E*)-2,4-Dihydroxy-5-((*E*)-3-(4-hydroxyphenyl)acryloyl)-3-(3-methylbut-2-en-1-yl) benzylidene)hydrazine-1-carboximidamide (7)

Yellow solid, yield 7.9%; 1H-NMR (300 MHz, DMSO-d6) δ 14.27 (1H, s, -OH), 10.39 (1H, s, -NH), 10.30 (1H, s, -OH), 8.51 (1H, s, -CH = N-), 8.37 (1H, s, H-6), 7.95 (1H, m, H-α), 7.89 (1H, m, H-β), 7.86 (3H, s, -NH2, =NH), 7.80 (2H, d, J = 8.6 Hz, H-2′, H-6′), 6.89 (2H, d, J = 8.5 Hz, H-3′, H-5′), 5.17 (m, H-2″), 3.29 (2H, d, J = 6.3 Hz, H-1″), 1.75 (3H, s, H-5″), 1.63 (3H, s, H-4″); 13C-NMR (75 MHz, DMSO-d6) δ 192.25, 164.48, 160.70, 160.31, 154.81, 148.54, 145.55, 131.91, 131.59, 131.34, 125.58, 121.67, 116.91, 115.94, 115.82, 113.56, 111.50, 25.48, 21.21, 17.81; ESI-HRMS (m/z): calcd. for C22H25N4O4 + [M + H] + 409.18758, found 409.18719.

#### The general procedure for the preparation of 8–17

To a solution of IBC (149 mg, 0.46 mmol) in MeCN (4 ml) was added aldehydes (4-pyridine-carboxaldehyde, 0.69 mmol; benzaldehyde, 0.69 mmol; 4-bromobenz-aldehyde, 0.69 mmol; 4-chlorobenzaldehyde, 0.69 mmol; 4-fluorobenzyl chloride, 0.69 mmol; 4-nitrobenzaldehyde, 0.69 mmol; 4-carboxy-benzaldehyde, 0.69 mmol; 4-methoxy-bezaldehyde, 0.69 mmol; 3,4,5-trimethoxybenzaldehyde, 0.69 mmol; 4-trifluoro-methylbenzaldehyde, 0.69 mmol) and morpholine (0.86 mmol). After stirred at 80 °C for 12 h, the reaction mixture was quenched with water and extracted three times with ethyl acetate. The combined extracts were dried over anhydrous Na2SO4 and concentrated under reduced pressure to provide the crude products. Finally, the crude residues were purified by silica gel column chromatography using petroleum/ethyl acetate = 3:1 to afford pure compounds 8–17, respectively.

##### (*E*)-1-(2,4-Dihydroxy-3-(3-methylbut-2-en-1-yl)-5-(morpholino(pyridin-4-yl)methyl) phenyl)-3-(4-hydroxyphenyl)prop-2-en-1-one (8)

Yellow solid, yield 66.2%; 1H-NMR (300 MHz, DMSO-d6) δ 13.93 (1H, s, -OH), 12.25 (1H, s, -OH), 10.25 (1H, s, -OH), 8.73 (2H, d, J = 5.6 Hz, H-4‴, H-6‴), 8.12 (1H, s, H-6), 7.87 (2H, d, J = 6.1 Hz, H-2′, H-6′), 7.80 (1H, d, J = 15.3 Hz, H-β), 7.77 (2H, d, J = 6.8 Hz, H-3‴, H-7‴), 7.72 (1H, d, J = 15.4 Hz, H-α), 6.90 (2H, d, J = 8.6 Hz, H-3′, H-5′), 5.15 (m, H-2″), 4.93 (1H, s, H-1‴), 3.75 (2H, m, H-9‴, H-10‴), 3.27 (2H, d, J = 6.7 Hz, H-1″), 2.54 (2H, t, J = 8.2 Hz, H-8‴, H-11‴), 1.71 (3H, s, H-5″), 1.61 (3H, s, H-4″); 13C-NMR (75 MHz, DMSO-d6) δ 191.67, 163.08, 160.57, 160.52, 147.13, 147.08, 144.77, 131.29, 131.03, 129.31, 125.06, 124.02, 121.95, 116.98, 115.91, 115.77, 112.99, 99.80, 65.63, 59.75, 51.79, 25.47, 21.37, 17.73; ESI-HRMS (m/z): calcd. for C30H32N2O5Na+ [M + Na] + 523.22034, found 523.22064.

##### (*E*)-1-(2,4-Dihydroxy-3-(3-methylbut-2-en-1-yl)-5-(morpholino(phenyl)methyl)phenyl)-3-(4-hydroxyphenyl)prop-2-en-1-one (9)

Yellow solid, yield 43.5%; 1H-NMR (300 MHz, DMSO-d6) δ 13.93 (1H, s, -OH), 10.20 (1H, s, -OH), 8.00 (1H, s, H-6), 7.78 (1H, d, J = 13.7 Hz, H-β), 7.76 (2H, d, J = 8.9 Hz, H-3‴, H-7‴), 7.71 (1H, d, J = 15.5 Hz, H-β), 7.54 (1H, d, J = 7.4 Hz, H-2′, H-6′), 7.36 (m, H-4‴, H-5‴, H-6‴), 6.89 (2H, d, J = 8.5 Hz, H-3′, H-5′), 5.21 (m, H-2″), 4.80 (1H, s, H-1‴), 3.70 (m, H-9‴, H-10‴), 3.30 (2H, d, J = 6.8 Hz, H-1″), 2.57 (2H, t, J = 11.0 Hz, H-8‴, H-11‴), 1.74 (3H, s, H-5″), 1.63 (3H, s, H-4″); 13C-NMR (75 MHz, DMSO-d6) δ 191.55, 162.68, 161.20, 160.38, 144.44, 131.26, 130.85, 129.03, 128.95, 128.12, 127.96, 125.66, 122.16, 117.33, 117.08, 115.86, 115.42, 112.60, 65.72, 59.75, 51.84, 25.50, 21.36, 17.75; ESI-HRMS (m/z): calcd. for C31H33NO5Na+ [M + Na] + 522.22509, found 522.22534.

##### (*E*)-1-(5-((4-Bromophenyl)(morpholino)methyl)-2,4-dihydroxy-3-(3-methylbut-2-en-1-yl)phenyl)-3-(4-hydroxyphenyl)prop-2-en-1-one (10)

Yellow solid, yield 16.0% 1H-NMR (300 MHz, DMSO-d6) δ 13.91 (1H, s, -OH), 10.20 (1H, s, -OH), 7.96 (1H, s, H-6), 7.72 (m, H-α, H-β, H-4‴, H-6‴), 7.64 (1H, s, -OH), 7.59 (1H, d, J = 6.4 Hz, H-2′, H-6′), 7.47 (2H, d, J = 6.7 Hz, H-3‴, H-7‴), 6.87 (2H, d, J = 6.7 Hz, H-3′, H-5′), 5.20 (m, H-2″), 4.70 (1H, s, H-1‴), 3.67 (2H, t, J = 16.1 Hz, H-9‴, H-10‴), 3.27 (2H, d, J = 7.0 Hz, H-1″), 2.45 (2H, t, J = 11.5 Hz, H-8‴, H-11‴), 1.73 (3H, s, H-5″), 1.62 (3H, s, H-4″); 13C-NMR (75 MHz, DMSO-d6) δ 191.55, 162.70, 161.20, 160.39, 144.43, 139.14, 131.89, 131.25, 130.90, 130.22, 130.19, 129.11, 125.69, 122.16, 121.07, 117.10, 115.89, 115.50, 112.66, 73.46, 65.91, 48.63, 25.52, 21.37, 17.77; ESI-HRMS (m/z): calcd. for C31H33BrNO5 +[M + H] + 578.15366, found 578.15399.

##### (*E*)-1-(5-((4-Chlorophenyl)(morpholino)methyl)-2,4-dihydroxy-3-(3-methylbut-2-en-1-yl)phenyl)-3-(4-hydroxyphenyl)prop-2-en-1-one (11)

Yellow solid, yield 21.7%; 1H-NMR (300 MHz, DMSO-d6) δ 13.90 (1H, s, -OH), 13.08 (1H, s, -OH), 10.14 (1H, s, -OH), 7.95 (1H, s, H-6), 7.76 (1H, d, J = 14.6 Hz, H-α), 7.72 (2H, d, J = 8.7 Hz, H-2′, H-6′), 7.68 (1H, d, J = 15.5 Hz, H-β), 7.54 (2H, d, J = 8.5 Hz, H-4‴, H-6‴), 7.45 (2H, d, J = 8.4 Hz, H-3‴, H-7‴), 7.36 (m, H-5‴), 6.88 (2H, d, J = 8.5 Hz, H-3′, H-5′), 5.20 (m, H-2″), 4,73 (1H, s, H-1‴), 3.68 (2H, t, J = 15.1 Hz, H-9‴, H-10‴), 3.28 (2H, d, J = 6.4 Hz, H-1″), 2.45 (2H, t, J = 12.5 Hz, H-8‴, H-11‴), 1.73 (3H, s, H-5″), 1.62 (3H, s, H-4″); 13C-NMR (75 MHz, DMSO-d6) δ 191.53, 162.68, 161.14, 160.37, 144.41, 138.71, 132.47, 131.22, 130.85, 129.84, 128.93, 125.66, 123.84, 122.14, 117.11, 115.85, 115.46, 112.64, 73.40, 65.89, 51.83, 21.33, 17.74; ESI-HRMS (m/z): calcd. for C31H32ClNO5Na+ [M + Na] + 556.18612, found 556.18616.

##### (*E*)-1-(5-((4-Fluorophenyl)(morpholino)methyl)-2,4-dihydroxy-3-(3-methylbut-2-en-1-yl)phenyl)-3-(4-hydroxyphenyl)prop-2-en-1-one (12)

Yellow solid, yield 18.21%; 1H-NMR (300 MHz, DMSO-d6) δ 13.91 (1H, s, -OH), 13.19 (1H, s, -OH), 10.16 (1H, s, -OH), 7.96 (1H, d, J = 16.1 Hz, H-β), 7.75 (3H, d, J = 8.7 Hz, H-2′, H-4‴, H-6′), 7.71 (1H, s, H-6), 7.69 (1H, d, J = 15.5 Hz, H-α), 7.54 (m, H-3‴, H-7‴), 7.21 (m, H-6‴), 6.88 (m, H-3′, H-5′), 5.21 (m, H-2″), 4.73 (1H, s, H-1‴), 3.67 (2H, t, J = 11.0 Hz, H-9‴, H-10‴), 3.28 (2H, d, J = 7.1 Hz, H-1″), 2.44 (2H, t, J = 11.8 Hz, H-8‴, H-11‴), 1.73 (3H, s, H-5″), 1.63 (3H, s, H-4″); 13C-NMR (75 MHz, DMSO-d6) δ 191.53, 162.64, 161.22, 160.37, 156.66, 144.39, 139.43, 131.23, 130.84, 130.01, 129.08, 125.66, 122.16, 117.37, 115.86, 115.60, 115.44, 114.77, 112.60, 73.34, 65.89, 63.30, 25.50, 21.35, 17.74; ESI-HRMS (m/z): calcd. for C31H32FNO5Na+ [M + Na] + 540.2157, found 540.2159.

##### (*E*)-1-(2,4-Dihydroxy-3-(3-methylbut-2-en-1-yl)-5-(morpholino(4-nitrophenyl)methyl)phenyl)-3-(4-hydroxyphenyl)prop-2-en-1-one (13)

Yellow solid, yield 20.38%; 1H-NMR (300 MHz, DMSO-d6) δ 13.91 (1H, s, -OH), 10.19 (1H, s, -OH), 8.26 (2H, d, J = 8.7 Hz, H-4‴, H-6‴), 8.06 (1H, s, H-6), 7.84 (2H, d, J = 8.7 Hz, H-2′, H-6′), 7.78 (1H, d, J = 15.3 Hz, H-β), 7.76 (2H, d, J = 8.4 Hz, H-3‴, H-7‴), 7.71 (1H, d, J = 15.3 Hz, H-α), 6.89 (2H, d, J = 8.5 Hz, H-3′, H-5′), 5.17 (m, H-2″), 4.96 (1H, s, H-1‴), 3.74 (m, H-9‴, H-10‴), 3.28 (2H, d, J = 6.6 Hz, H-1″), 2.61 (m, H-8‴, H-11‴), 1.72 (3H, s, H-5″), 1.61 (3H, s, H-4″); 13C-NMR (75 MHz, DMSO-d6) δ 191.62, 162.91, 160.71, 160.46, 146.99, 144.62, 144.60, 131.26, 130.97, 129.30, 129.20, 125.63, 124.18, 122.02, 117.03, 116.22, 115.89, 115.71, 112.86, 72.88, 65.65, 51.86, 25.47, 21.35, 17.74; ESI-HRMS (m/z): calcd. for C31H32N2O7Na+ [M + Na] + 567.21017, found 567.21033.

##### (*E*)-4-((2,4-Dihydroxy-5-(3-(4-hydroxyphenyl)acryloyl)-3-(3-methylbut-2-en-1-yl)phenyl)(morpholino)methyl)benzoic acid (14)

Yellow solid, yield 24.52%; 1H-NMR (300 MHz, DMSO-d6) δ 14.01 (1H, s, -COOH), 13.92 (1H, s, -OH), 7.99 (1H, s, H-6), 7.94 (2H, d, J = 8.1 Hz, H-4‴, H-6‴), 7.72 (m, H-2′, H-6′, H-α, H-β), 7.64 (2H, d, J = 8.1 Hz, H-3‴, H-7‴), 6.88 (2H, d, J = 8.3 Hz, H-3′, H-5′), 5.21 (m, H-2″), 4.77 (1H, s, H-1‴), 3.69 (m, H-9‴, H-10‴), 3.27 (2H, d, J = 8.0 Hz, H-1″), 2.42 (m, H-8‴, H-11‴), 1.73 (3H, s, H-5″), 1.62 (3H, s, H-4″); 13C-NMR (75 MHz, DMSO-d6) δ 191.55, 167.74, 162.73, 161.20, 160.47, 144.46, 144.26, 131.28, 130.89, 129.98, 129.93, 129.20, 128.05, 125.66, 122.16, 117.10, 117.02, 115.89, 115.50, 112.68, 73.93, 65.92, 51.85, 25.51, 21.37; ESI-HRMS (m/z): calcd. for C31H32N2O7Na+ [M + Na] + 566.2149, found 566.2151.

##### (*E*)-1-(2,4-Dihydroxy-5-((4-methoxyphenyl)(morpholino)methyl)-3-(3-methylbut-2-en-1-yl)phenyl)-3-(4-hydroxyphenyl)prop-2-en-1-one (15)

Yellow solid, yield 26.23%; 1H-NMR (300 MHz, DMSO-d6) δ 13.91 (1H, s, -OH), 13.42 (1H, s, -OH), 10.15 (1H, s, -OH), 7.91 (1H, s, H-6), 7.75 (2H, d, J = 8.7 Hz, H-2′, H-6′), 7.72 (m, H-α, H-β), 7.40 (2H, d, J = 8.6 Hz, H-3‴, H-7‴), 6.94 (2H, d, J = 8.6 Hz, H-4‴, H-6‴), 6.87 (2H, d, J = 8.5 Hz, H-3′, H-5′), 5.22 (m, H-2″), 4.65 (1H, s, H-1‴), 3.71 (1H, s, H-12‴), 3.66 (m, H-9‴, H-10‴), 3.28 (2H, d, J = 6.5 Hz, H-1″), 2.43 (m, H-8‴, H-11‴), 1.74 (3H, s, H-5″), 1.63 (3H, s, H-4″); 13C-NMR (75 MHz, DMSO-d6) δ 191.49, 162.55, 161.55, 160.34, 158.80, 144.29, 131.49, 131.21, 130.77, 129.35, 129.04, 125.70, 122.26, 117.77, 117.13, 115.84, 115.30, 114.25, 112.49, 73.71, 65.95, 55.08, 51.78, 25.51, 21.36, 17.74; ESI-HRMS (m/z): calcd. for C32H35NO6Na+ [M + Na] + 529.2464, found 530.2537.

##### (*E*)-1-(2,4-Dihydroxy-3-(3-methylbut-2-en-1-yl)-5-(morpholino(3,4,5-trimethoxyphe-nyl)methyl)phenyl)-3-(4-hydroxyphenyl)prop-2-en-1-one (16)

Yellow solid, yield 12.03%; 1H-NMR (300 MHz, DMSO-d6) δ 13.94 (1H, s, -OH), 13.48 (1H, s, -OH), 10.16 (1H, s, -OH), 7.92 (1H, s, H-6), 7.77 (1H, d, J = 15.3 Hz, H-β), 7.74 (2H, d, J = 7.7 Hz, H-2′, H-6′), 7.69 (1H, d, J = 15.2 Hz, H-α), 6.86 (2H, d, J = 8.6 Hz, H-3‴, H-7‴), 6.84 (2H, d, J = 7.1 Hz, H-3′, H-5′), 5.24 (m, H-2″), 4.59 (1H, s, H-1‴), 4.11 (3H, s, H-12‴, H-13‴, H-14‴), 3.75 (m, H-9‴, H-10‴), 3.61 (m, H-1″), 3.33 (m, H-8‴, H-11‴), 1.74 (3H, s, H-5″), 1.60 (3H, s, H-4″); 13C-NMR (75 MHz, DMSO-d6) δ 191.49, 162.43, 161.73, 160.35, 152.95, 144.32, 136.95, 135.27, 131.19, 130.73, 129.09, 125.71, 122.17, 117.46, 117.15, 115.85, 115.48, 112.51, 74.63, 65.95, 59.91, 59.76, 55.73, 25.47, 21.32, 17.73; ESI-HRMS (m/z): calcd. for C34H39NO8Na+ [M + Na] + 612.25679, found 612.25696.

##### (*E*)-1-(2,4-Dihydroxy-3-(3-methylbut-2-en-1-yl)-5-(morpholino(4-(trifluoromethyl) phenyl)methyl)phenyl)-3-(4-hydroxyphenyl)prop-2-en-1-one (17)

Yellow solid, yield 12.38%; 1H-NMR (300 MHz, DMSO-d6) δ 13.91 (1H, s, -OH), 10.18 (1H, s, -OH), 7.99 (1H, s, H-6), 7.75 (m, H-2′, H-6′, H-α, H-β, H-3‴, H-4‴, H-6‴, H-7‴), 6.88 (2H, d, J = 8.5 Hz, H-3′, H-5′), 5.20 (m, H-2″), 4.82 (1H, s, H-1‴), 4.10 (m, H-9‴, H-10‴), 3.69 (m, H-8‴, H-11‴), 3.28 (2H, d, J = 6.9 Hz, H-1″), 1.72 (3H, s, H-5″), 1.62 (3H, s, H-4″); 13C-NMR (75 MHz, DMSO-d6) δ 191.54, 162.77, 161.07, 160.40, 144.44, 144.40, 131.22, 130.86, 129.19, 129.16, 129.14, 128.79, 125.93, 125.65, 122.10, 117.05, 116.77, 115.86, 115.56, 112.70, 73.53, 65.87, 51.84, 25.48, 21.35, 17.73; ESI-HRMS (m/z): calcd. for C32H32F3NO5Na+ [M + Na] +590.21248, found 590.21252.

### Cell culture

Four cell lines (H1975, A549, PC9, and Aml-12) were cultured in RPMI 1640 medium containing 10% foetal bovine serum (FBS) and 1% penicillin/streptomycin in an atmosphere with 5% CO2 and 95% relative humidity at 37 °C.

### MTT assay

Cell viability was examined using the MTT assay. The cells grown in the logarithmic phase were seeded in 96-well plates at a density of 5 × 103 per well. The cells were treated with various concentrations (0, 5, 10, 20, 40, and 80 μM) of the compound at different times, respectively. At the end of time point, MTT solution (5 mg/ml) was added to each well and incubated at 37 °C for 4 h. Next, the media was removed, and formazan was dissolved in DMSO. Finally, absorbance was measured with a microplate spectrophotometer at 490 nm (Synergy HT, BioTek, VT, USA).

### Colony-formation assay

Cells were seeded at a density of 1.5 × 103 cells/well into 6-well plates. When colonies formed, the media was replaced with fresh media at various concentrations of compound 16 (0, 1, 2, and 4 μM) and incubated for 10 days. At the end of the incubation time, the cells were washed three times with PBS, fixed with 4% paraformaldehyde for 15 min, stained with crystal violet solution for 10 min, and photographed.

### Flow cytometry with Annexin V/PI dual staining

Cells were seeded (3 × 105 per well) in 6-well plates and incubated overnight. After adherence, the cells were treated with various concentrations (0, 10, 20, 40, and 80 μM) of compound 16 for 24 h. Furthermore, the cells were harvested, washed three times with PBS, and stained with Annexin V (FITC Annexin V) and propidium iodide (PI) staining solution for 30 min in the dark. The percentage of cell death was measured by flow cytometry (Becton-Dickinson, Franklin Lakes, NJ, USA).

### Fluorescence staining

Cells were seeded at a density of 5 × 103 cells/well in 12-well plates and treated with different concentrations (0, 10, 20, 40, and 80 μM) of 16. After incubated for 24 h, the cells were stained with Calcein/PI solutions and subsequent incubated at 37 °C for 30 min. The fluorescent intensity of the cells was observed using a fluorescence microscope (Olympus, Japan).

### Western blotting

Cells were seeded in 100 mm dishes at a density of 3 × 106 cells/well and treated with different concentrations (0, 10, 20, 40, and 80 μM) of compound 16. After incubated for 24 h, the cells were harvested, and washed three times with PBS solution. Next, the cells were lysed with RIPA lysis buffer for 30 min on ice. Protein quantification was determined using the BCA protein assay kit. Equal amounts of proteins were separated by sodium dodecyl sulfate-polyacrylamide gel electrophoresis (SDS-PAGE) and transferred to PVDF membrane. The membrane was blocked with 5% non-fat milk and incubated with primary antibodies (Bax, Bcl-2, Akt, Cyt C, Caspase-9, Caspase-3, RIP1, RIP3, p-RIP3, MLKL, p-MLKL, and β-actin) for 2 h at room temperature, followed by the secondary antibodies for 2 h at room temperature. Finally, the blots were detected by a chemiluminescent gel imaging system (Bio-Rad, Hercules, CA, USA).

### Electron microscopy

Cells were seeded in a 6-well plate (1.5 × 103 cells/well) and treated with compound 16 (40 μM) and control for 6 h. At the end of time point, the cells were washed with PBS, fixed with 2.5% glutaraldehyde, and stored at 4 °C. Post-fixed samples were sent to Servicebio (Wuhan, China) for subsequent experiments using transmission electron microscopy (TEM).

### Knockdown of RIP3 using siRNA

RIP3 siRNAs were purchased from Gene-Pharma (Shanghai, China). The siRNAs were transiently transfected into H1975 cells using a lipofectamine 2000 reagent kit (Invitrogen, CA, USA) according to the manufacturer’s protocol. After 48 h of transfection, the cells were collected for western blot analysis. The sequences of siRNA used for experiments with human RIP3 were as follows: positive control sense, 5′-UGA CCU CAA CUA CAU GGU UTT-3′ and antisense, 5′-AAC CAU GUA GUU GAG GUC ATT-3′; negative control sense, 5′-UUC UCC GAA CGU GUC ACG UTT-3′ and antisense, 5′-ACG UGA CAC GUU CGG AGA ATT-3′; RIP3 homo-1687 sense, 5′-GCC ACA GGG UUG GUA UAA UTT-3′ and antisense, 5′-AUU AUA CCA ACC CUG UGG CTT-3′; RIP3 homo-305 sense, 5′-GCG GUC AAG AUC GUA AAC UTT-3′ and antisense, 5′-AGU UUA CGA UCU UGA CCG CTT-3′; RIP3 homo-1503 sense, 5′-GAC CGC CUC GUU AAC AUA ATT-3′ and antisense, 5′-UAU AUG UUA ACG AGC GGU CTT-3′; and RIP3 homo-1171 sense, 5′-CCA GCAC CUC UCG UAA UGA ATT-3′ and antisense, 5′-AUC AUU ACG AGA GUG CUG GTT-3′.

### Detection of mitochondrial membrane potential with JC-1

Cells were seeded in 6-well plates at a density of 3 × 105 cells/well and treated with various concentrations (0, 10, 20, 40, and 80 μM) of compound 16. After incubated for 24 h, the cells were harvested, stained with JC-1 solution, and incubated for 30 min at 37 °C. Finally, the cells were detected and analysed using a flow cytometry (Becton-Dickinson, Franklin Lakes, NJ, USA).

### ATP detection

Cells were seeded in 6-well plates at a density of 3 × 105 cells/well and incubated with different concentrations (0, 10, 20, 40, and 80 μM) of compound 16 for 6 h. Afterwards, the cells were washed three times with PBS, lysed with RIPA buffer, and the lysates were centrifuged at 12 000 rpm for 5 min. The intracellular ATP levels were measured using a luminoskan luminometer (Thermo Scientific, Atlanta, GA, USA), according to the ATP assay kit’s protocol.

### Detection of ROS

Cells were seeded in 6-well plates at a density of 3 × 105 cells/well and incubated with different concentrations (0, 10, 20, 40, and 80 μM) of compound 16 or pre-treated with NAC (20 μM) incubated for 6 h, the cells were co-incubated with DCFH-DA for 20 min at 37° C in the dark. Finally, the intracellular ROS levels were measured using flow cytometry (Becton-Dickinson, Franklin Lakes, NJ, USA).

### Molecular docking

The compound **16** bound to RIP3 (PDB code: 6oko) was used for molecular docking experiment using MOE software. PyMOL software was used for further visualisation, figure preparation, and conformational analyses.

### Statistical analysis

All data were analysed using GraphPad Prism software version 8.0. Student’s t-tests were performed to analyse the differences between the two groups. The results are presented as the mean ± SD from three independent experiments. *p < 0.05, **p < 0.01, and ***p < 0.001 were considered statistically significant.

## Supplementary Material

Supplemental Material
