# Supplementary material for: Novel isobavachalcone derivatives induce apoptosis and necroptosis in human non-small cell lung cancer H1975 cells
Source: J Enzyme Inhib Med Chem. 2023 Dec 12;39(1):2292006. doi: 10.1080/14756366.2023.2292006 (PMC11721617; doi:10.1080/14756366.2023.2292006)
Supplement: Supplemental Material [file IENZ_A_2292006_SM6136.pdf]

Supplementary materials for

Novel isobavachalcone derivatives induces apoptosis and necroptosis in human non-small cell lung cancer H1975 cells

Jie Chen[a,†], Long Zhao[a,b,†], Meng-Fan Xu[a], Di Huang[a], Xiao-Long Sun[a], Yu-Xin Zhang[b,c], Hong-Mei Li[a,b,*] and Cheng-Zhu Wu[a,b,*]

[a] School of Pharmacy, Bengbu Medical College, Bengbu 233030, Anhui, China.

[b] Anhui Province Biochemical Pharmaceutical Engineering Technology Research Center, Bengbu 233030, Anhui, China.

[c] School of Laboratory Medicine, Bengbu Medical College, 2600 Donghai Road, Bengbu 233030, Anhui, China

[*] Corresponding authors: Tel:+86-552-3171262, E-mail: lihongmei@bbmc.edu.cn (H.-M. Li); Tel:+86-552-3175232, E-mail: wuchengzhu0611@bbmc.edu.cn (C.-Z. Wu).

[†] These authors contributed equally to this work.

List of supplemental data

Figure S1 ^{1}H-NMR (300 MHz, DMSO-d_6) spectrum of compound 1 4
Figure S2 ^{13}C-NMR (75 MHz, DMSO-d_6) spectrum of compound 1 4
Figure S3 ESI-HRMS spectrum of compound 1 5
Figure S4 ^{1}H-NMR (300 MHz, DMSO-d_6) spectrum of compound 2 6
Figure S5 ^{13}C-NMR (75 MHz, DMSO-d_6) spectrum of compound 2 6
Figure S6 ESI-HRMS spectrum of compound 2 7
Figure S7 ^{1}H-NMR (300 MHz, DMSO-d_6) spectrum of compound 3 8
Figure S8 ^{13}C-NMR (75 MHz, DMSO-d_6) spectrum of compound 3 8
Figure S9 ESI-HRMS spectrum of compound 3 9
Figure S10 ^{1}H-NMR (300 MHz, DMSO-d_6) spectrum of compound 4 10
Figure S11 ^{13}C-NMR (75 MHz, DMSO-d_6) spectrum of compound 4 10
Figure S12 ESI-HRMS spectrum of compound 4 11
Figure S13 ^{1}H-NMR (300 MHz, DMSO-d_6) spectrum of compound 5 12
Figure S14 ^{13}C-NMR (75 MHz, DMSO-d_6) spectrum of compound 5 12
Figure S15 ESI-HRMS spectrum of compound 5 13
Figure S16 ^{1}H-NMR (300 MHz, DMSO-d_6) spectrum of compound 6 14
Figure S17 ^{13}C-NMR (75 MHz, DMSO-d_6) spectrum of compound 6 14
Figure S18 ESI-HRMS spectrum of compound 6 15
Figure S19 ^{1}H-NMR (300 MHz, DMSO-d_6) spectrum of compound 7 16
Figure S20 ^{13}C-NMR (75 MHz, DMSO-d_6) spectrum of compound 7 16
Figure S21 ESI-HRMS spectrum of compound 7 17
Figure S22 ^{1}H-NMR (300 MHz, DMSO-d_6) spectrum of compound 8 18
Figure S23 ^{13}C-NMR (75 MHz, DMSO-d_6) spectrum of compound 8 18
Figure S24 ESI-HRMS spectrum of compound 8 19
Figure S25 ^{1}H-NMR (300 MHz, DMSO-d_6) spectrum of compound 9 20
Figure S26 ^{13}C-NMR (75 MHz, DMSO-d_6) spectrum of compound 9 20
Figure S27 ESI-HRMS spectrum of compound 9 21
Figure S28 ^{1}H-NMR (300 MHz, DMSO-d_6) spectrum of compound 10 22
Figure S29 ^{13}C-NMR (75 MHz, DMSO-d_6) spectrum of compound 10 22
Figure S30 ESI-HRMS spectrum of compound 10 23
Figure S31 ^{1}H NMR (300 MHz, DMSO-d_6) spectrum of compound 11 24
Figure S32 ^{13}C NMR (75 MHz, DMSO-d_6) spectrum of compound 11 24
Figure S33 ESI-HRMS spectrum of compound 11 25
Figure S34 ^{1}H-NMR (300 MHz, DMSO-d_6) spectrum of compound 12 26
Figure S35 ^{13}C-NMR (75 MHz, DMSO-d_6) spectrum of compound 12 26
Figure S36 ESI-HRMS spectrum of compound 12 27
Figure S37 ^{1}H-NMR (300 MHz, DMSO-d_6) spectrum of compound 13 28
Figure S38 ^{13}C NMR (75 MHz, DMSO-d_6) spectrum of compound 13 28
Figure S39 ESI-HRMS spectrum of compound 13 29
Figure S40 ^{1}H-NMR (300 MHz, DMSO-d_6) spectrum of compound 14 30
Figure S41 ^{13}C-NMR (75 MHz, DMSO-d_6) spectrum of compound 14 30
Figure S42 ESI-HRMS spectrum of compound 14 31

Figure S43 ¹H-NMR (300 MHz, DMSO-d_6) spectrum of compound 15 .... **32**
Figure S44 ¹³C-NMR (75 MHz, DMSO-d_6) spectrum of compound 15 .... **32**
Figure S45 ESI-HRMS spectrum of compound 15 .... **33**
Figure S46 ¹H-NMR (300 MHz, DMSO-d_6) spectrum of compound 16 .... **34**
Figure S47 ¹³C-NMR (75 MHz, DMSO-d_6) spectrum of compound 16 .... **34**
Figure S48 ESI-HRMS spectrum of compound 16 .... **35**
Figure S49 ¹H-NMR (300 MHz, DMSO-d_6) spectrum of compound 17 .... **36**
Figure S50 ¹³C-NMR (75 MHz, DMSO-d_6) spectrum of compound 17 .... **36**
Figure S51 ESI-HRMS spectrum of compound 17 .... **37**

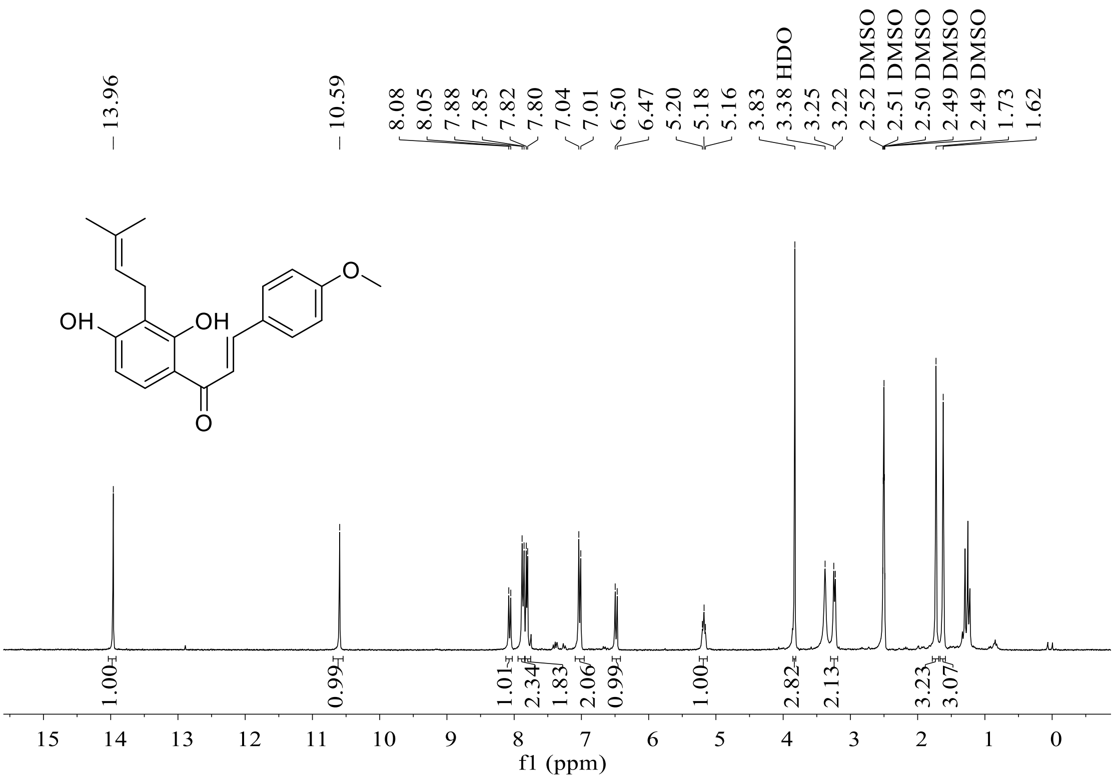

Figure S1 ^{1}H-NMR (300 MHz, DMSO-d_6) spectrum of compound 1

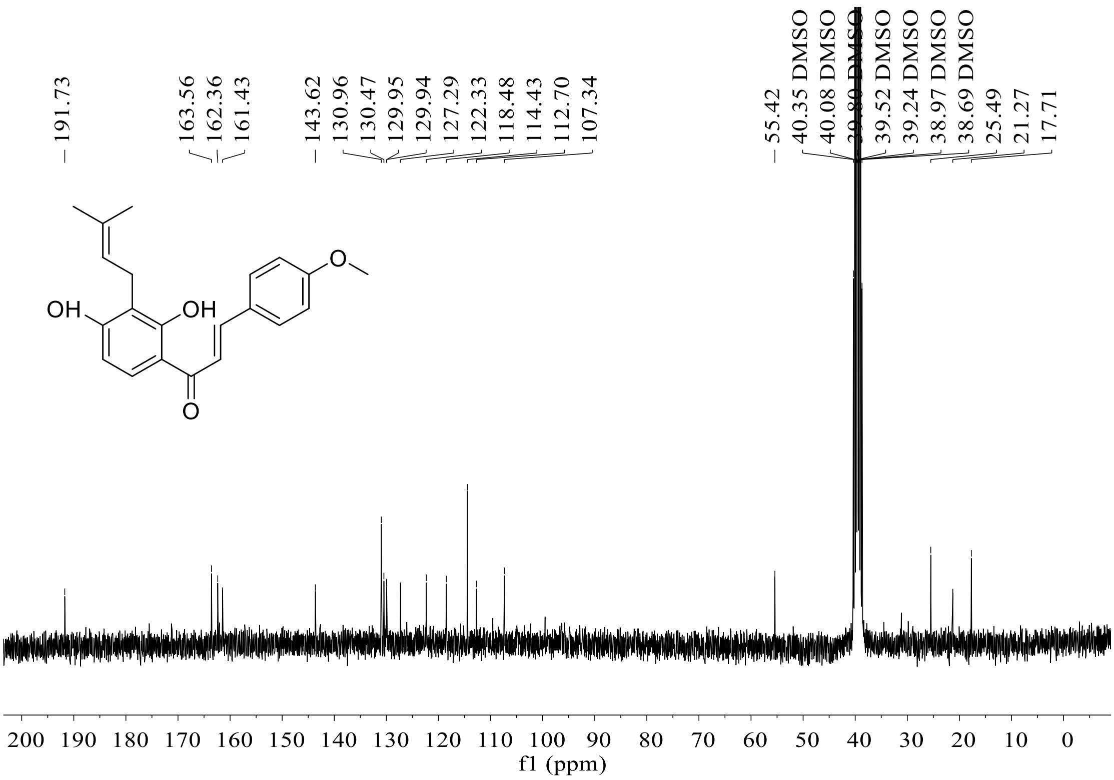

Figure S2 ^{13}C-NMR (75 MHz, DMSO-d_6) spectrum of compound 1

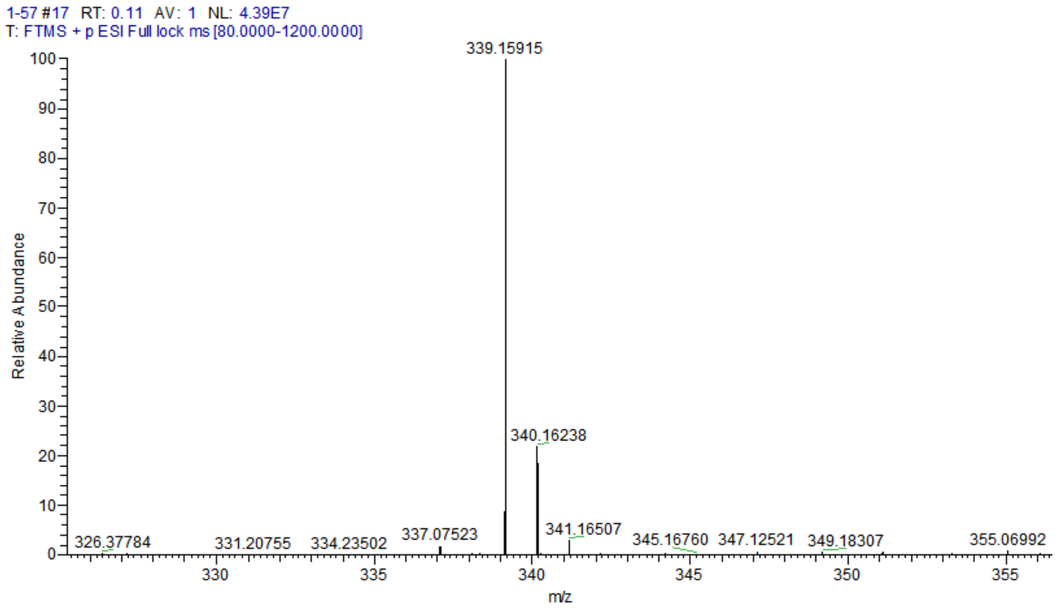

Figure S3 ESI-HRMS spectrum of compound 1

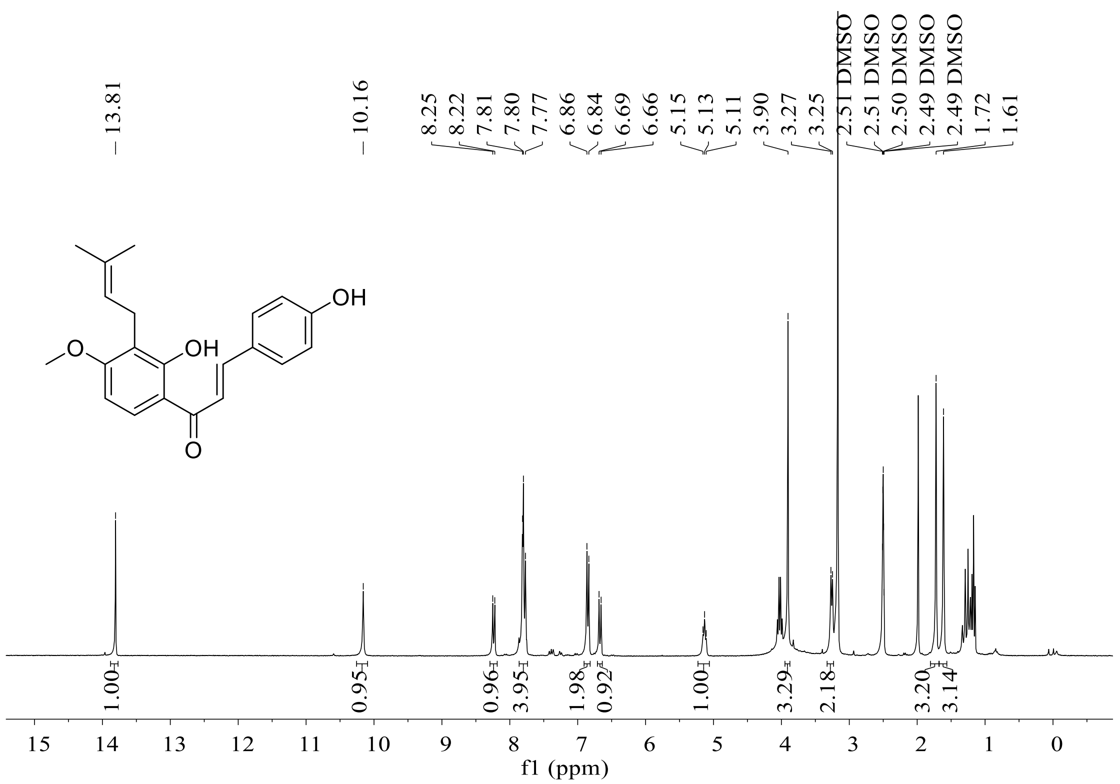

Figure S4 ^{1}H-NMR (300 MHz, DMSO-d_6) spectrum of compound 2

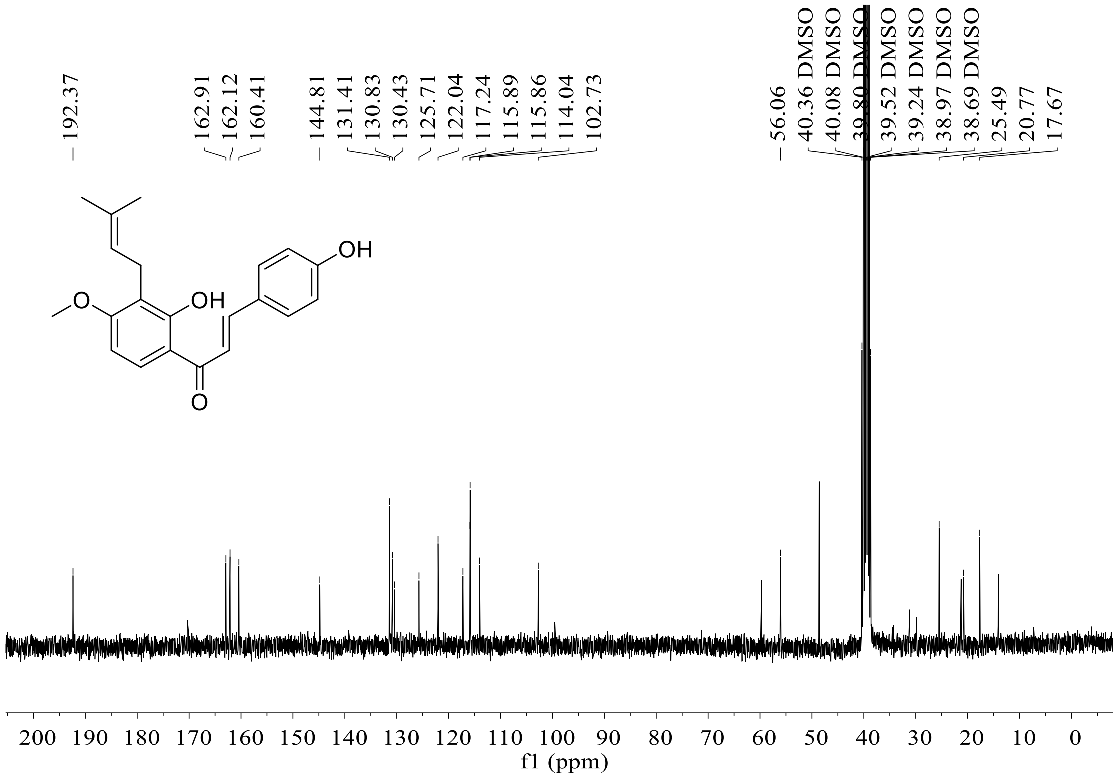

Figure S5 ^{13}C-NMR (75 MHz, DMSO-d_6) spectrum of compound 2

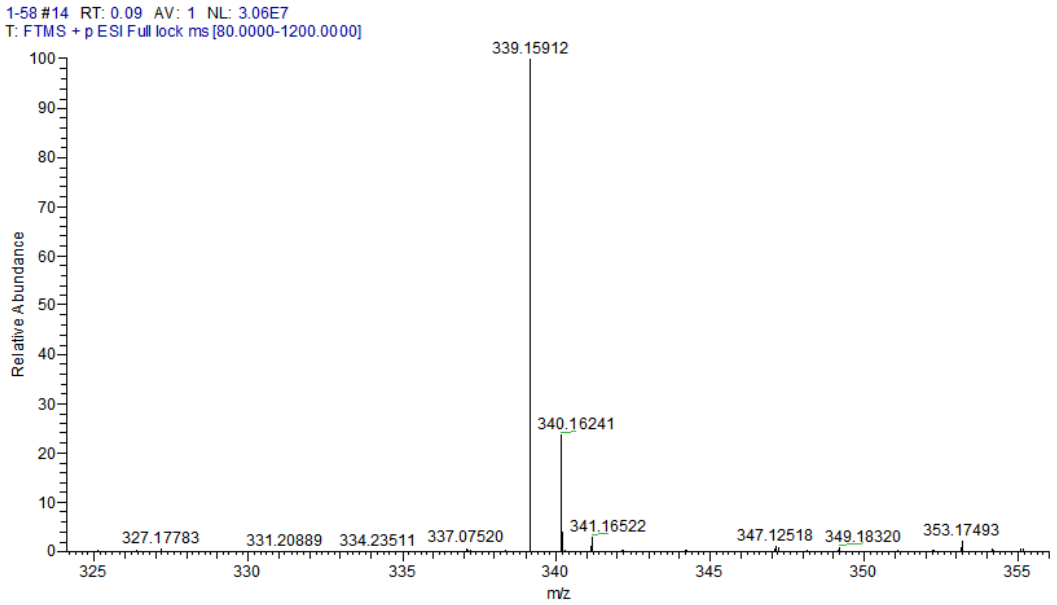

Figure S6 ESI-HRMS spectrum of compound 2

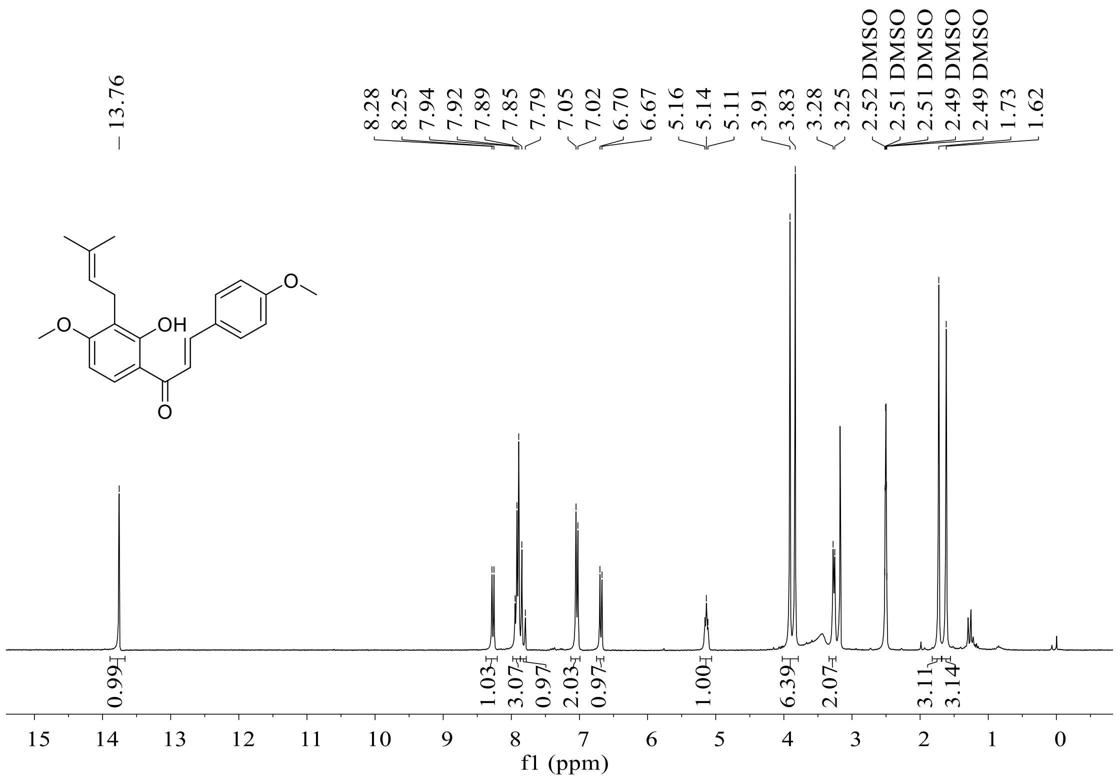

Figure S7 ^{1}H-NMR (300 MHz, DMSO-d_6) spectrum of compound 3

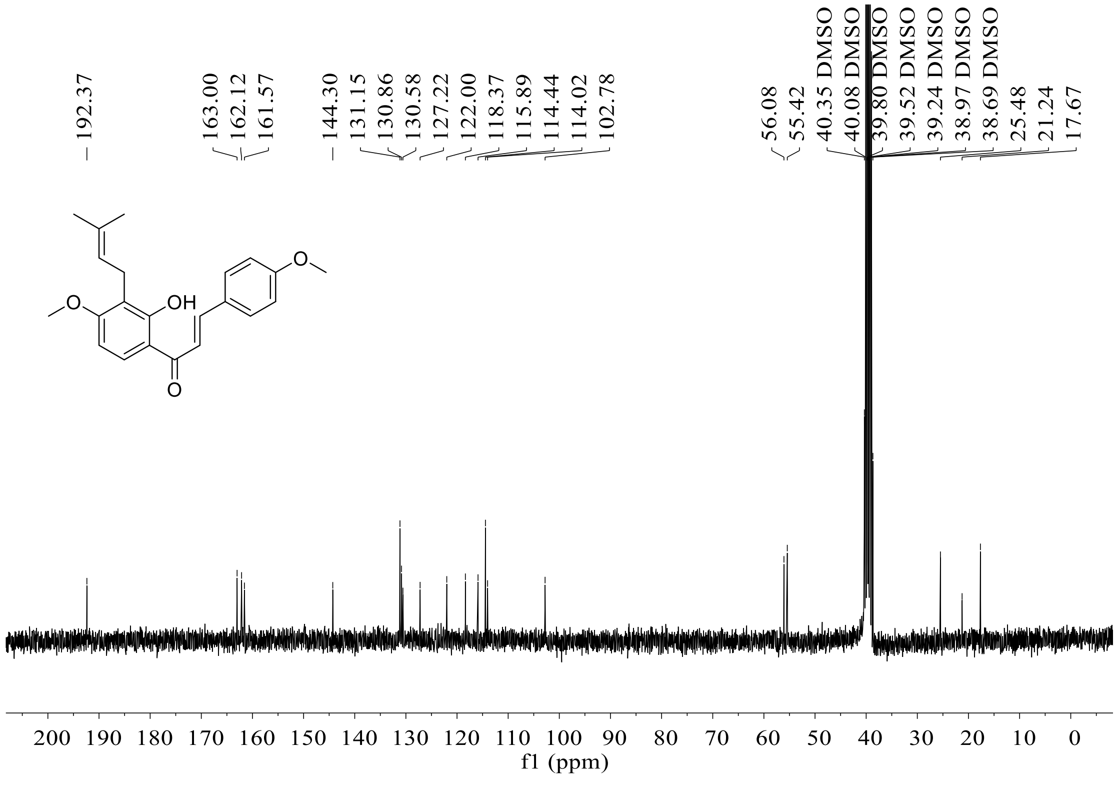

Figure S8 ^{13}C-NMR (75 MHz, DMSO-d_6) spectrum of compound 3

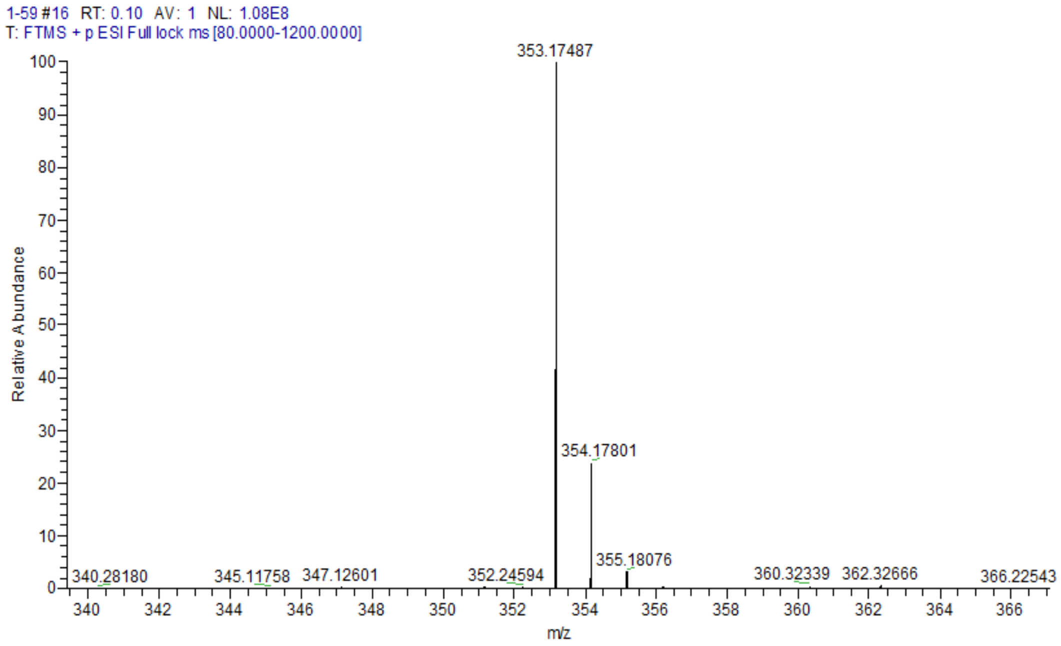

Figure S9 ESI-HRMS spectrum of compound 3

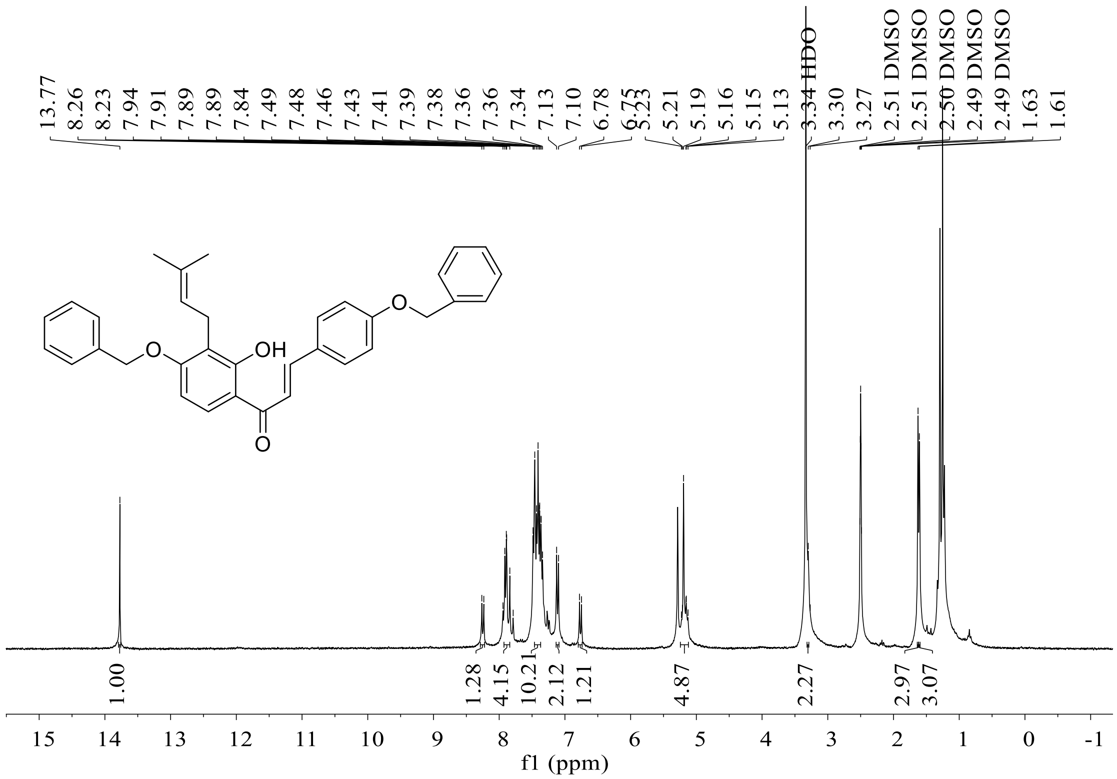

Figure S10 ^{1}H-NMR (300 MHz, DMSO-d_6) spectrum of compound 4

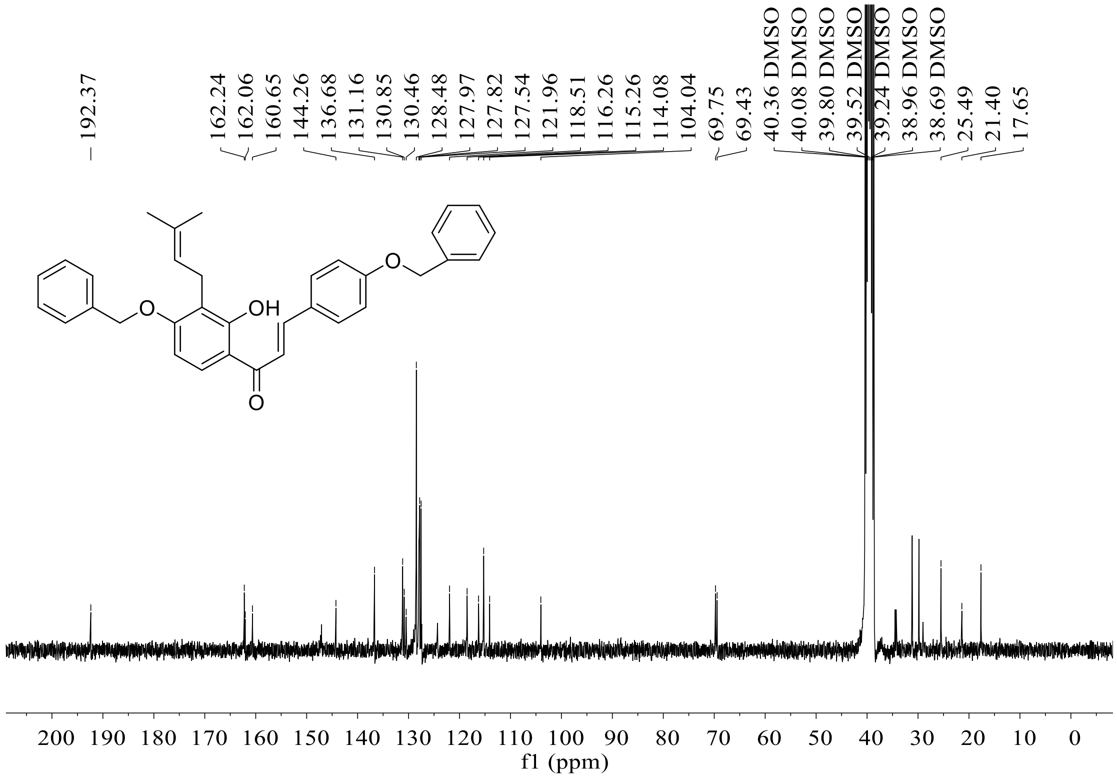

Figure S11 ^{13}C-NMR (75 MHz, DMSO-d_6) spectrum of compound 4

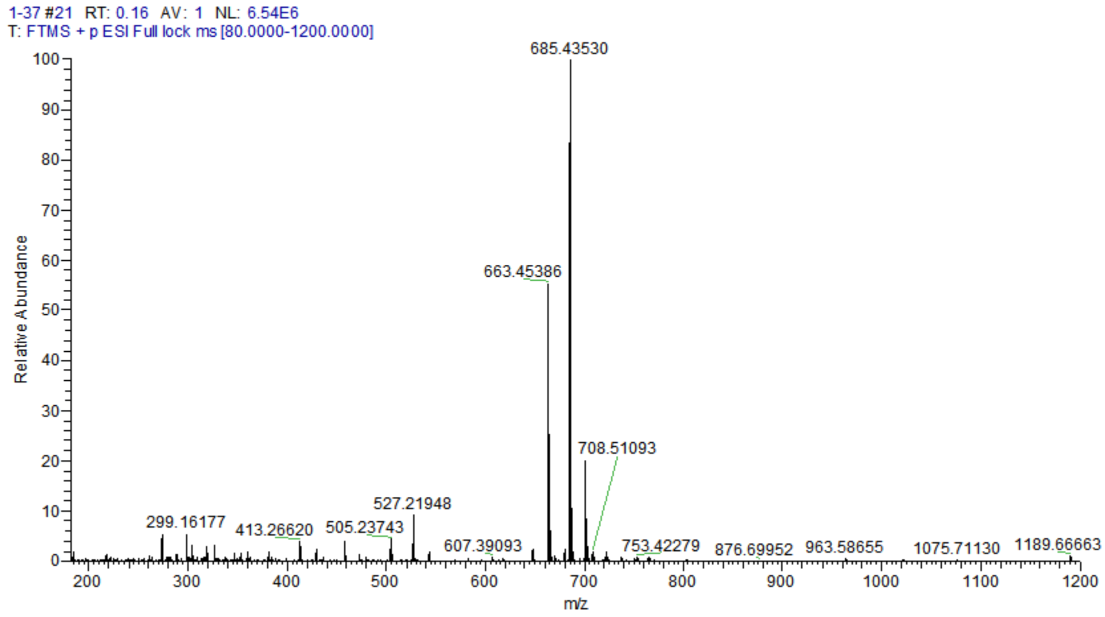

Figure S12 ESI-HRMS spectrum of compound 4

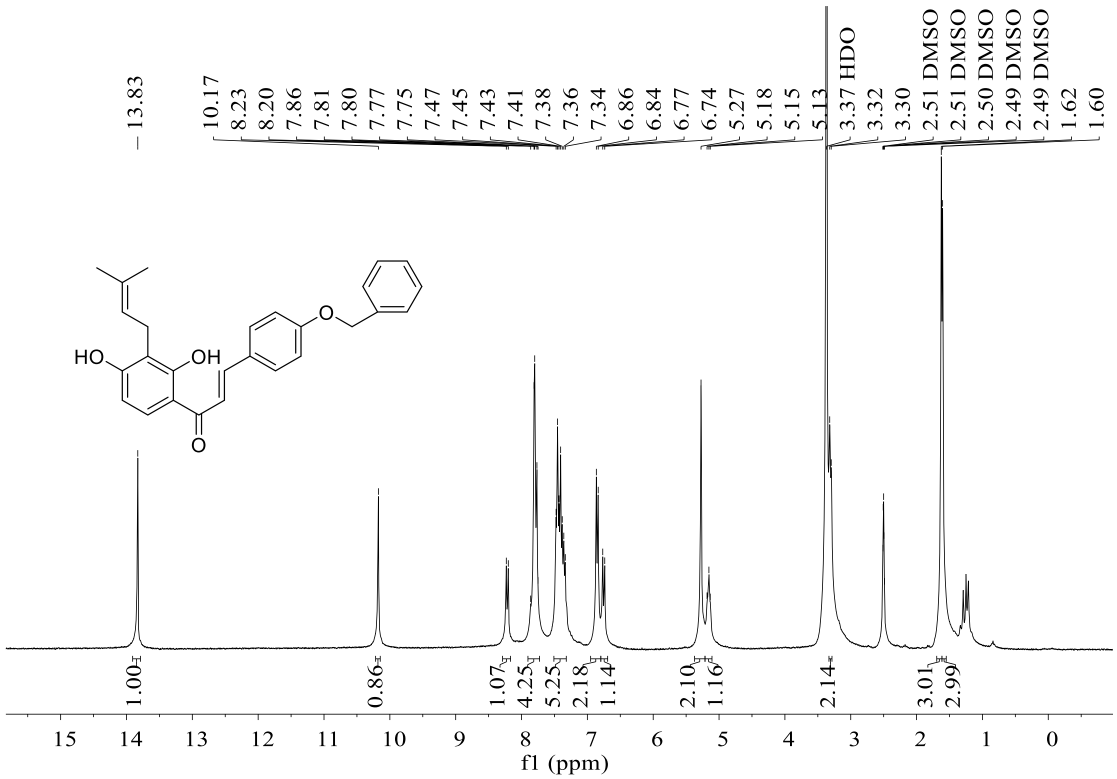

Figure S13 ^{1}H-NMR (300 MHz, DMSO-*d$_6$*) spectrum of compound 5

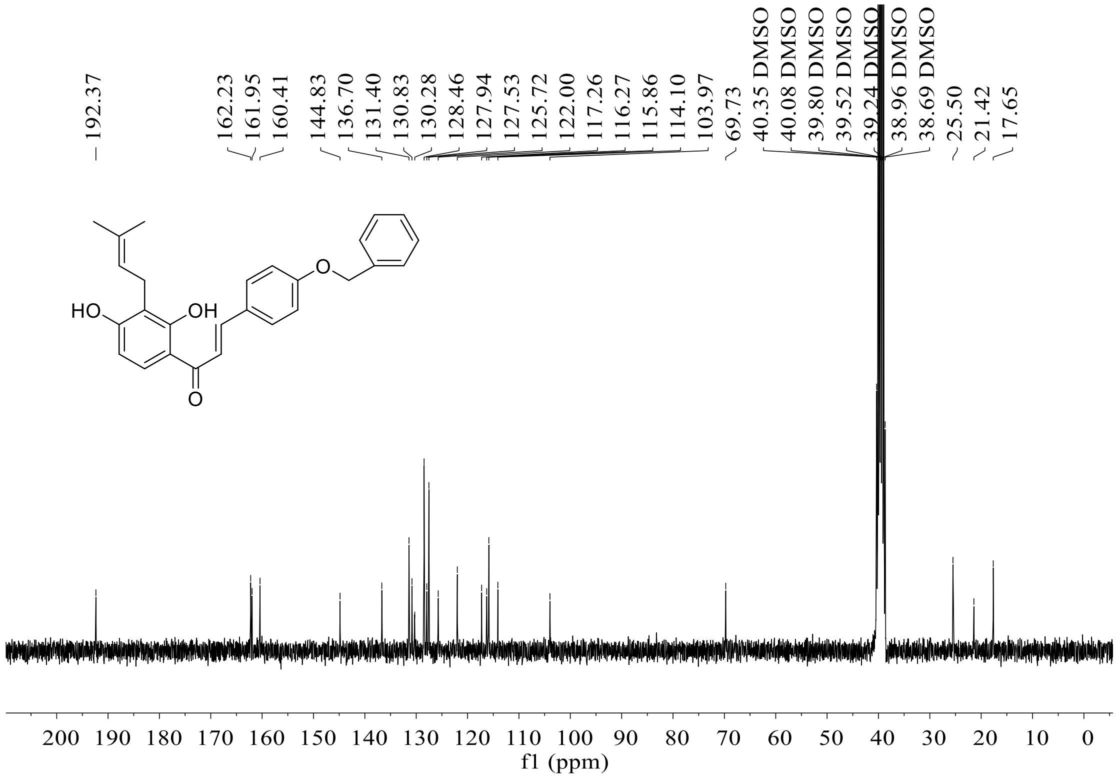

Figure S14 ^{13}C-NMR (75 MHz, DMSO-*d$_6$*) spectrum of compound 5

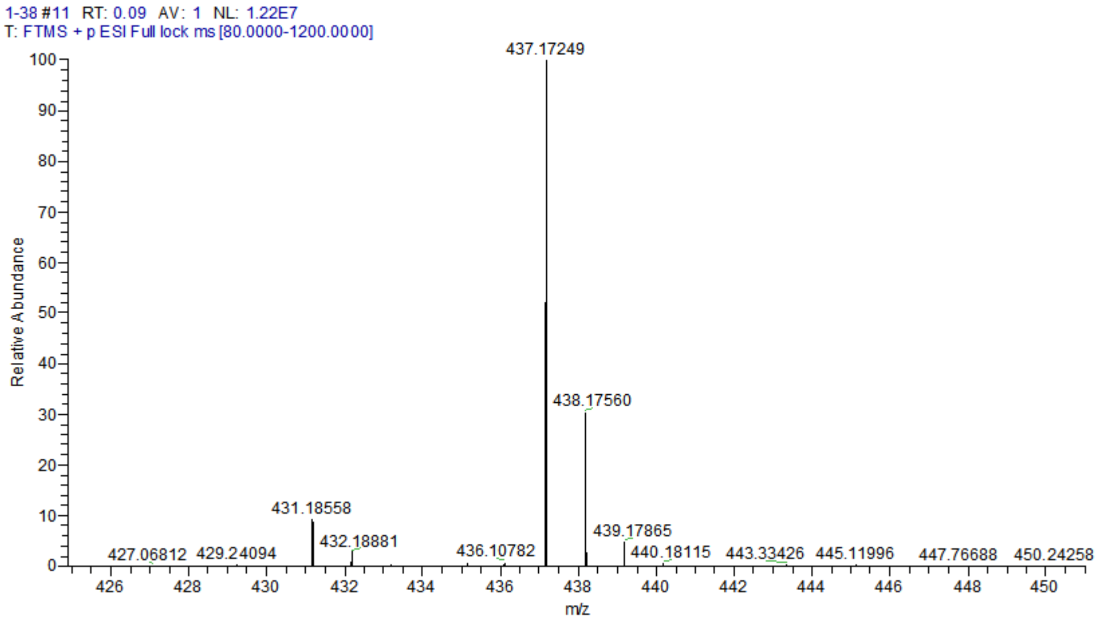

Figure S15 ESI-HRMS spectrum of compound 5

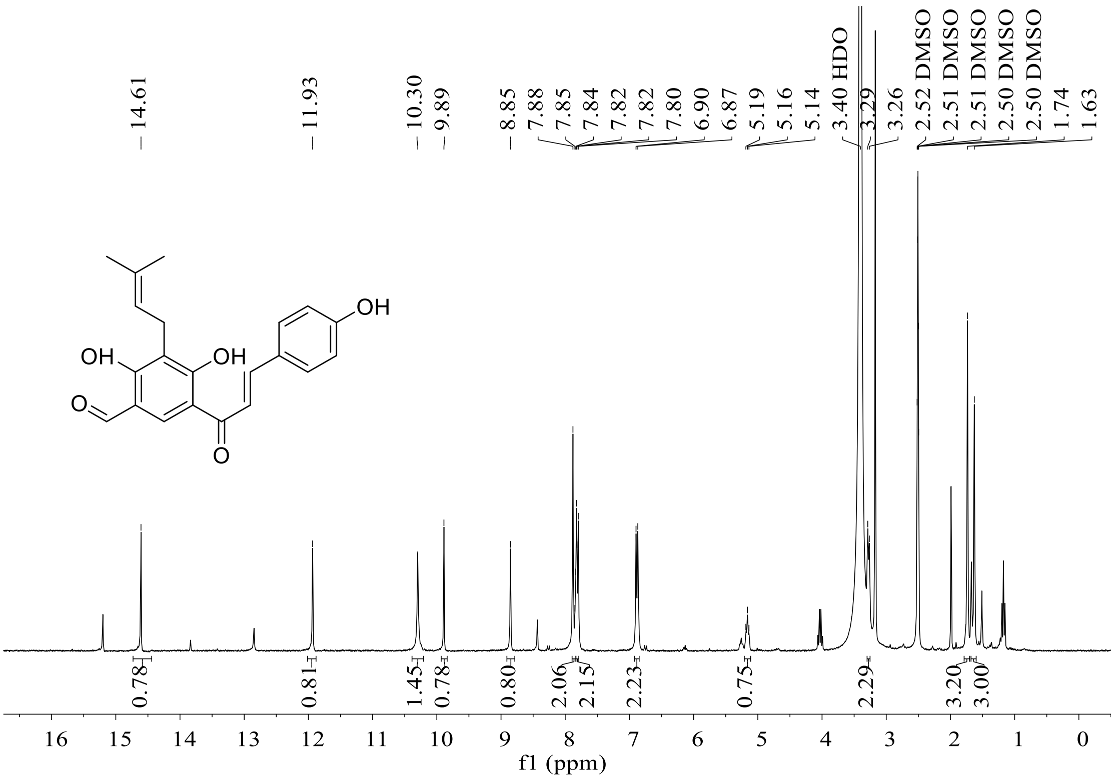

Figure S16 ^{1}H-NMR (300 MHz, DMSO-d_6) spectrum of compound 6

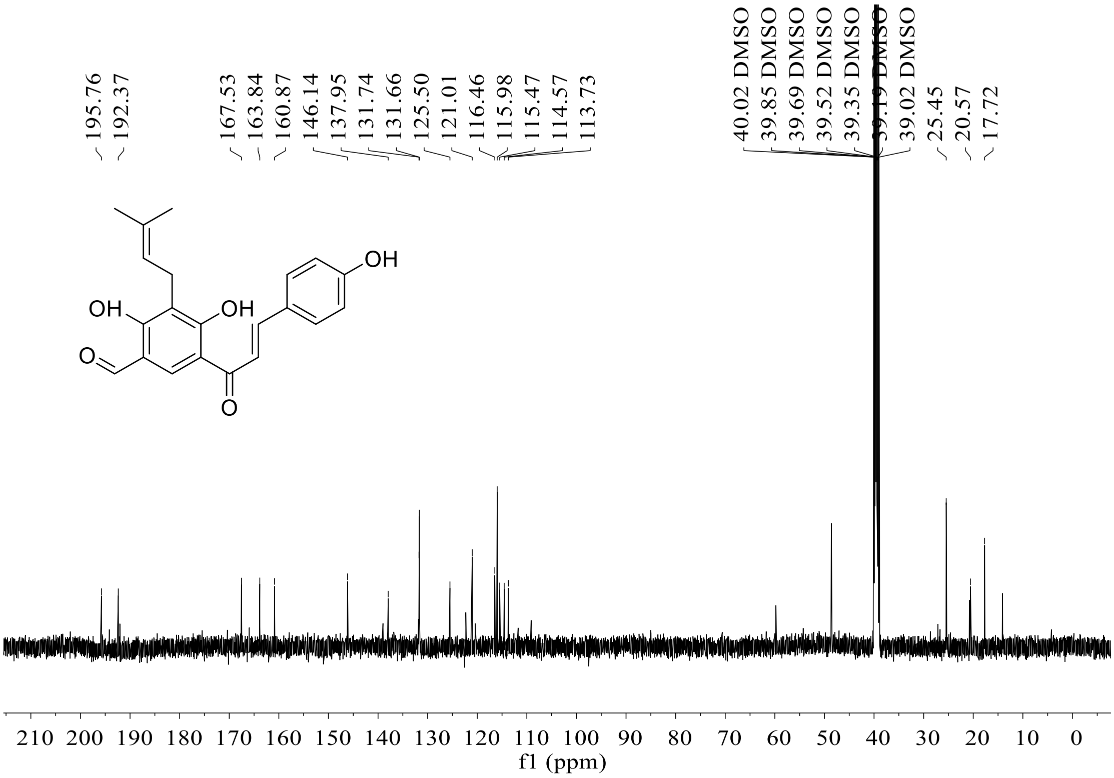

Figure S17 ^{13}C-NMR (75 MHz, DMSO-d_6) spectrum of compound 6

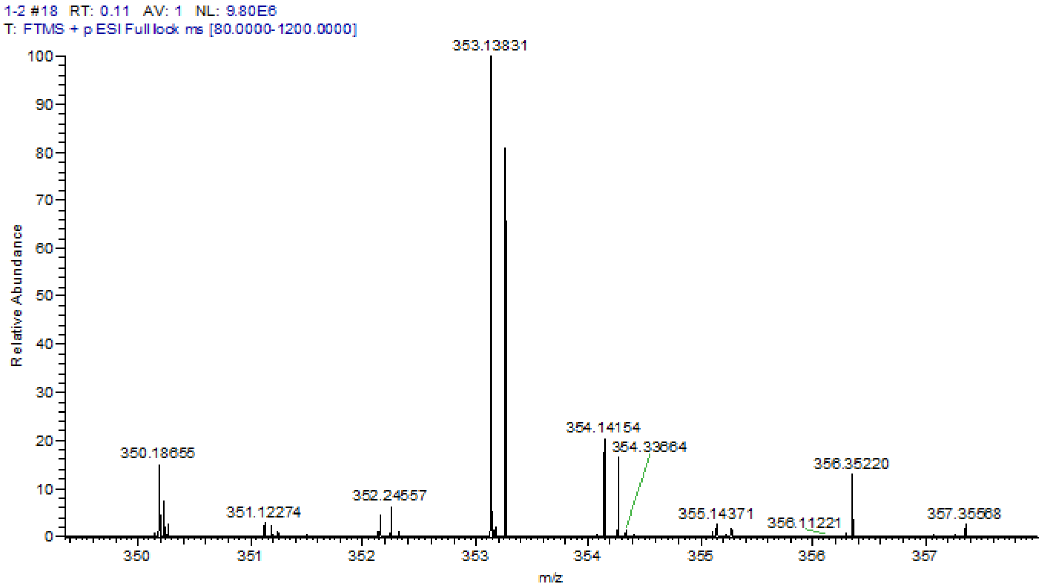

Figure S18 ESI-HRMS spectrum of compound 6

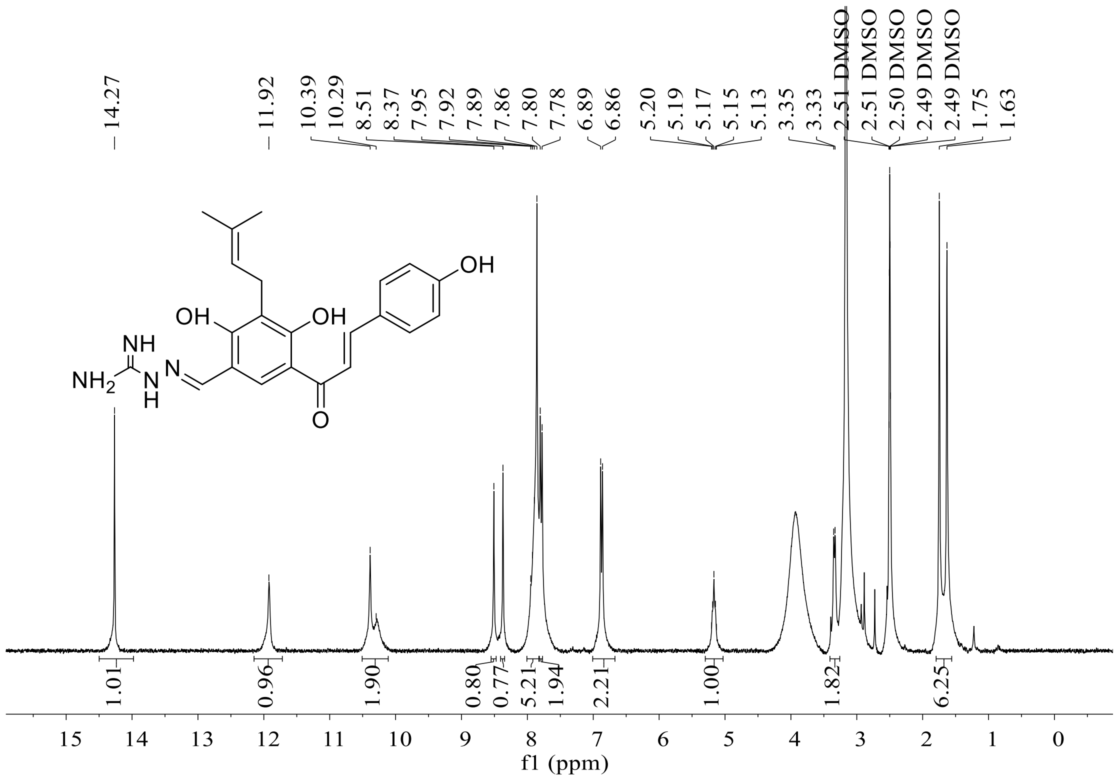

Figure S19 ^{1}H-NMR (300 MHz, DMSO-d_6) spectrum of compound 7

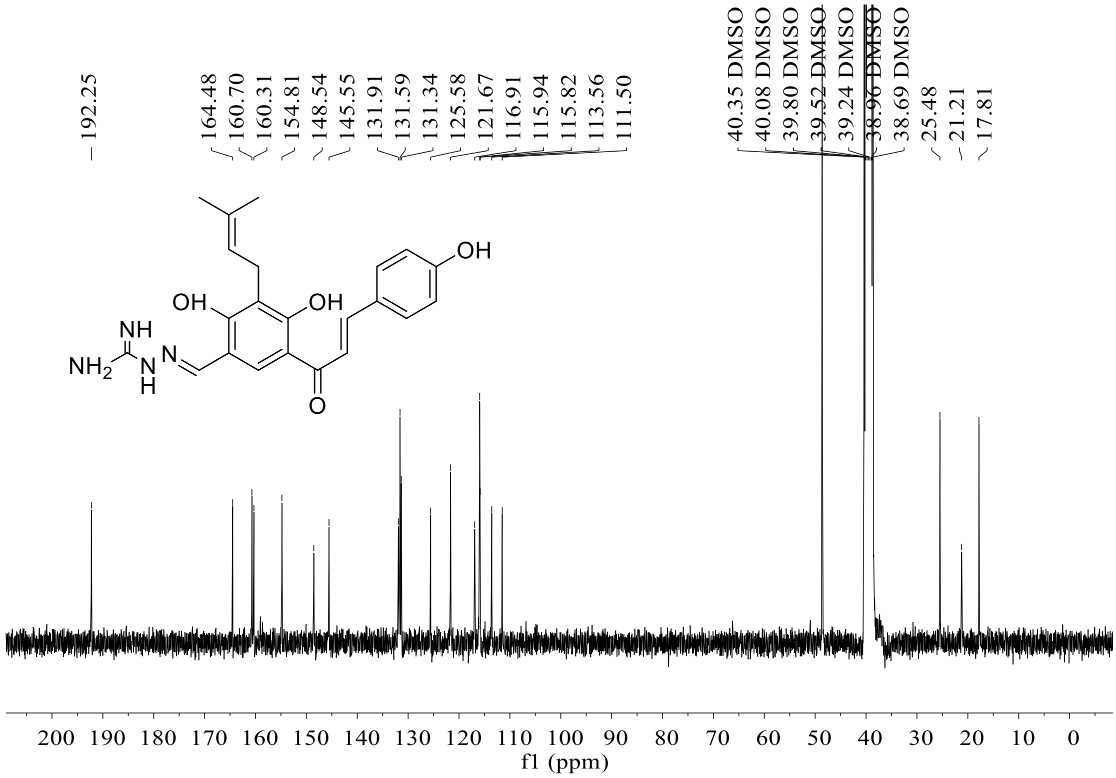

Figure S20 ^{13}C-NMR (75 MHz, DMSO-d_6) spectrum of compound 7

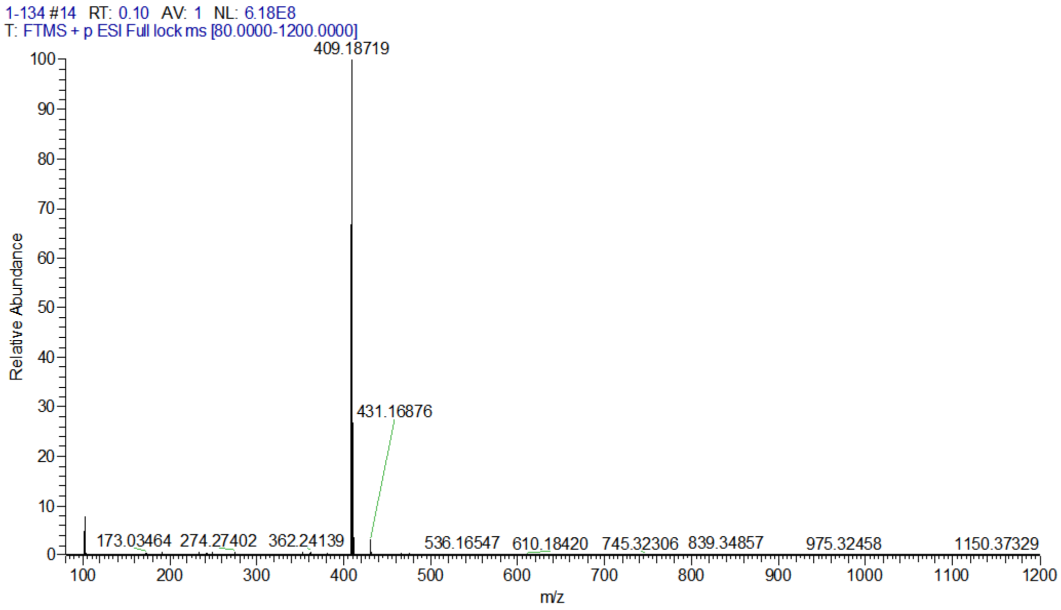

Figure S21 ESI-HRMS spectrum of compound 7

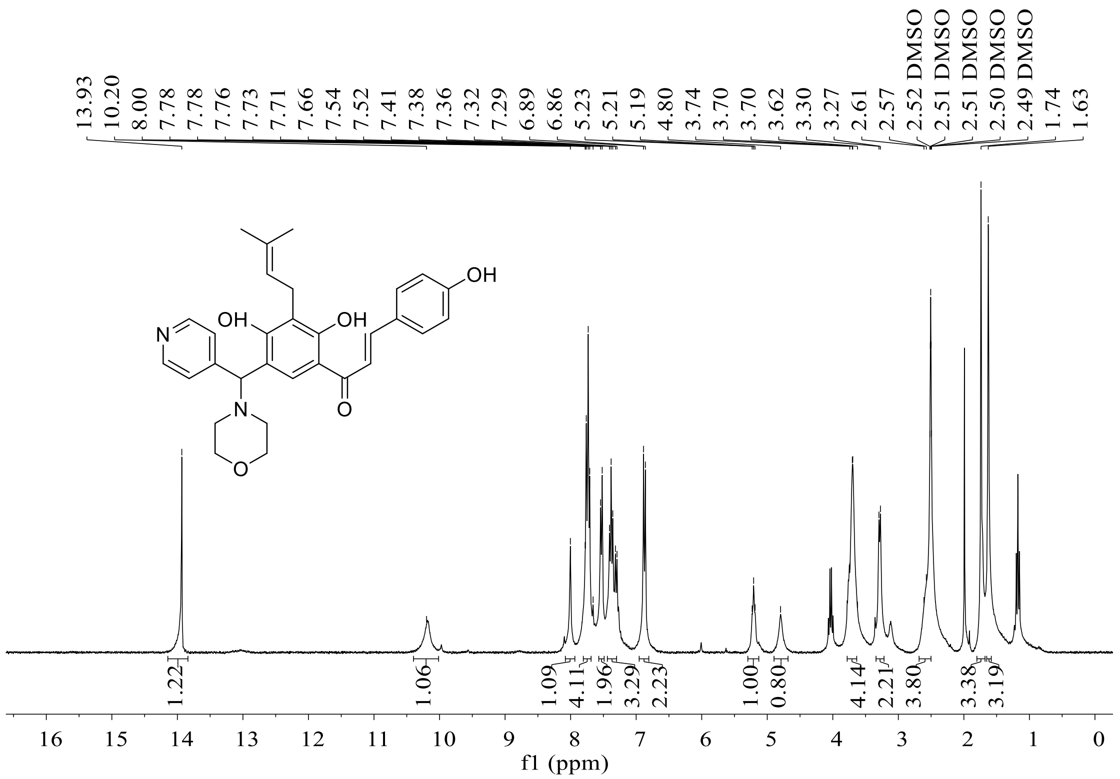

Figure S22 ^{1}H-NMR (300 MHz, DMSO-d_6) spectrum of compound 8

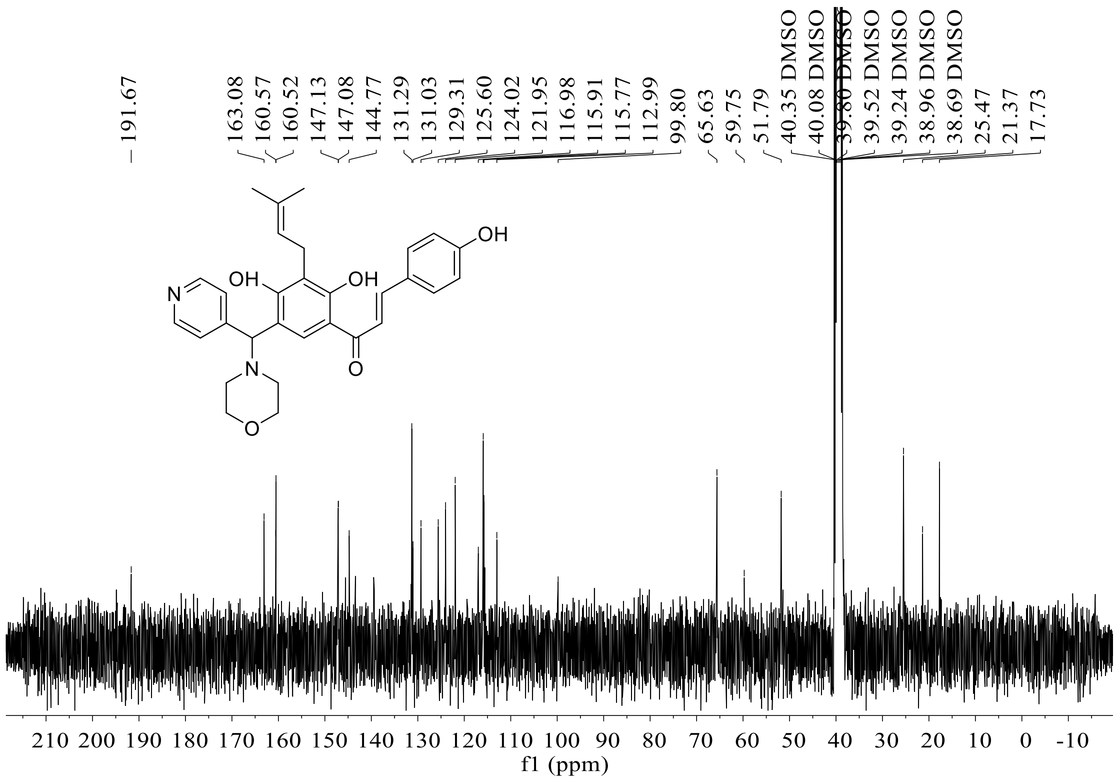

Figure S23 ^{13}C-NMR (75 MHz, DMSO-d_6) spectrum of compound 8

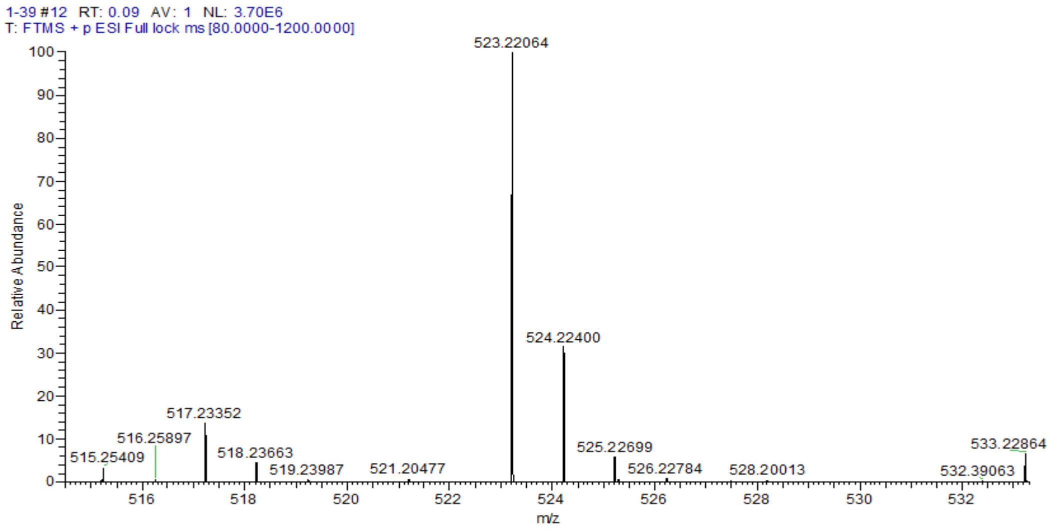

Figure S24 ESI-HRMS spectrum of compound 8

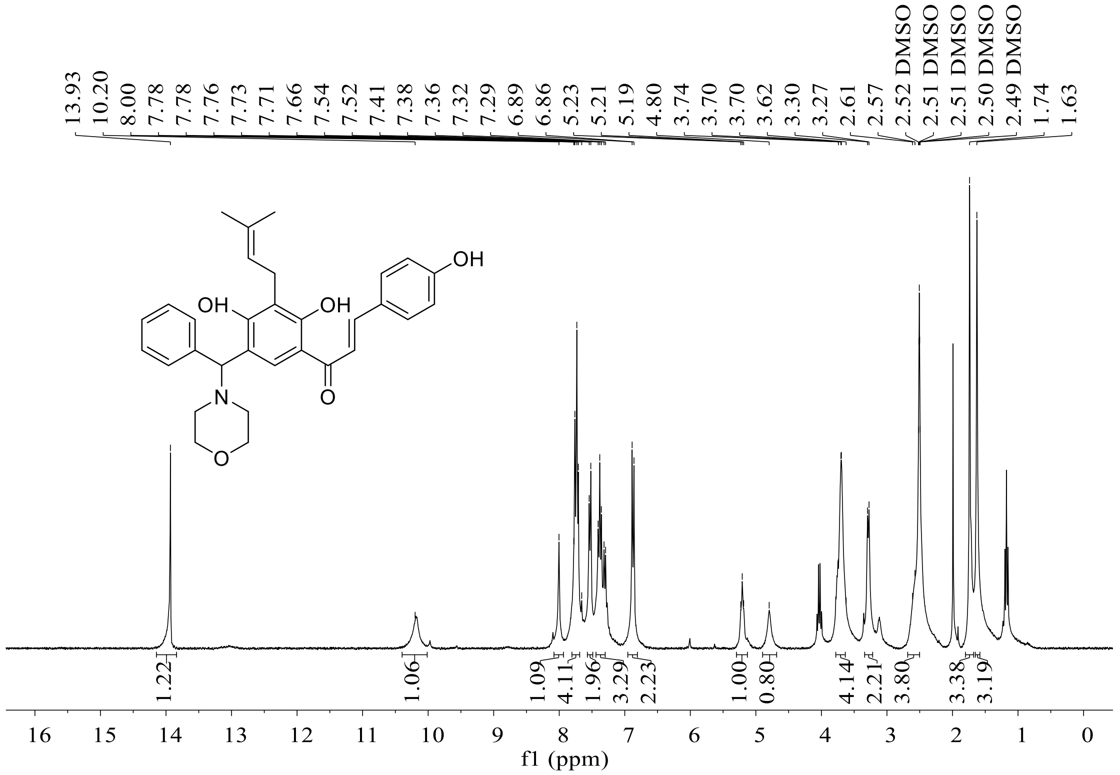

Figure S25 ^{1}H-NMR (300 MHz, DMSO-d_6) spectrum of compound 9

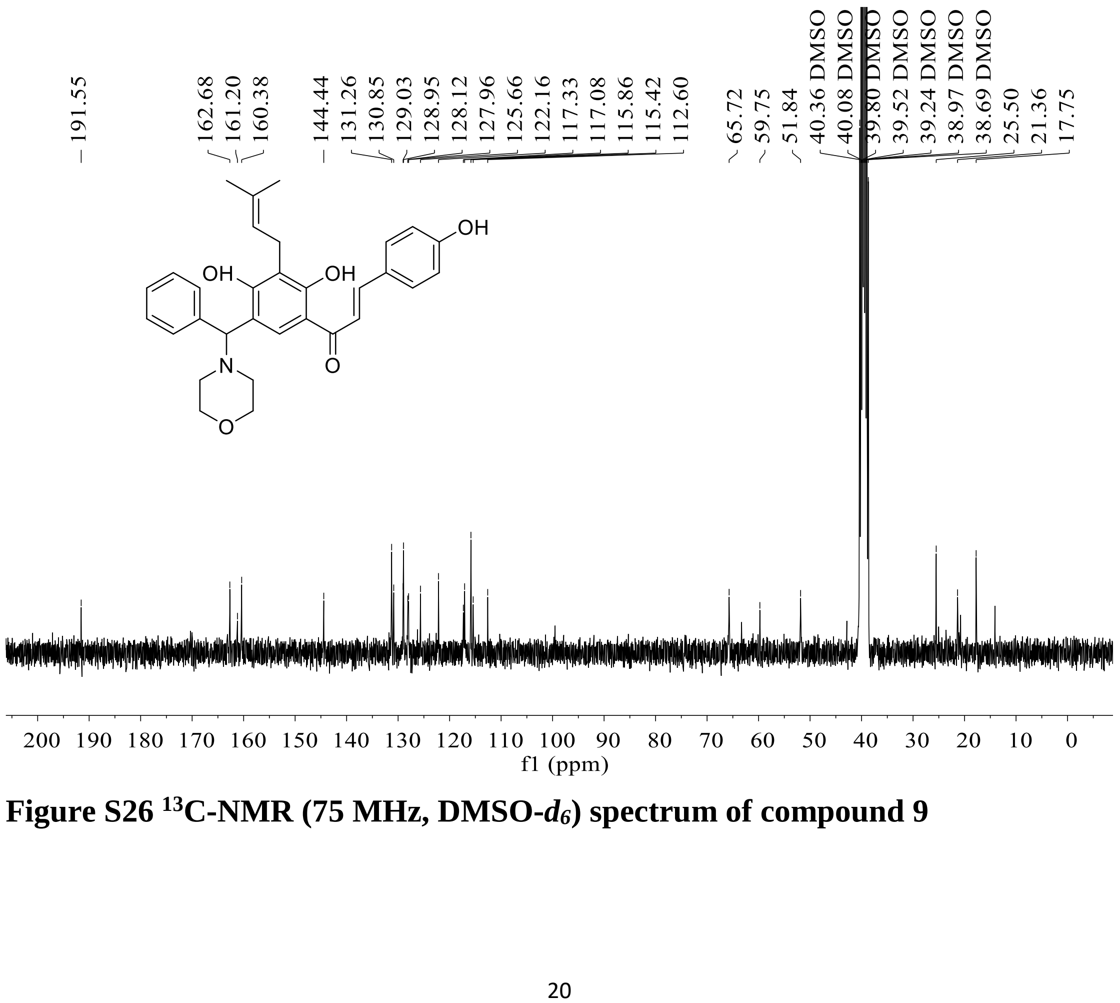

Figure S26 ^{13}C-NMR (75 MHz, DMSO-d_6) spectrum of compound 9

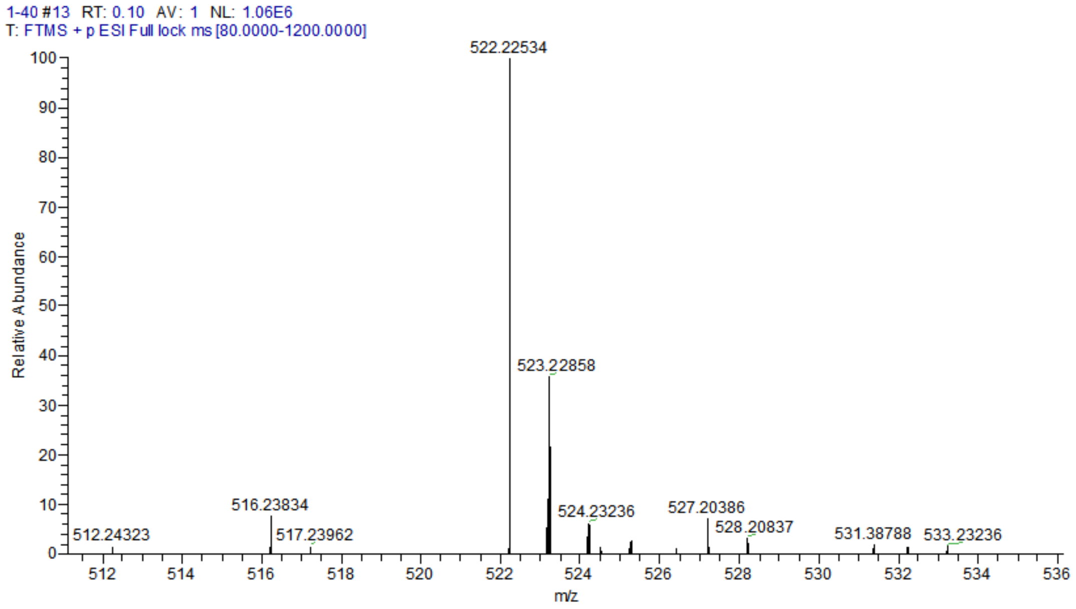

Figure S27 ESI-HRMS spectrum of compound 9

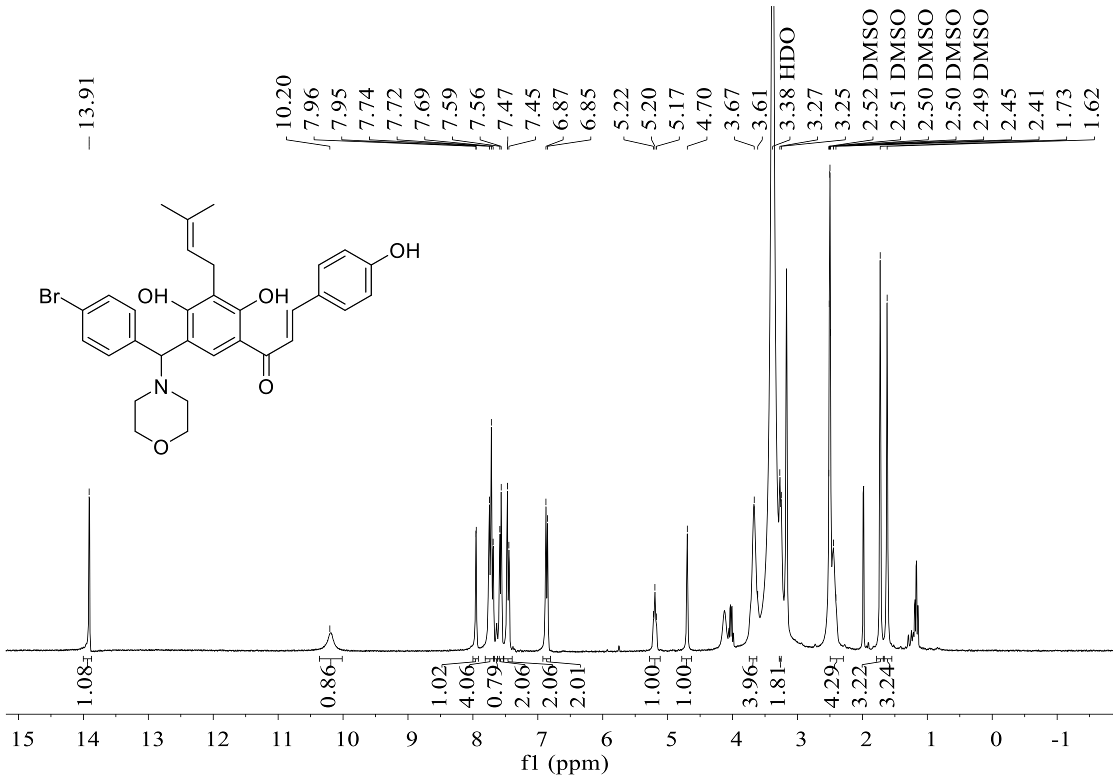

Figure S28 ^{1}H-NMR (300 MHz, DMSO-d_6) spectrum of compound 10

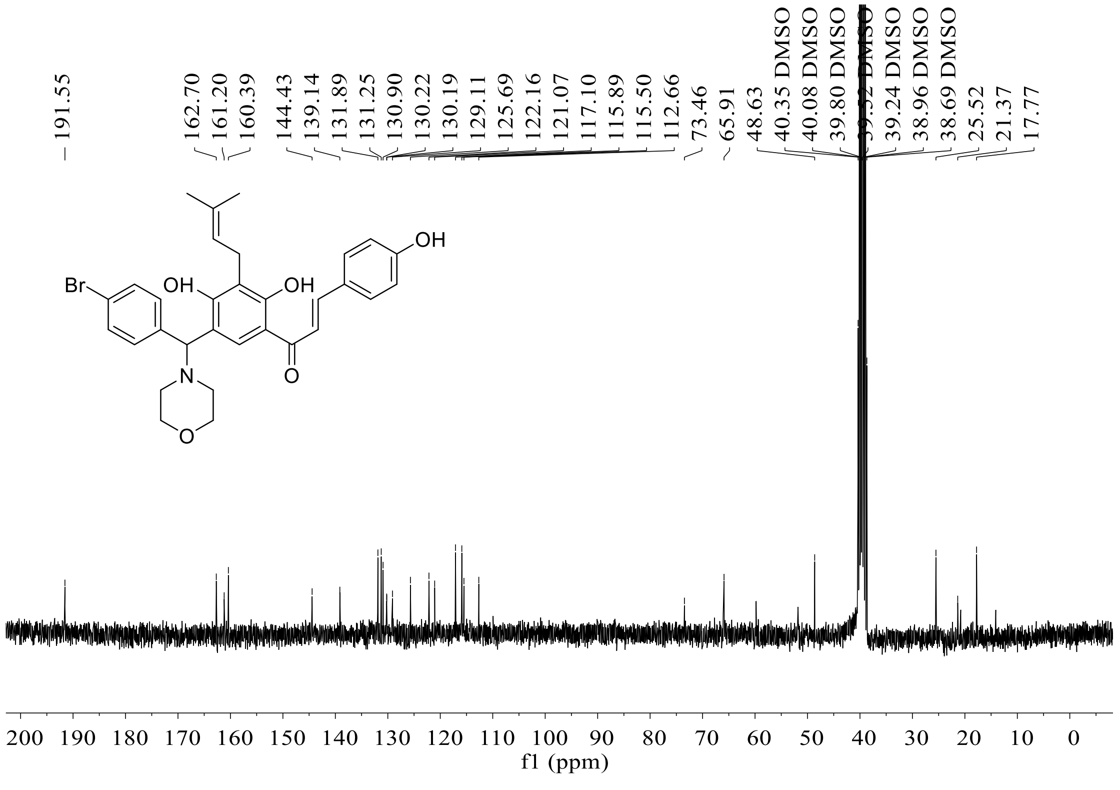

Figure S29 ^{13}C-NMR (75 MHz, DMSO-d_6) spectrum of compound 10

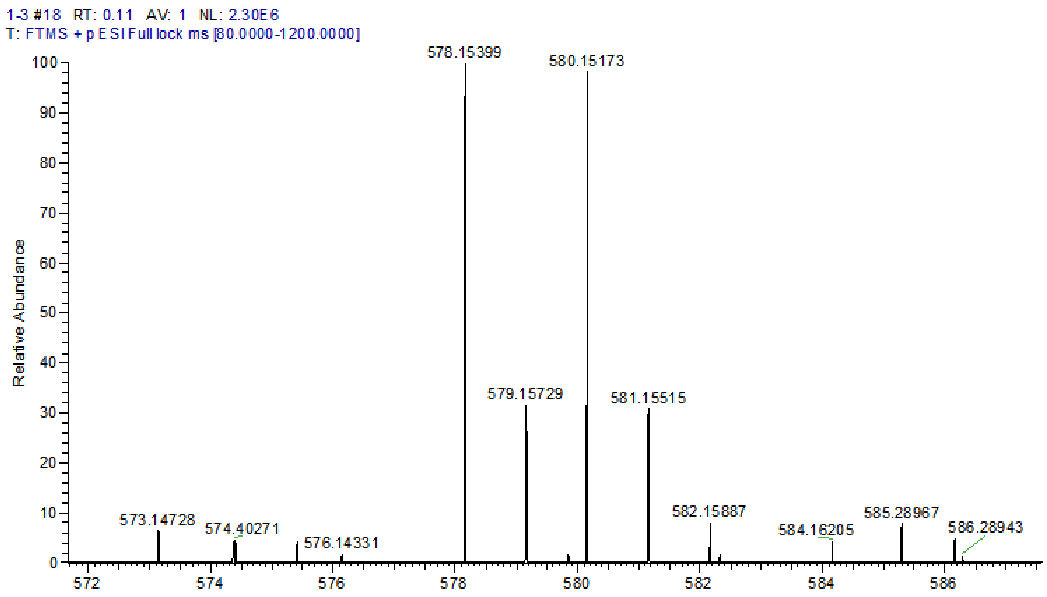

Figure S30 ESI-HRMS spectrum of compound 10

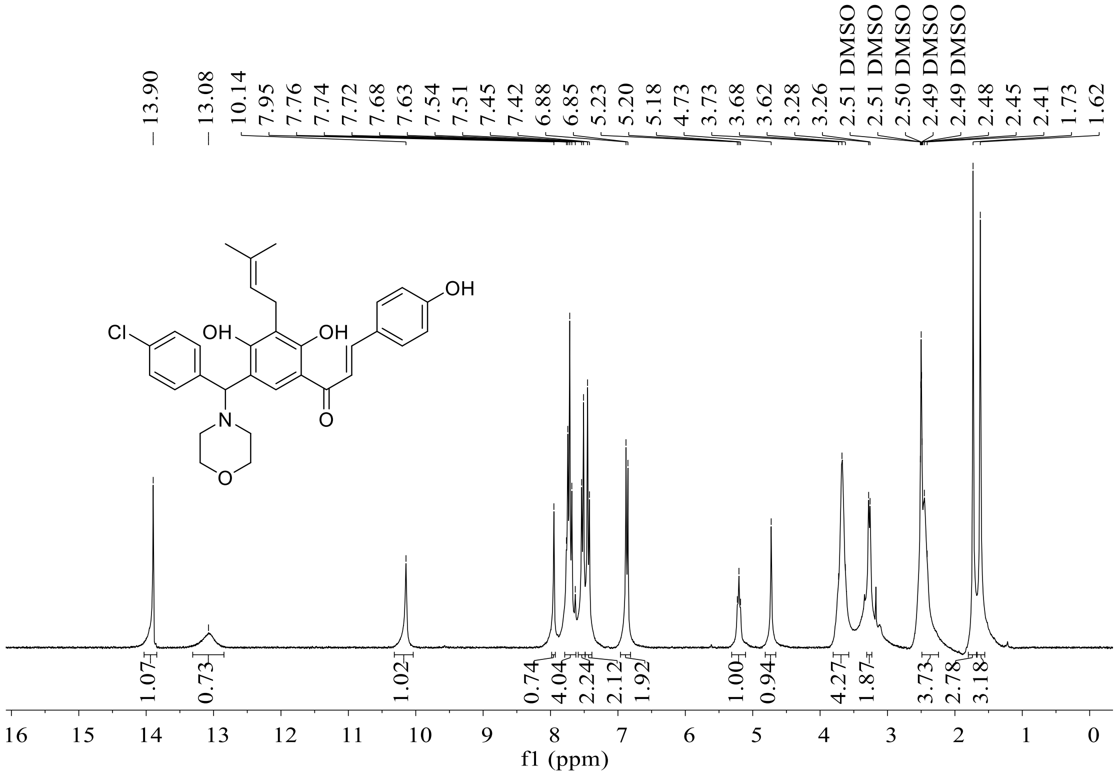

Figure S31 ^{1}H-NMR (300 MHz, DMSO-d_6) spectrum of compound 11

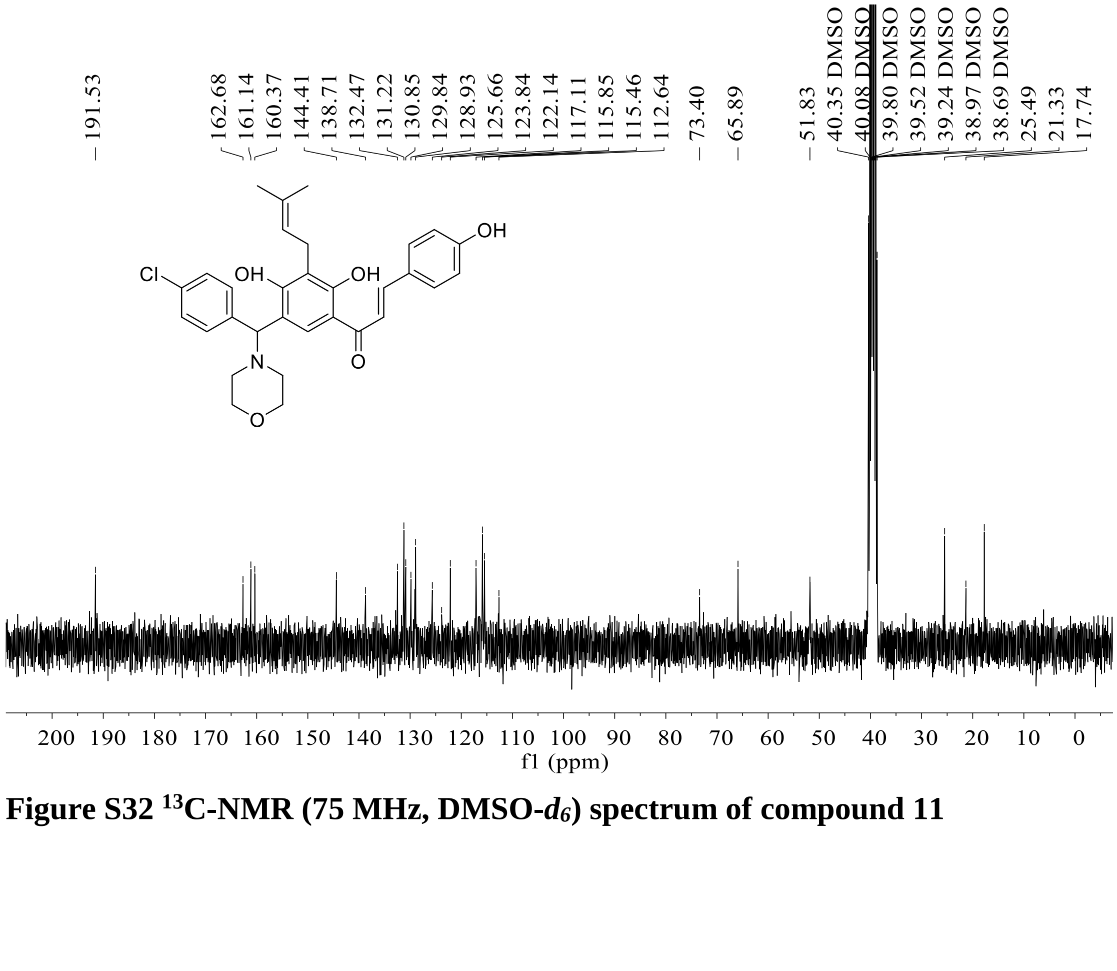

Figure S32 ^{13}C-NMR (75 MHz, DMSO-d_6) spectrum of compound 11

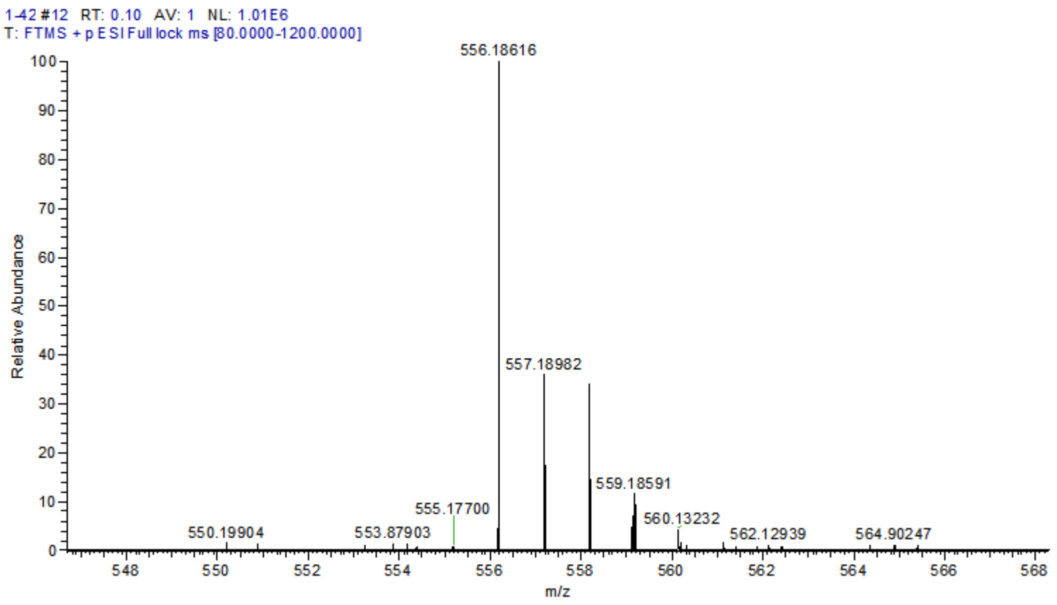

Figure S33 ESI-HRMS spectrum of compound 11

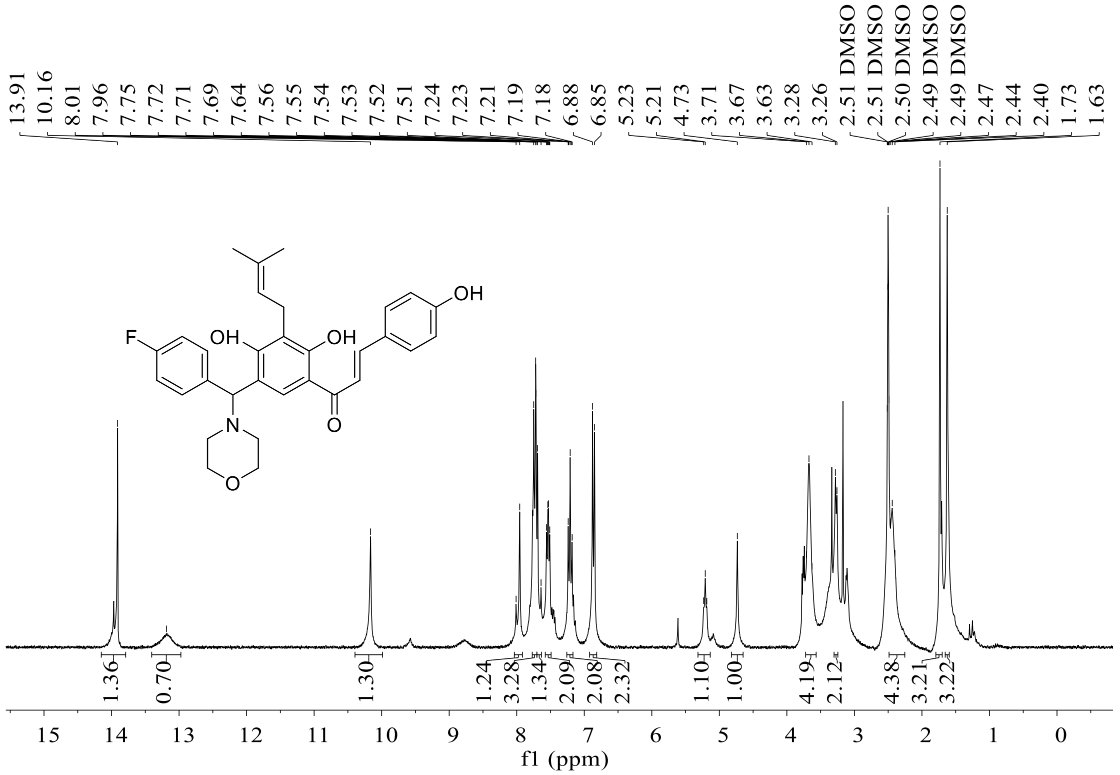

Figure S34 ^{1}H-NMR (300 MHz, DMSO-d_6) spectrum of compound 12

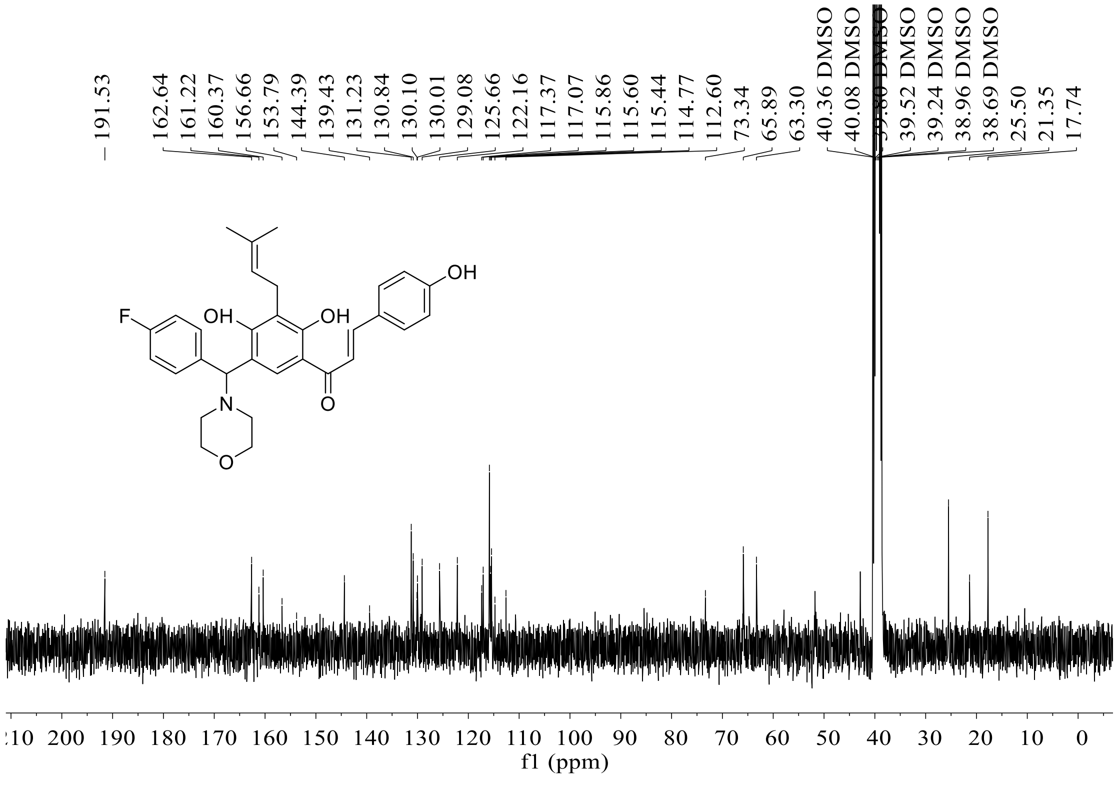

Figure S35 ^{13}C-NMR (75 MHz, DMSO-d_6) spectrum of compound 12

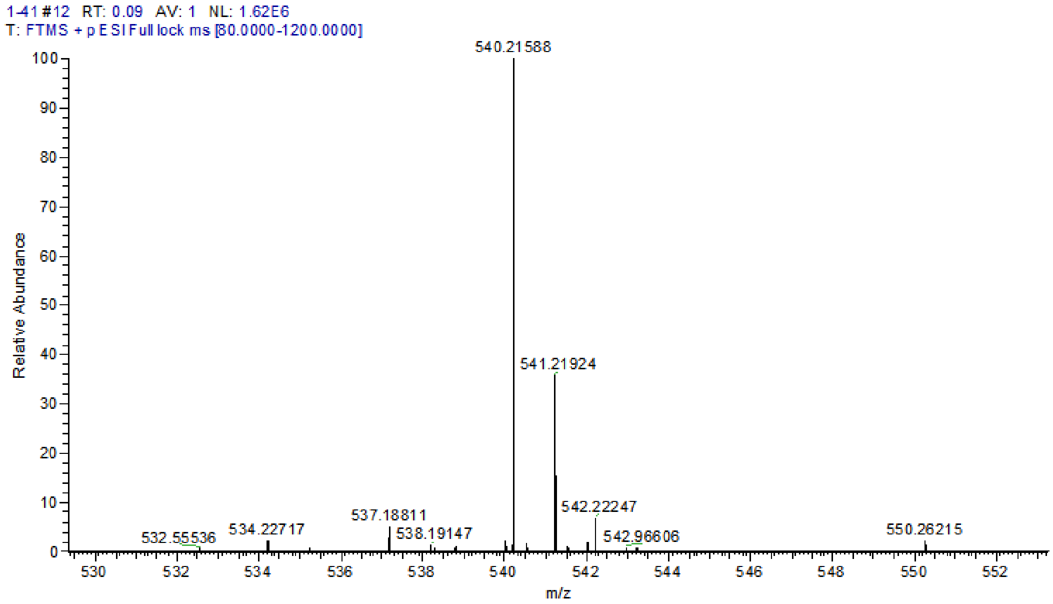

Figure S36 ESI-HRMS spectrum of compound 12

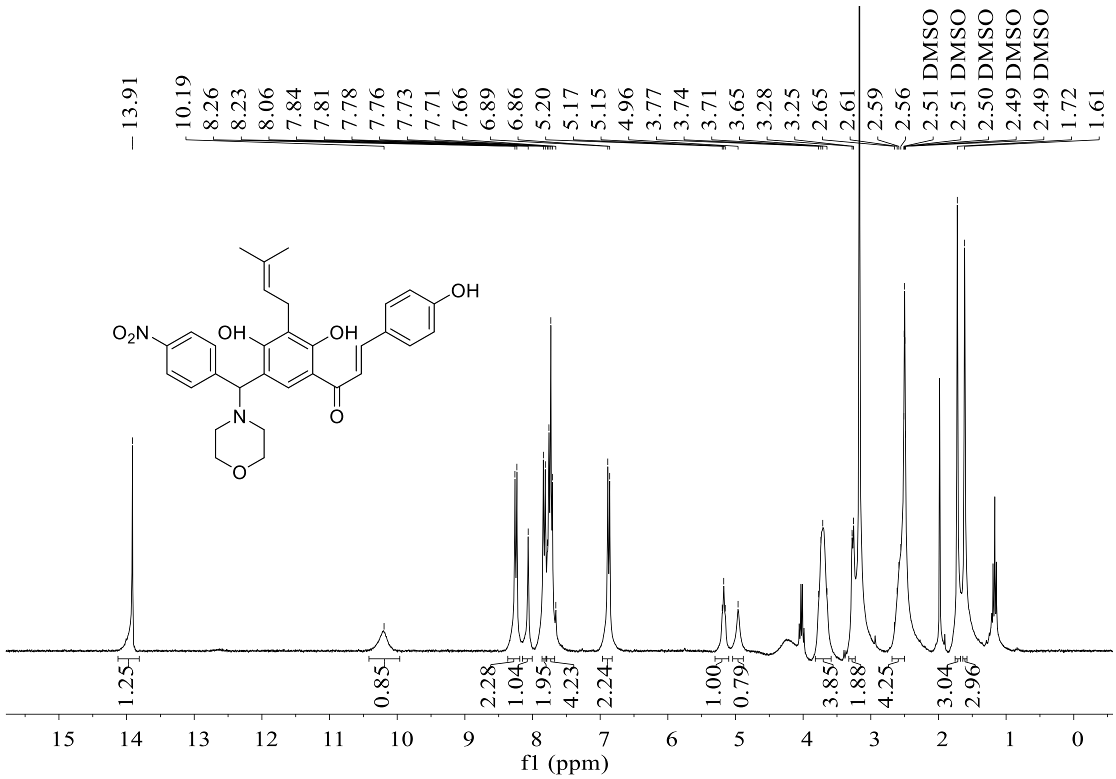

Figure S37 ^{1}H-NMR (300 MHz, DMSO-d_6) spectrum of compound 13

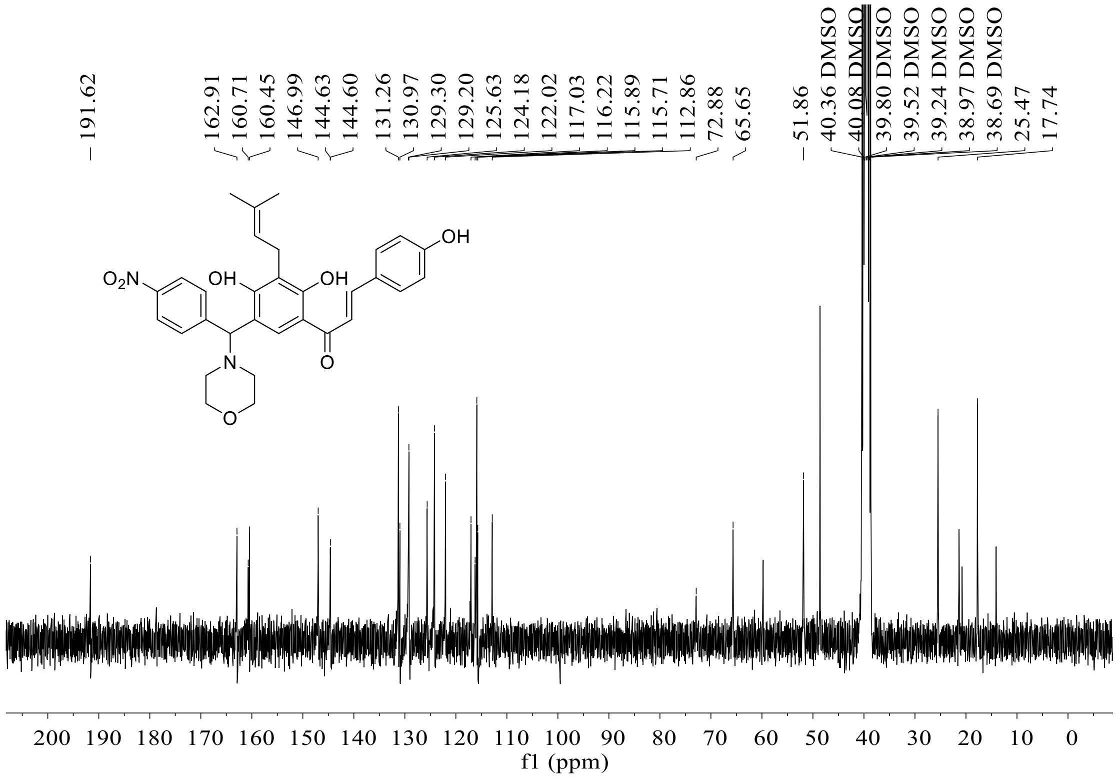

Figure S38 ^{13}C-NMR (75 MHz, DMSO-d_6) spectrum of compound 13

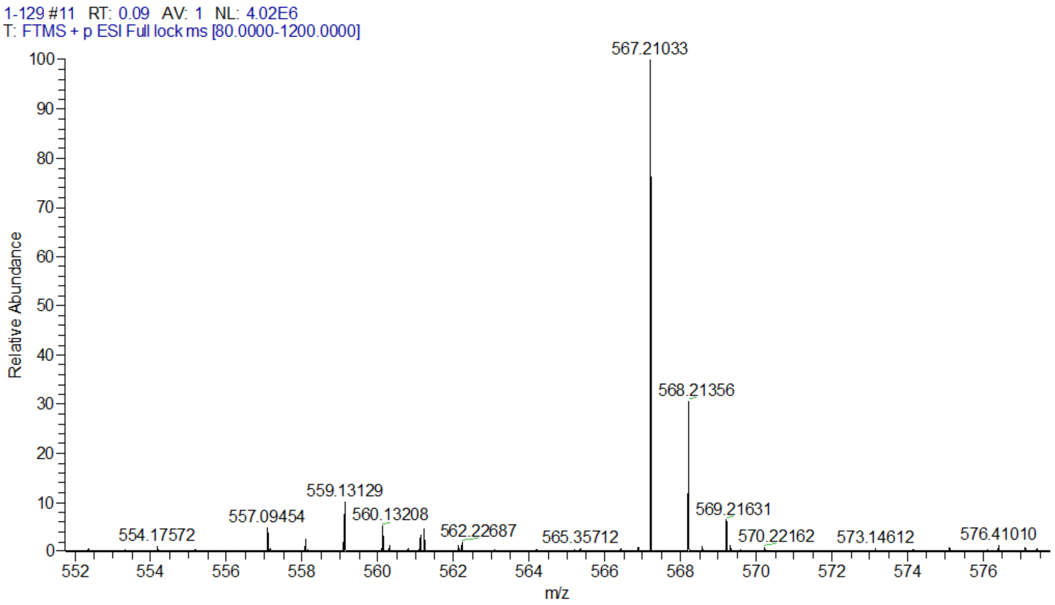

Figure S39 ESI-HRMS spectrum of compound 13

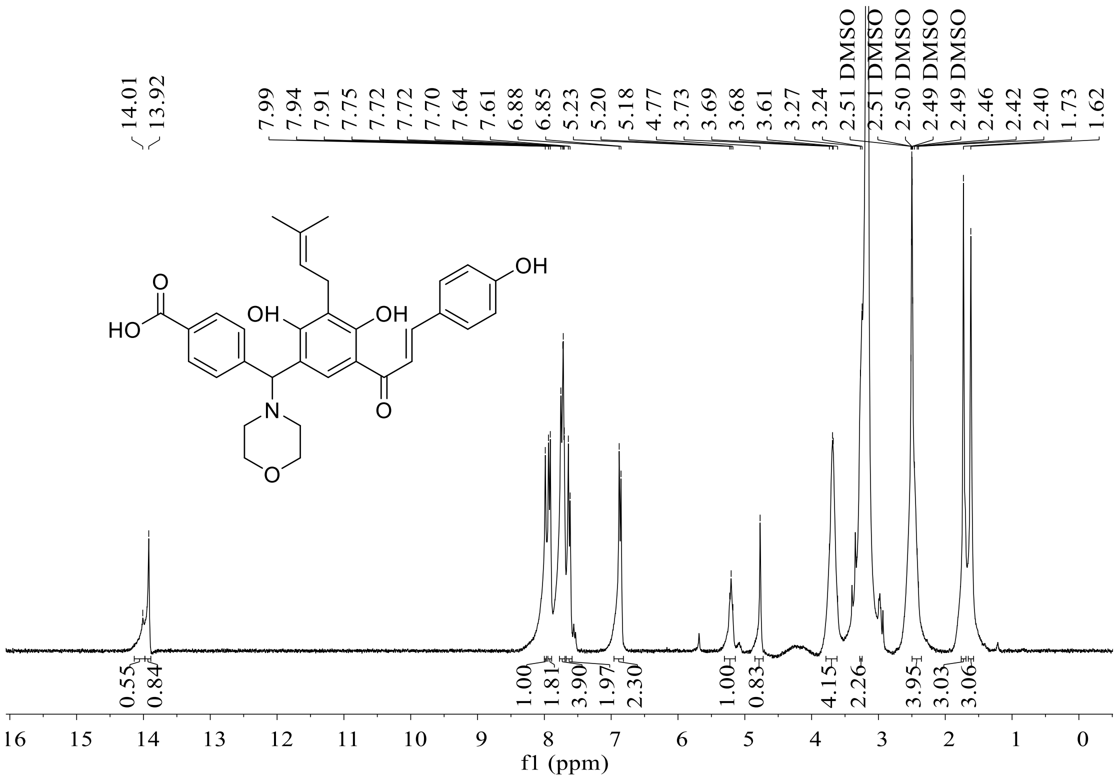

Figure S40 ^{1}H-NMR (300 MHz, DMSO-d_6) spectrum of compound 14

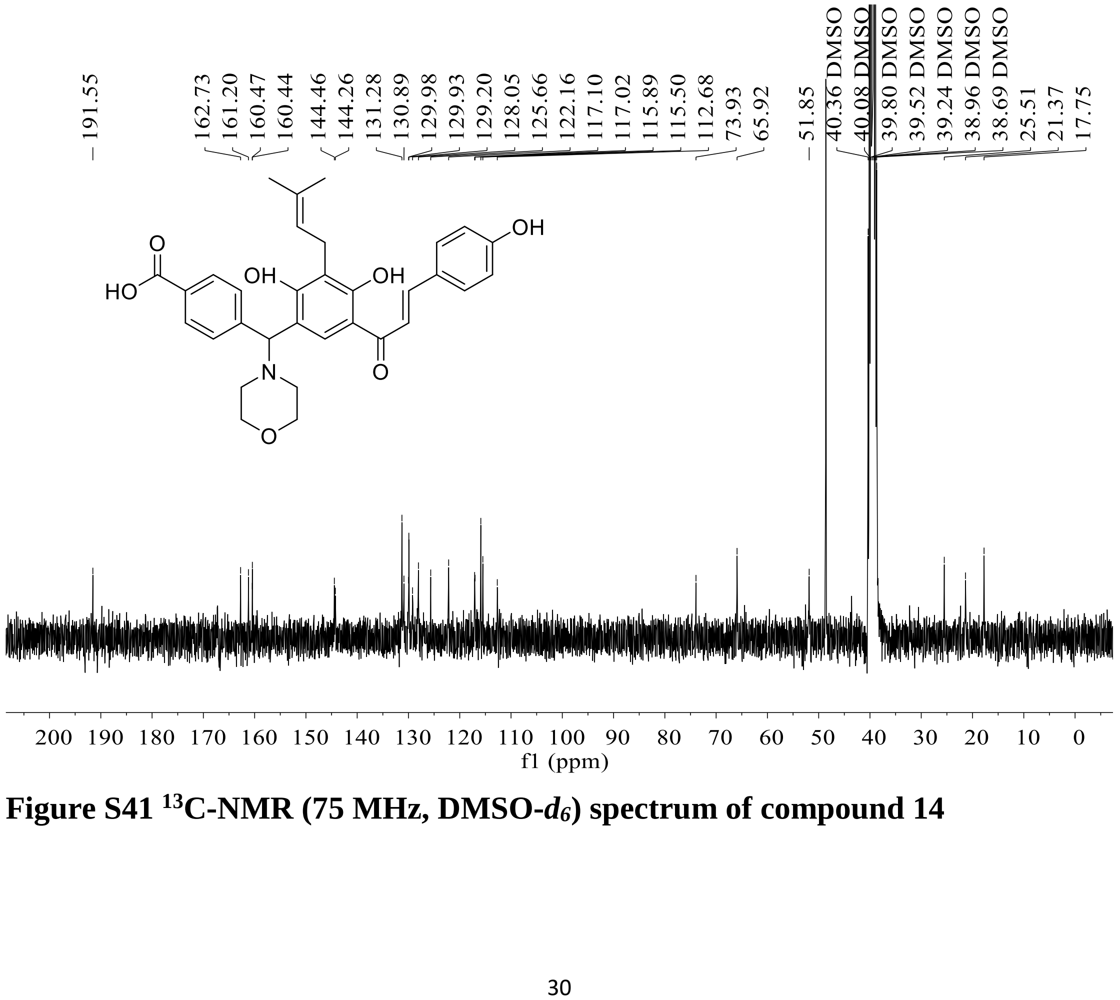

Figure S41 ^{13}C-NMR (75 MHz, DMSO-d_6) spectrum of compound 14

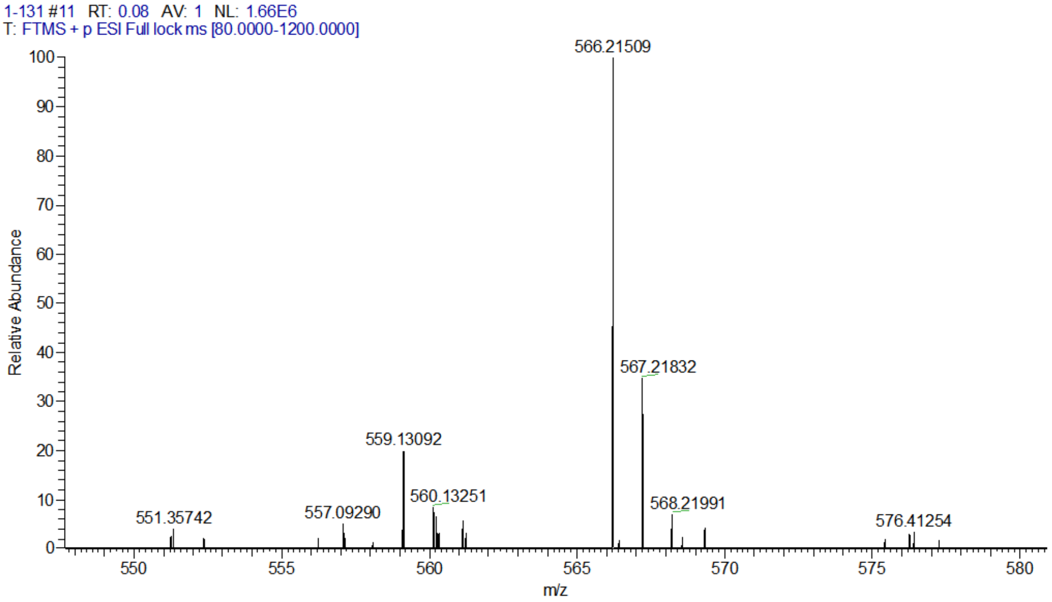

Figure S42 ESI-HRMS spectrum of compound 14

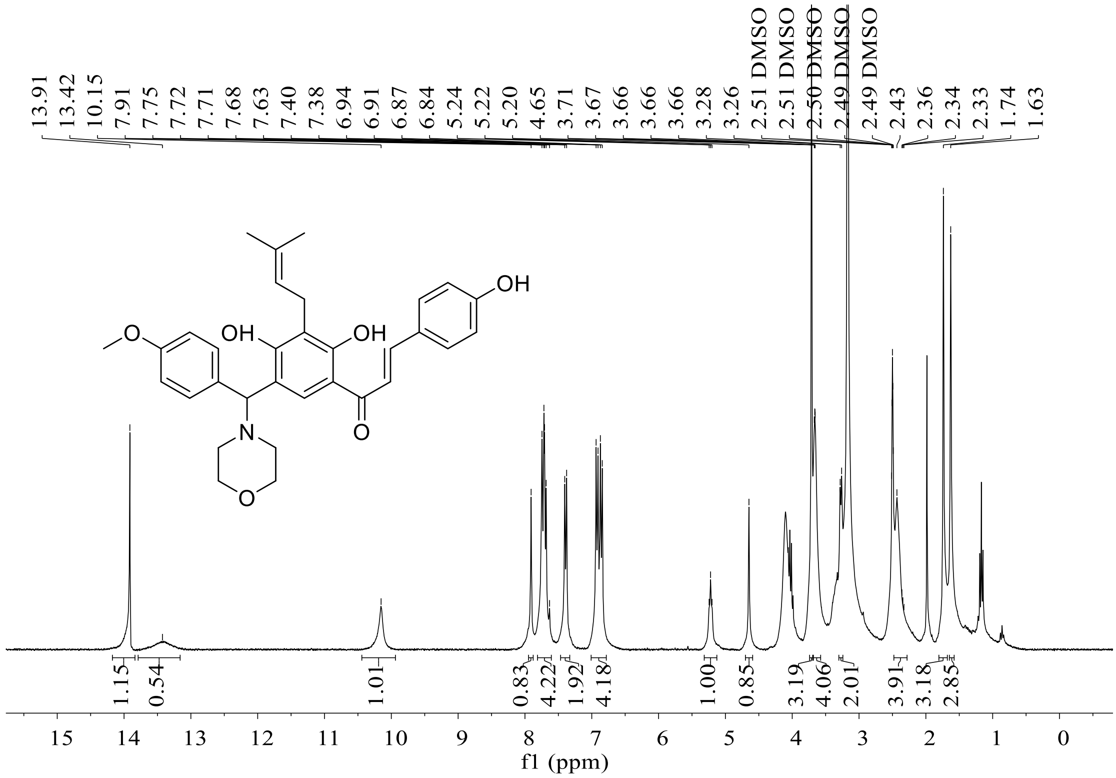

Figure S43 ^{1}H-NMR (300 MHz, DMSO-d_6) spectrum of compound 15

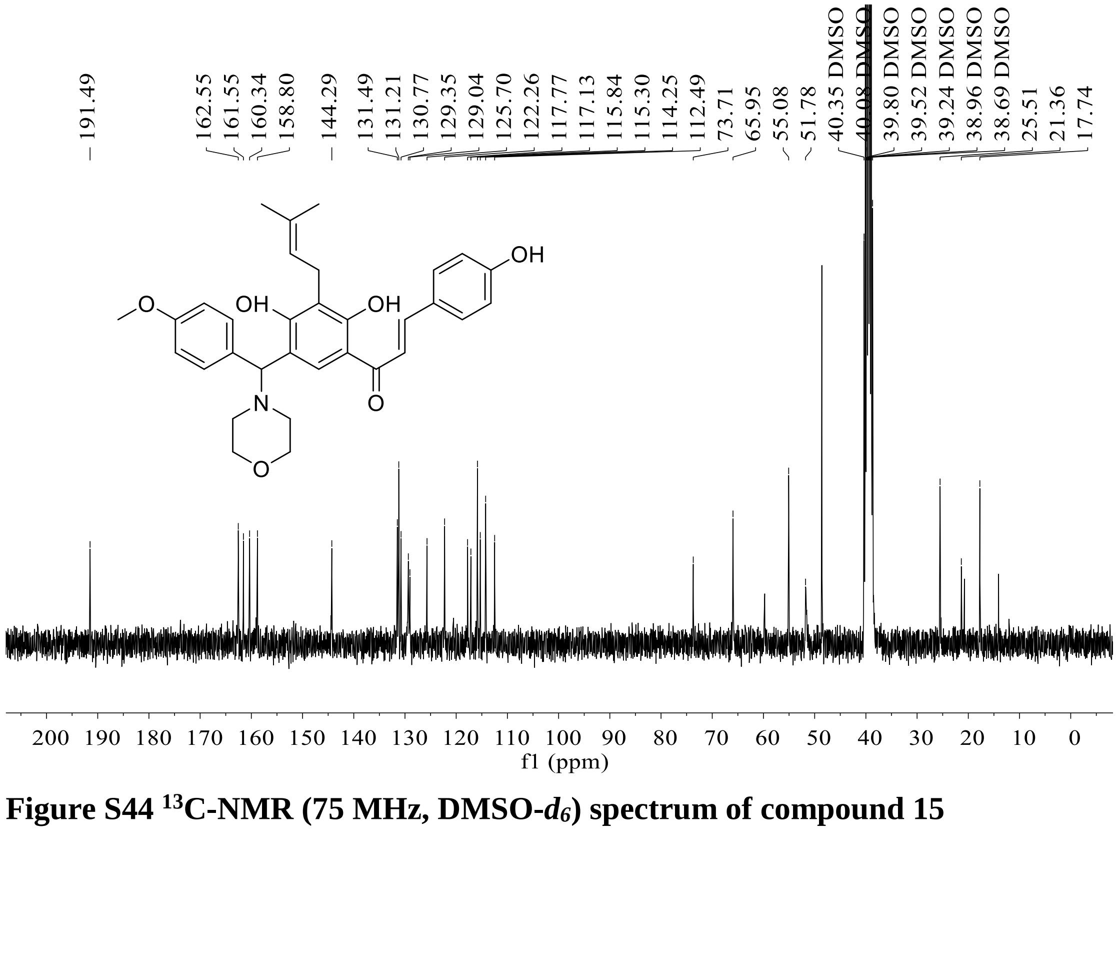

Figure S44 ^{13}C-NMR (75 MHz, DMSO-d_6) spectrum of compound 15

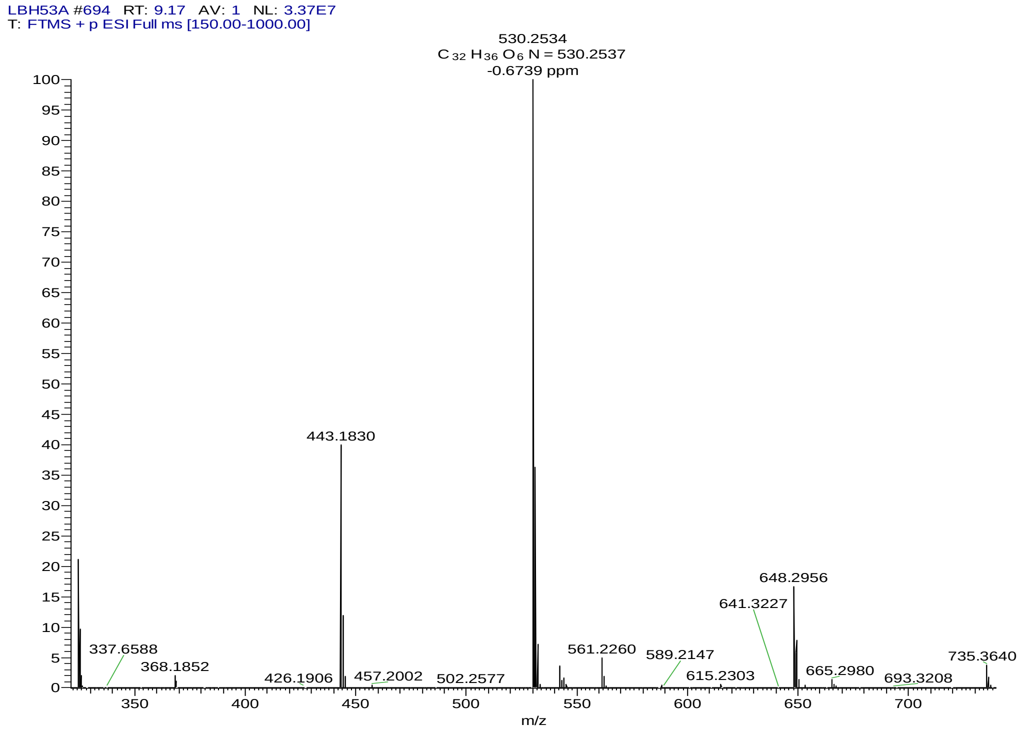

Figure S45 ESI-HRMS spectrum of compound 15

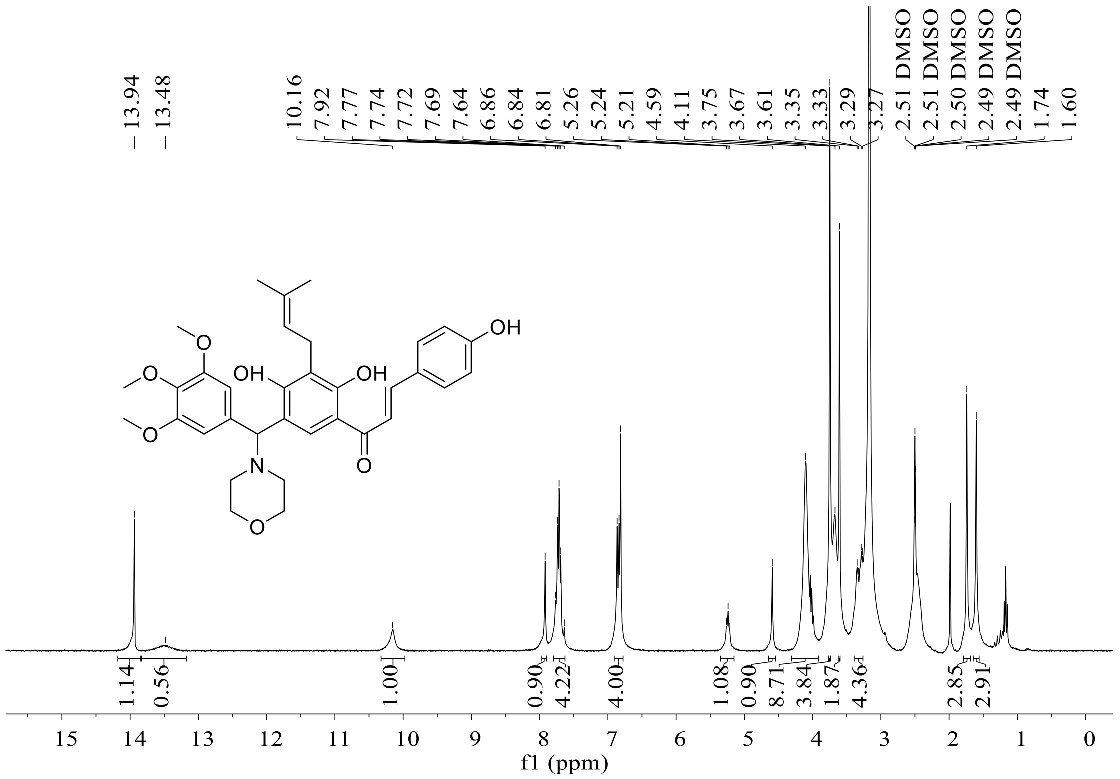

Figure S46 ^{1}H-NMR (300 MHz, DMSO-d_6) spectrum of compound 16

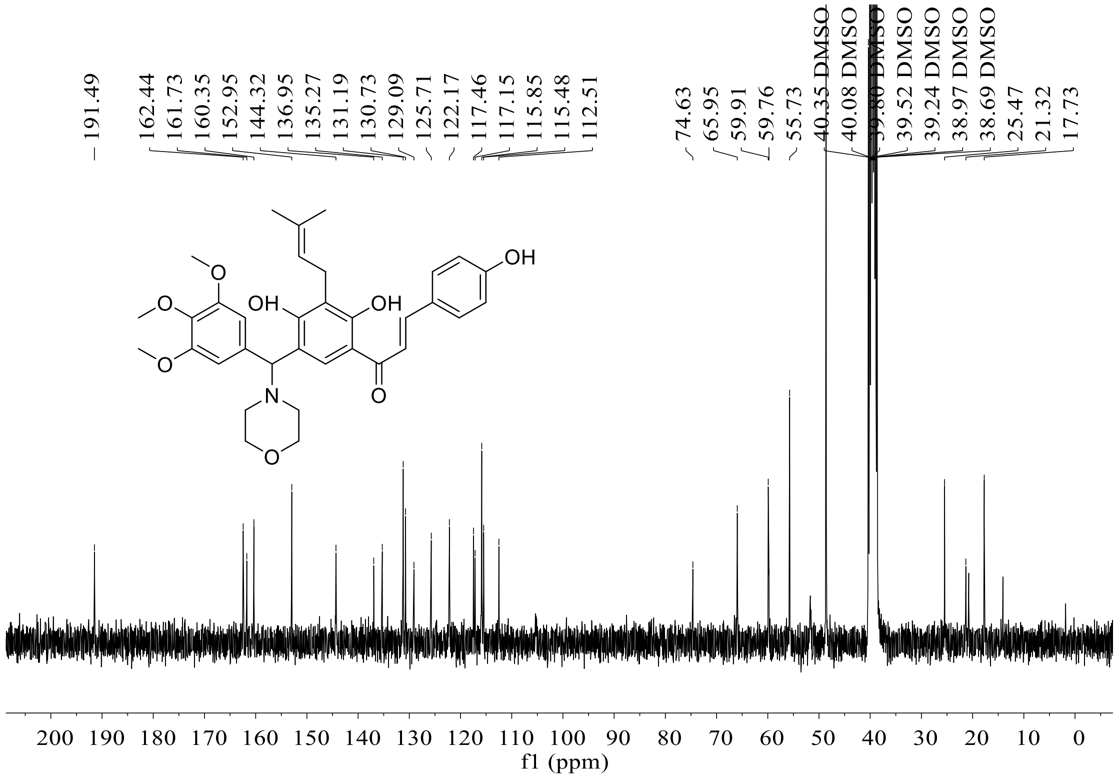

Figure S47 ^{13}C-NMR (75 MHz, DMSO-d_6) spectrum of compound 16

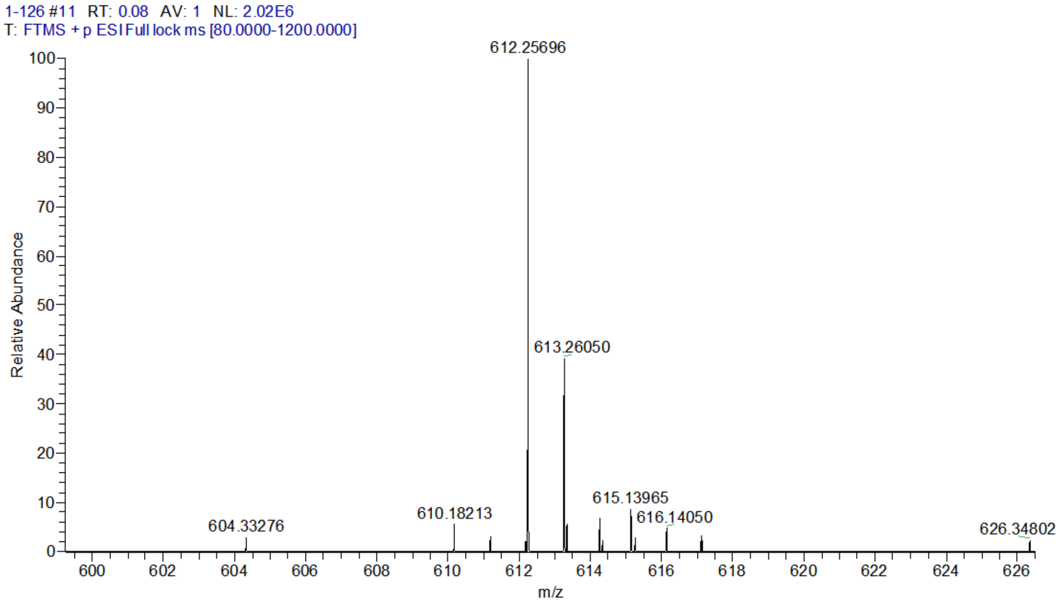

Figure S48 ESI-HRMS spectrum of compound 16

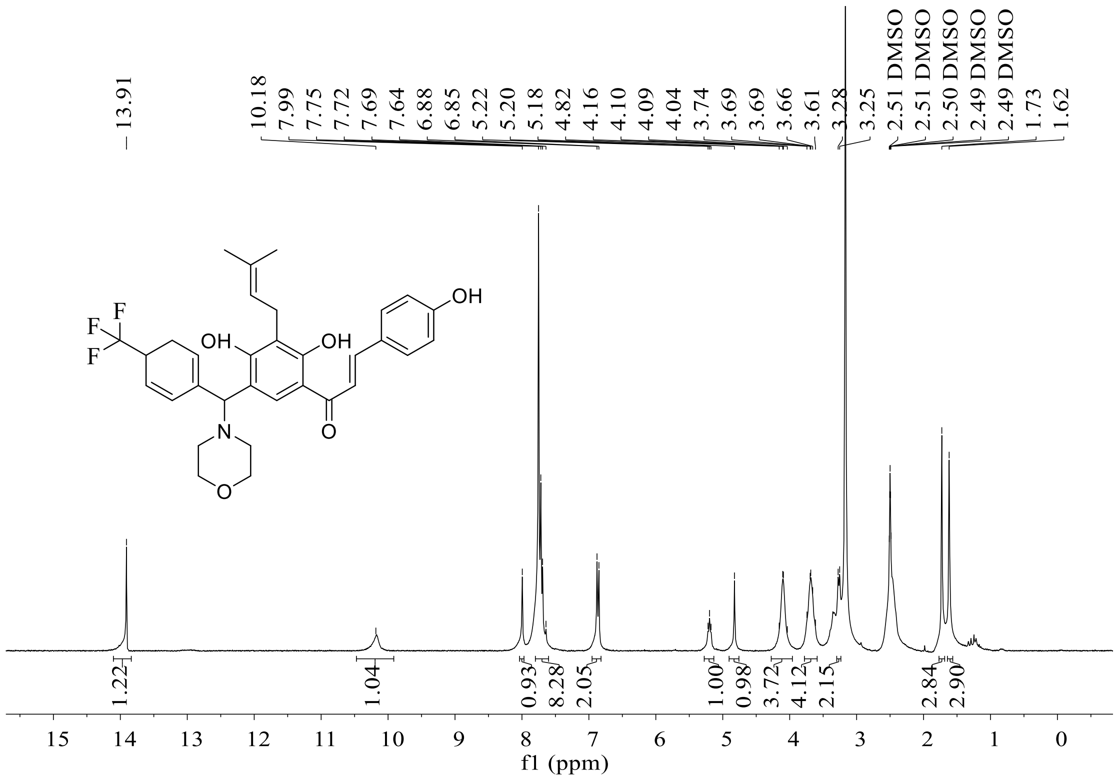

Figure S49 ^{1}H-NMR (300 MHz, DMSO-d_6) spectrum of compound 17

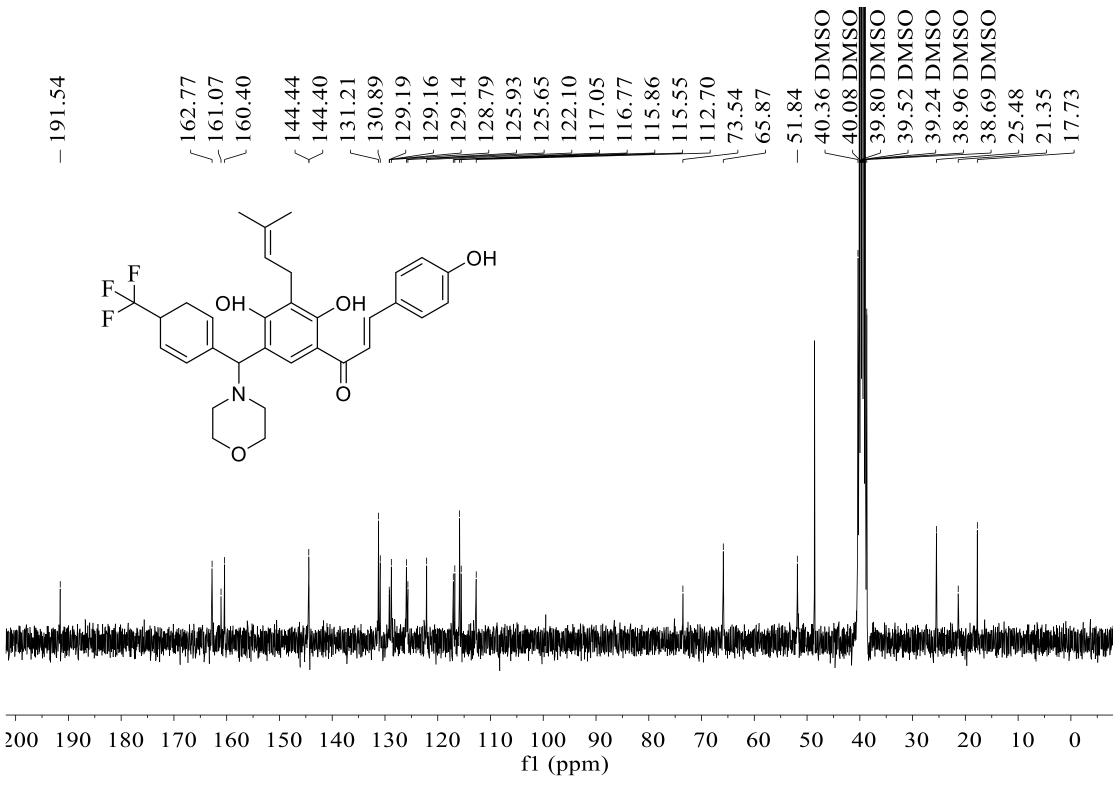

Figure S50 ^{13}C-NMR (75 MHz, DMSO-d_6) spectrum of compound 17

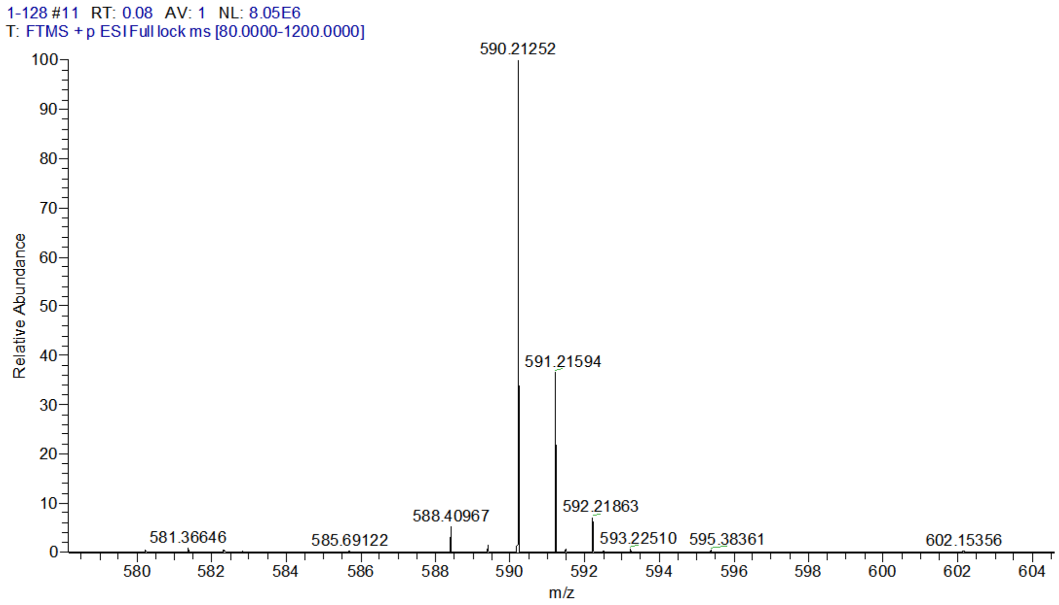

Figure S51 ESI-HRMS spectrum of compound 17
